# Evaluation of hybrid solvents featuring choline chloride-based deep eutectic solvents and ethanol as extractants for the liquid–liquid extraction of benzene from *n*-hexane: towards a green and sustainable paradigm

**DOI:** 10.1007/s13203-021-00282-y

**Published:** 2021-09-25

**Authors:** Mohammed Awwalu Usman, Olumide Kayode Fagoroye, Toluwalase Olufunmilayo Ajayi

**Affiliations:** Sustainable Process Technology Group, Process Systems Engineering Cluster, Department of Chemical and Petroleum Engineering, University of Lagos, Akoka, Yaba, 101017 Lagos Nigeria

**Keywords:** Glyceline, Ethaline, Reline, Ethanol, Distribution coefficient, Separation factor, Viscosity

## Abstract

Deep eutectic solvents (DESs) have high viscosities, but known to be mitigated by addition of suitable co-solvent. The effect of such co-solvent on the extraction efficiency of the hybrid solvent is hardly known. This study examined the effect of ethanol on three choline chloride-based DESs (glyceline, reline, and ethaline) by mixing each in turn with ethanol in various volume proportions. The hybrid solvents were evaluated for the extraction of benzene from *n*-hexane. Pseudo-ternary liquid–liquid equilibrium data were obtained using the refractive index method at 303 K and 1 atm for the systems, *n*-hexane (1) + benzene (2) + hybrid solvent (glyceline/ethanol, ethaline/ethanol, reline/ethanol) (3), and used to evaluate distribution coefficient (*D*) and selectivity (*S*). Furthermore, the physicochemical properties of the hybrid solvents were also determined. The results indicate increase in selectivity with increasing ethanol addition up to 50% and decrease with further addition. All hybrid solvents with 50% ethanol outperform sulfolane and are suitable replacement for same as green and sustainable extractant for aromatics from aliphatics. The glyceline + 50% ethanol emerged the overall best with 49.73% elevation in selectivity and 41.15% reduction in viscosity relative to the neat glyceline. The finding of this study is expected to fillip the drive for paradigm shift in petrochemical industries.

**Supplementary Information:**

The online version contains supplementary material available at 10.1007/s13203-021-00282-y.

## Introduction

Thermodynamic and transport properties are needed for the design and operation of process and products in diverse areas, more so in Chemical and Petroleum Engineering. A triangular collaboration between the academia, industries and software providers, to address challenges in this regard has been re-emphasized by a recent survey conducted on industry practitioners [1]. A key finding of the survey is the complementary roles of experimental data and model development which should not be jettisoned. For example, in a liquid–liquid extraction process, thermodynamic properties such as equilibrium data and derived parameters like selectivity (*S*) and distribution coefficient (*D*) enable determination of equipment size and solvent consumption rate. On the other hand, transport property such as viscosity fixes the hydrodynamics, mixing and flow issues. In the petrochemical industries, aromatic production from various sources like pyrolysis gasoline, reformate or naphtha accounts for 40 million metric tons of benzene, 40 million metric tons of xylenes and 20 million metric tons of toluene per annum globally [2]. A critical stage of this production process is the liquid–liquid extraction of the aromatics from the non-aromatic (or aliphatic) media. The choice of solvent with suitable values of thermodynamic and transport properties is crucial and bears overarching influence on the economic viability and sustainability of the process.

Deep eutectic solvents (DESs) are mixtures characterized by remarkable depressions in melting point relative to the constituents and having tunable physicochemical properties. It has continued to attract research attention in diverse area of application. A comprehensive review of DESs, fundamentals and applications can be found in the literature [3, 4]. More recent applications include biomass pre-treatment [5, 6], media for enzymatic hydrolysis [7, 8], platform for lipase extraction [9], biodiesel production and purification [10, 11], inhibiting shale hydration [12] and COVID-19 intervention [13]. In the specific area of solvent extraction, a few recent studies are worthy of review. Wojeicchowski et al. [14] explored the capacity of deep eutectic solvents to extract phenolics from rosemary leaves. The results indicate that the DES, choline chloride: 1,2-propanediol, at the optimal conditions (65 °C, liquid:solid ratio of 40:1 and 5.0% wt of water), achieved a 39–51% inhibition of antimicrobial activity of extract to all tested bacteria. Petracic et al. [15] investigated deep eutectic solvents as extractants to reduce the free fatty acid content of feedstocks for biodiesel production in a liquid–liquid extraction process. The results show that the acidity of waste animal fat was significantly reduced. Lemaoui et al. [16] studied the application of deep eutectic solvents as extractants in the simultaneous de-aromatization, desulfurization and denitrogenation of diesel in a liquid–liquid extraction process. The results showed that 100% removal of pyrrole and pyridine can be achieved in 2 stages. Rezaee et al. [17] investigated the use of deep eutectic solvents as extractants in the liquid–liquid extraction to remove dibenzothiophene from model fuel (*n*-octane). The result indicates significant removal of the sulfur-containing compound.

The superior performance of DESs as extractants for aromatics from aliphatic media over the conventional organic solvent (sulfolane) has been well reported in the literature. For example, Shekaari et al. [18] reported a maximum selectivity (*S*_max_) of 52.4197 for DES (choline chloride: diglycolamine, 1:5 molar ratio) as against 47.7704 for sulfolane in the extraction of benzene from *n*-hexane at 303.15 K. Similarly, Usman et al. [19] reported a high value for selectivity (*S*_max_ = 462.00) using glyceline (choline chloride:glycerol, 1:2 molar ratio) as extractant for separating benzene from *n*-hexane. In a related study using ASPEN simulation, Usman et al. [20] reported higher values of selectivity for glyceline (*S*_max_ = 378.283) and ethaline (*S*_max_ = 77.364) compared to sulfolane (*S*_max_ = 55.371) in the extraction of aromatics (benzene–toluene–xylene) from waste tire pyrolysis gasoline at 303.15 K and 1 atm. Further, using a mixture of glyceline and ethaline in the volume ratio 80:20, respectively, as extractant for separating benzene from *n*-hexane, Usman et al. [21] reported the selectivity value of 422.485. These studies eloquently speak to the superiority of DESs to sulfolane in terms of thermodynamic properties. In addition to the aforementioned experimental works, several molecular dynamic simulation studies have also been carried out in the evaluation of ionic liquids/deep eutectic solvents as extractants for separating aromatics from aliphatic hydrocarbons, with results that are in good agreement with experimental findings [22–24]. The green credentials of DESs, particularly glyceline, ethaline and reline, have been well established by various studies [25, 26].

However, the undesirable transport property (high viscosity) of DESs is a huge challenge to its industrial application. For example, the viscosities of glyceline, ethaline, reline and sulfolane are 342.12 cP, 38.52 cP, 667.28 cP [21], and 10.35 cP [27] at 303.15 K, respectively. Zheng et al. [28] posited that mixing of DESs with molecular solvents (volatile organic solvents) can help overcome the drawbacks of neat DESs and thus extend the practical or industrial application of DESs. In this regard, several studies have been conducted to mitigate the viscosity of DESs by blending with organic solvents. Some of the organic solvents explored include methanol [29], ethanol [30, 31], dimethyl sulfoxide [32]. The considerably lower viscosities of the organic solvents in comparison with DESs or ionic liquids (ILs) suggest that the former should have a thinning effect on the latter. This was corroborated in the aforementioned studies as viscosities of the mixed solvents plummet further with increase in the proportion of organic solvent. Ethanol is expected to exert viscosity reduction on DESs giving its much lower viscosity of 0.983 cP.

Traditionally, ethanol is produced from biomass in a production chain that encompasses some or all of the following steps: pre-treatment, hydrolysis, enzymatic fermentation and purification, depending on the feedstock. The purification stage, conventionally done by distillation, is characterized by high-energy consumption and inefficiency. Azeotropy of the ethanol–water mixture is an additional contributory factor. These tend to detract from the green and sustainable indices of the process and that of ethanol. Thankfully, there are emerging technologies that ensure drastic knockdown on energy consumption and guarantee improved efficiency of the distillation process [33]. Other interventions focused on alternative separation methods, such as liquid–liquid extraction [34, 35] and adsorption [36, 37]. All these restore confidence in the green and sustainable credentials of ethanol.

Ethanol can therefore be rightly classified as green and sustainable co-solvent to add to DESs for viscosity reduction. However, the effect of such addition on the thermodynamic properties (extraction performance for aromatics) of the resulting hybrid solvent has not been previously explored, to the best of our knowledge. This study therefore seeks to bridge the gap by mixing glyceline, ethaline and reline in all volume proportions with ethanol to form three categories of hybrid solvents (glyceline + ethanol, ethaline + ethanol, and reline + ethanol). These hybrid solvents were then evaluated as extractants for the extraction of benzene from *n*-hexane. The extraction efficiency is assessed using the thermodynamic parameters, benzene distribution coefficient (*D*) and selectivity factor (*S*). The physicochemical properties (density, viscosity and refractive index) of the extractants were also measured.

## Experimental

### Materials

Benzene, *n*-hexane, Choline chloride, ethylene glycol, urea, glycerol and ethanol were purchased from Sigma-Aldrich (Germany) with a mass fraction purity higher than 0.98. All chemicals were used as received without further purification and they were stored in a desiccator in their original tightly closed bottles. Table 1 shows the chemicals, CAS number and purity.

**Table 1:** Chemicals used in this study

Component	Supplier	CAS reg. no.	Mass fraction purity (%)
Benzene	Sigma-Aldrich	71-43-2	≥ 99.5
*n*-Hexane	Sigma-Aldrich	110-54-3	≥ 99
Ethanol	Sigma-Aldrich	64-17-5	≥ 99
Choline chloride	Sigma-Aldrich	67-48-1	≥ 98
Ethylene glycol	Sigma-Aldrich	107-21-1	≥ 99
Glycerol	Sigma-Aldrich	56-81-5	≥ 99
Urea	Sigma-Aldrich	57-13-6	≥ 99

### Preparation of deep eutectic solvents and hybrid solvents

Three deep eutectic solvents were prepared in this study, namely ethaline (choline chloride and ethylene glycol), glyceline (choline chloride and glycerol) and reline (choline chloride and urea). The quaternary ammonium salt (choline chloride) was used as the hydrogen bond acceptor while urea, glycerol and ethylene glycol serve as the hydrogen bond donors in the molar ratio 1:2, respectively. The detailed protocol for preparing these DESs is explained in our previous articles [19, 21]. The hybrid solvents of these DESs were then prepared by mixing each DES with ethanol in varying volume proportion and named appropriately. For example, G95Et5 means 95% glyceline and 5% ethanol; E80Et20 means 80% ethaline and 20% ethanol; R60Et40 means 60% reline and 40% ethanol. A total of fifty-seven (57) DES–ethanol hybrid solvents were so prepared and used for this study in addition to the three neat DESs (E100, G100, and R100) and pure ethanol (Et100). Overall, there were sixty-one (61) solvents or extractants used for this study. The water contents in each DESs and hybrid solvents were determined by the method described in our previous article [19], the mass fraction was ≤ 0.0003 for all studied solvents.

### Extraction and determination of LLE data

The 61 solvents were each investigated for their extraction capacity; each was used as an extractant in the separation of *n*-hexane + benzene mixture. The extraction process was done on a bench scale as described in our articles [19, 21]. Measured volume of *n*-hexane + benzene mixture (feed) was contacted with hybrid solvent or solvent in 250 ml beaker. The extraction runs were carried out in a vessel, the temperature was controlled by a water bath at 303 K. After bringing the feed to extraction temperature, the solvent was added at the same temperature (according to the predetermined ratio). A rotating stainless steel shaft was used for mixing the feed and solvent at a controlled degree of mixing of 500 rpm. The extraction was carried out for a predetermined mixing time of 1 h and the mixture was left to separate into a raffinate phase (*n*-hexane-rich phase) at the top and an extract phase (solvent-rich phase) at the bottom for a predetermined settling time of 4 h. The extract was then separated and the equilibrium compositions of the phases were determined via refractive index measurement. All experiments were duplicated and average values reported.

### Determination of physicochemical properties

Densities were measured using a density tube meter, the viscosities were measured with a Brooksfield DV2T viscometer. This viscometer was calibrated with distilled water. Viscosity of the samples (*η*) was obtained under the following conditions; the flow time of 60 s was used to measure the flow time in the hybrid solvents or solvent, *T* = 303 K and a speed of 50 rpm. The estimated uncertainty of the experimental viscosity was ± 0.02 cP. The refractive index was measured with a digital refractometer (ATAGO DRA1, Japan) with an uncertainty of ± 0.001.

The analytical balance (AND, GR202, Japan) with the precision of ± 0.0001 g was used for the preparation of mixtures in molar basis. The studied hybrid solvents were prepared in well-sealed glass vials to avoid contamination or mixture evaporation. Measurements were done continually after the mixtures preparation. The standard uncertainty of solubility is 0.0014 and density is 0.001 g/cm^3^.

### Determination of performance parameters

The performances of the extractants were evaluated based on two metrics, namely benzene distribution coefficient (*D*) and selectivity (*S*) as defined mathematically in Eqs. (1–2).

1 $$D = \frac{{x_{23} }}{{x_{21} }},$$

2 $$S = \frac{{x_{23} \cdot x_{11} }}{{x_{21} \cdot x_{13} }},$$

where $x_{23}$ is the mole fraction of benzene in the extract (hybrid solvent) phase, $x_{21}$ is the mole fraction of benzene in the raffinate (*n*-hexane) phase, $x_{13}$ is the mole fraction of *n*-hexane in the extract (hybrid solvent) phase and $x_{11}$ is the mole fraction of *n*-hexane in the raffinate (hexane) phase.

## Results and discussion

In this section, the results of the experimental studies and subsequent analyses are presented and thoroughly discussed. The first sub-section presents and explains the performance of the neat ethanol (Et100, EtOH) while the second subsection presents and discusses the performances of the hybrid solvents of DESs and ethanol under the banner of the three pairs (ethaline/ethanol, glyceline/ethanol, and reline/ethanol). The physicochemical properties of the sixty-one (61) solvents or extractants are then presented in the third sub-section. The section is concluded with a general discussion and comparison of the studied extractants in the fourth subsection.

### Performance of neat ethanol (Et100, EtOH)

This sub-section presents the liquid–liquid equilibria data/ternary diagram, distribution coefficients and selectivities for the extraction of benzene from *n*-hexane using neat ethanol as the extractant.

#### LLE data and tie lines

**Fig. 1:**
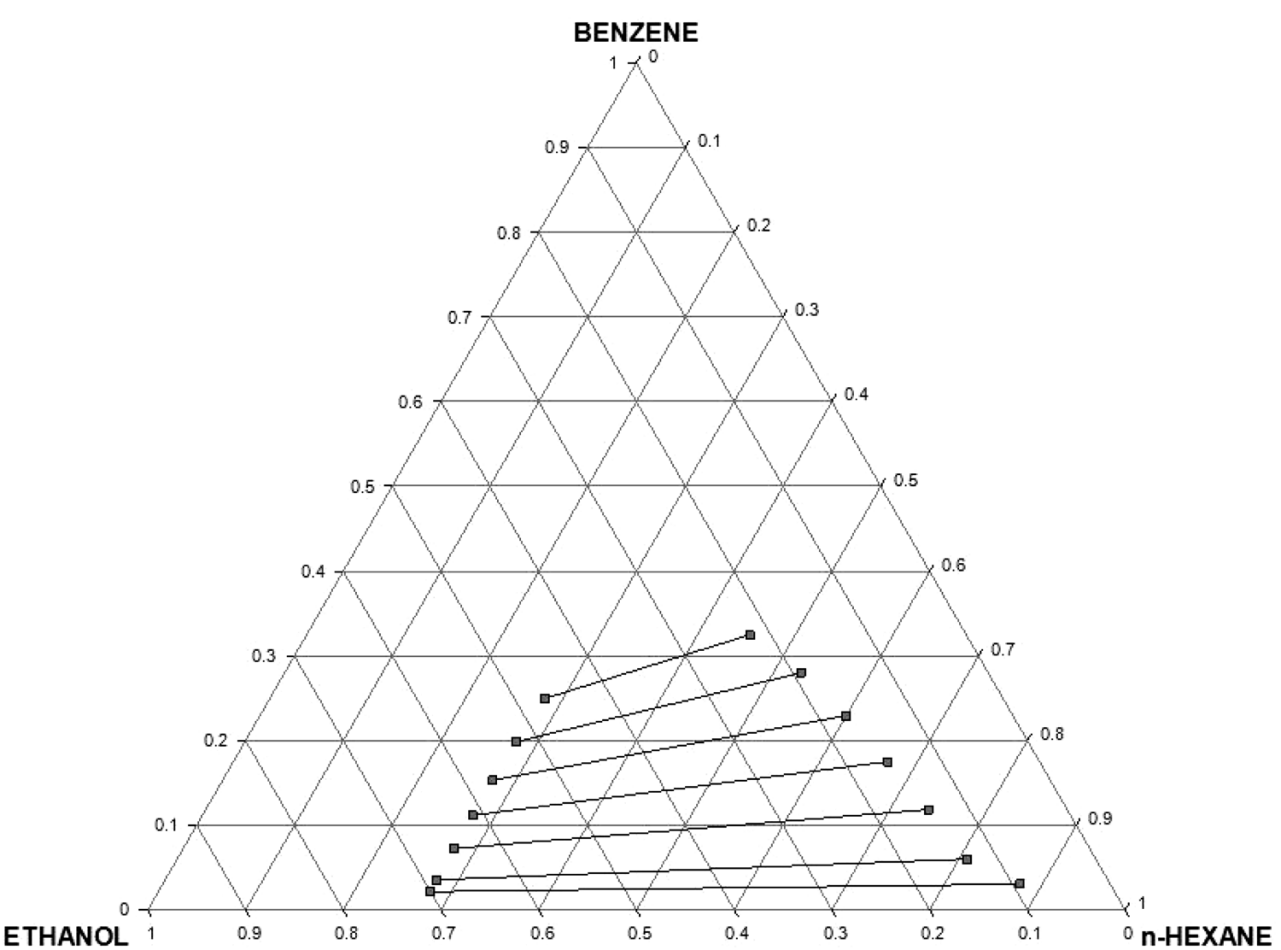
Experimental tie lines for the system *n-*hexane + benzene + ethanol (Et100, EtOH) at temperature 303 K and atmospheric pressure

Table S1 (Supplementary information) shows the liquid–liquid equilibrium data for the ternary system *n*-hexane (1) + benzene (2) + ethanol (3) at 303 K and 1 atm. These data are plotted in a ternary diagram as shown in Fig. 1. The biphasic region is clearly narrow, indicating a limited operation window for liquid–liquid extraction. The pair of *n*-hexane and ethanol shows partial miscibility to warrant recovery of ethanol from the raffinate with the attendant energy expenditure and cost implication. It is instructive to mention that this ternary diagram provides justification for the blending of gasoline with ethanol (10% EtOH or 15% EtOH) as currently practiced in some countries of the world as such blend lie within the single-phase region. It also provides limit for such blending, as any blend that falls within the two-phase region would not be acceptable since it compromises fuel function.

#### Distribution coefficients and selectivities

**Fig. 2:**
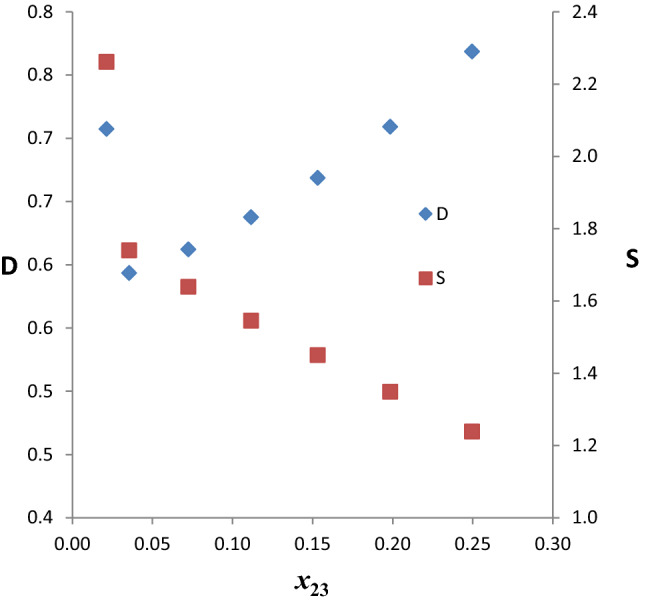
Benzene distribution coefficients and selectivities as a function of benzene composition in the extract phase for the system *n-*hexane + benzene + EtOH at temperature 303 K and atmospheric pressure

The benzene distribution coefficients (D) and selectivities (S) for the ternary system *n*-hexane (1) + benzene (2) + ethanol (3) at 303 K are presented in Table S1 and plotted in Fig. 2 as a function of benzene composition in the extract phase. The values of *D* vary from 0.593 to 0.769 while those of *S* vary from 1.238 to 2.261 as the benzene composition in the extract phase increases from 0.0215 to 0.2497. In a related study, Gramajo et al. [38] reported liquid–liquid equilibrium data for *n*-hexane + benzene + methanol system at 278.15 K in mass fractions from which evaluated values of *D* range from 0.26 to 0.74 while *S* ranges from 1.31 to 4.44. Thus, ethanol is a better extractant compared with methanol. However, it is clear that ethanol is a poor extractant for benzene when its *S* values are compared with those of other organic solvents: sulfolane (2.7963–47.7704), *N*-formylmorpholine (2.8551–21.8382), and diglycolamine (2.1985–47.1515) as reported by Shekaari et al. [18] at 303.15 K.

### Performance of the hybrid solvents: DESs and ethanol

The extraction performance of the three hybrid solvent categories: ethaline/ethanol, glyceline/ethanol and reline/ethanol is hereby presented.

#### Ethaline + ethanol (E100–E5Et95)

There were twenty (20) extractants investigated in this study involving ethaline, including the neat-ethaline (ChCl:EG 100%; E100) and nineteen (19) hybrid solvents of ethaline and ethanol in volume proportions of ethanol ranging from 5 to 95% in incremental steps of 5% (E95Et5–E5Et95). This sub-section presents the liquid–liquid equilibria data, ternary diagrams, distribution coefficients and selectivities for the extraction of benzene from *n*-hexane using these extractants.

##### LLE data and tie lines

**Fig. 3:**
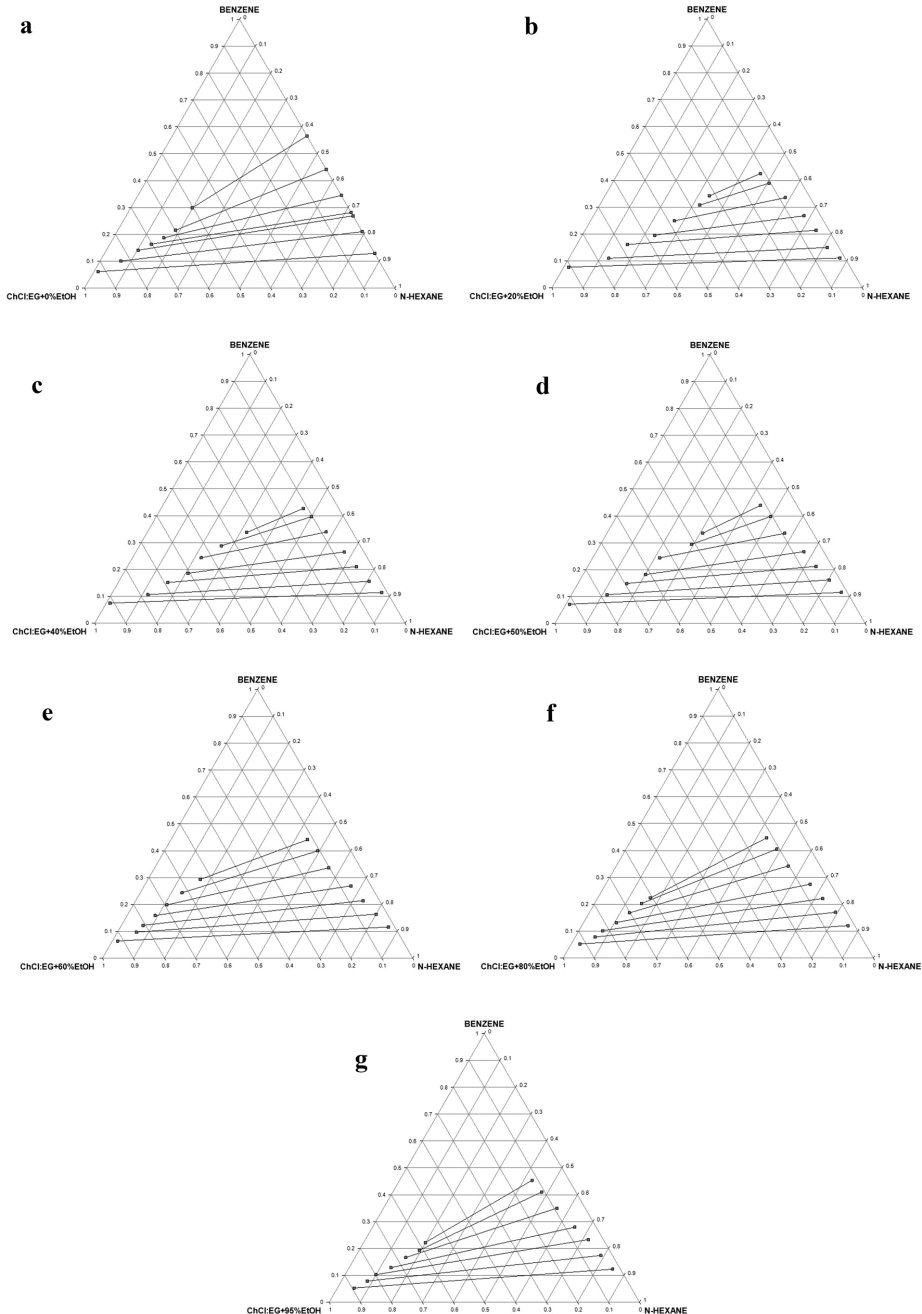
**a** Experimental ternary diagram/tie lines for the *n-*hexane + benzene + E100 (ChCl:EG + 0%EtOH) at temperature 303 K and atmospheric pressure. **b** Experimental ternary diagram/tie lines for the *n-*hexane + benzene + E80Et20 (ChCl:EG + 20%EtOH) at temperature 303 K and atmospheric pressure. **c**: Experimental ternary diagram/tie lines for the *n-*hexane + benzene + E60Et40 (ChCl:EG + 40%EtOH) at temperature 303 K and atmospheric pressure. **d** Experimental ternary diagram/tie lines for the *n-*hexane + benzene + E50Et50 (ChCl:EG + 50%EtOH) at temperature 303 K and atmospheric pressure. **e** Experimental ternary diagram/tie lines for the *n-*hexane + benzene + E40Et60 (ChCl:EG + 60%EtOH) at temperature 303 K and atmospheric pressure. **f** Experimental ternary diagram/tie lines for the *n-*hexane + benzene + E20Et80 (ChCl:EG + 80%EtOH) at temperature 303 K and atmospheric pressure. **g** Experimental ternary diagram/tie lines for the *n-*hexane + benzene + E5Et95 (ChCl:EG + 95%EtOH) at temperature 303 K and atmospheric pressure

Table S2 presents the comprehensive experimental LLE data for the pseudo-ternary systems of *n*-hexane (1) + benzene (2) + solvent (E100–E5Et95) (3). For the sake of brevity, the ternary diagrams/tie lines for seven (7) of the twenty (20) systems are shown in Fig. 3a–g, while the remaining ones are presented in Figure S1 (a–m). These seven are considered typical of behavior pattern of the lot. At 0% EtOH, as shown in Fig. 3a, there is no miscibility between ethaline and *n*-hexane, thus no DES in the raffinate phase thereby obviating the need for solvent recovery from this phase. As the % EtOH addition increases in the hybrid solvent, the miscibility improves (Fig. 3b–g). The two-phase region also decreases as the volume proportion of EtOH increases from 0% in Fig. 3a to 95% in Fig. 3g. Type 1 behavior according to Treybal’s classification was exhibited by all the hybrid solvents [39]. The highly polar nature of the studied DES may be responsible for its immiscibility with *n*-hexane. Finally, the positive slopes of tie lines show that benzene solubility in *n*-hexane is higher than its solubility in the solvents.

##### Distribution coefficients and selectivities

**Fig. 4:**
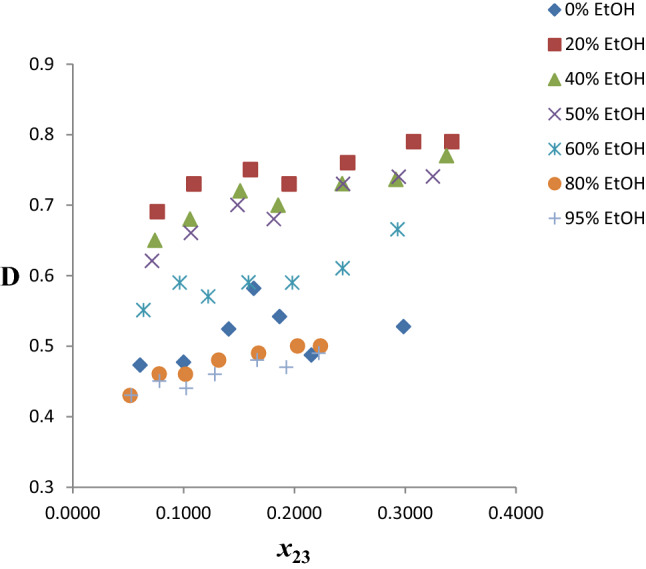
Benzene distribution coefficients as a function of benzene composition in the extract phase for the system *n-*hexane + benzene + ethaline/EtOH at temperature 303 K and atmospheric pressure

The benzene distribution coefficients (D) obtained for the twenty pseudo-ternary systems of *n*-hexane (1) + benzene (2) + hybrid solvent (E100–E5Et95) (3) are presented in Table S2. Figure 4 shows a plot of *D* versus benzene composition in the extract phase for the seven chosen systems. The *D* values increase as % EtOH increase from 0 to 20%, but drop subsequently, though still higher than the values at 0% EtOH, up to 60% EtOH. The values of *D* beyond 60% EtOH are less than the values at 0% EtOH.

**Fig. 5:**
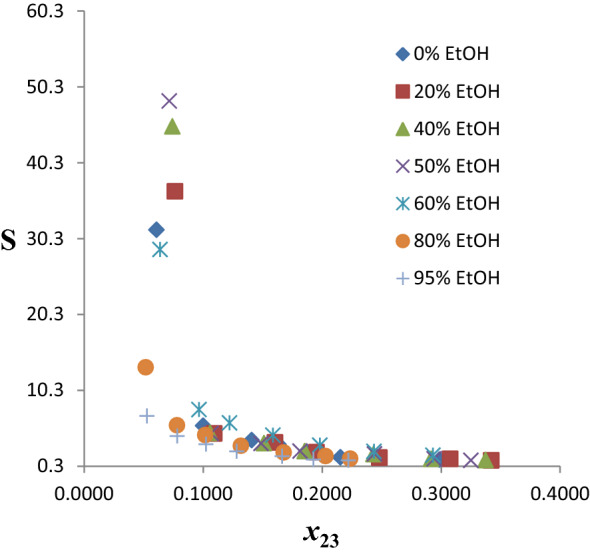
Selectivities as a function of benzene composition in the extract phase for the system *n-*hexane + benzene + ethaline/EtOH at temperature 303 K and atmospheric pressure

The selectivity values for the twenty pseudo-ternary systems are shown in Table S2 and those of the chosen seven are plotted in Fig. 5. Generally, the *S* values decrease with increasing composition of benzene in the extract phase for the seven extractants. Addition of EtOH significantly improved the selectivity of the hybrid solvents relative to neat-ethaline up to 50% and thereafter it decreased very sharply particularly beyond 60% EtOH. The maximum value of *S* is in the following decreasing order: E50Et50 (50% EtOH, S = 48.421) $\succ$ E60Et40 (40% EtOH, *S* = 45.097) $\succ$ E80Et20 (20% EtOH, *S* = 36.539) $\succ$ Et100 (0% EtOH, *S* = 31.440) $\succ$ E40Et60 (60% EtOH, *S* = 28.834) $\succ$ E20Et80 (80% EtOH, *S* = 13.323) $\succ$ E5Et95 (95% EtOH, *S* = 6.901). There is a 54.01% increase in the maximum value of *S* at 50% MeOH relative to the neat-ethaline. Thus, EtOH impart elevation in selectivity to ethaline up to 50% addition but a sharp attenuation beyond. Also worthy of note is the convergence of *S* values beyond 10% mole fraction of benzene in the extract irrespective of % EtOH. This signifies selectivity becomes insensitive to EtOH addition when the composition of benzene in the extract phase goes above 10%. Similar trend occurred in the extraction of benzene from *n*-hexane using a binary mixed DES (glyceline/ethaline) as extractant [21]. Generally, selectivity tends to unity as the tie line tends toward the plait point, where the distribution coefficient of solute and other components of the ternary mixture becomes unity. Thus, the observed convergence of *S* values can be attributed to the inability of the hybrid solvents to discriminate beyond 10% mole fraction of benzene as the *S* value has become very low.

#### Glyceline + ethanol (G100–G5Et95)

There were twenty (20) extractants investigated in this study involving glyceline, including the neat glyceline (ChCl:Gly 100%; G100) and nineteen (19) hybrid solvents of glyceline and ethanol in volume proportions of ethanol ranging from 5 to 95% in incremental steps of 5% (G95M5–G5M95). This sub-section presents the liquid–liquid equilibria data, ternary diagrams, distribution coefficients and selectivities for the extraction of benzene from *n*-hexane using these extractants.

##### LLE data and tie lines

**Fig. 6:**
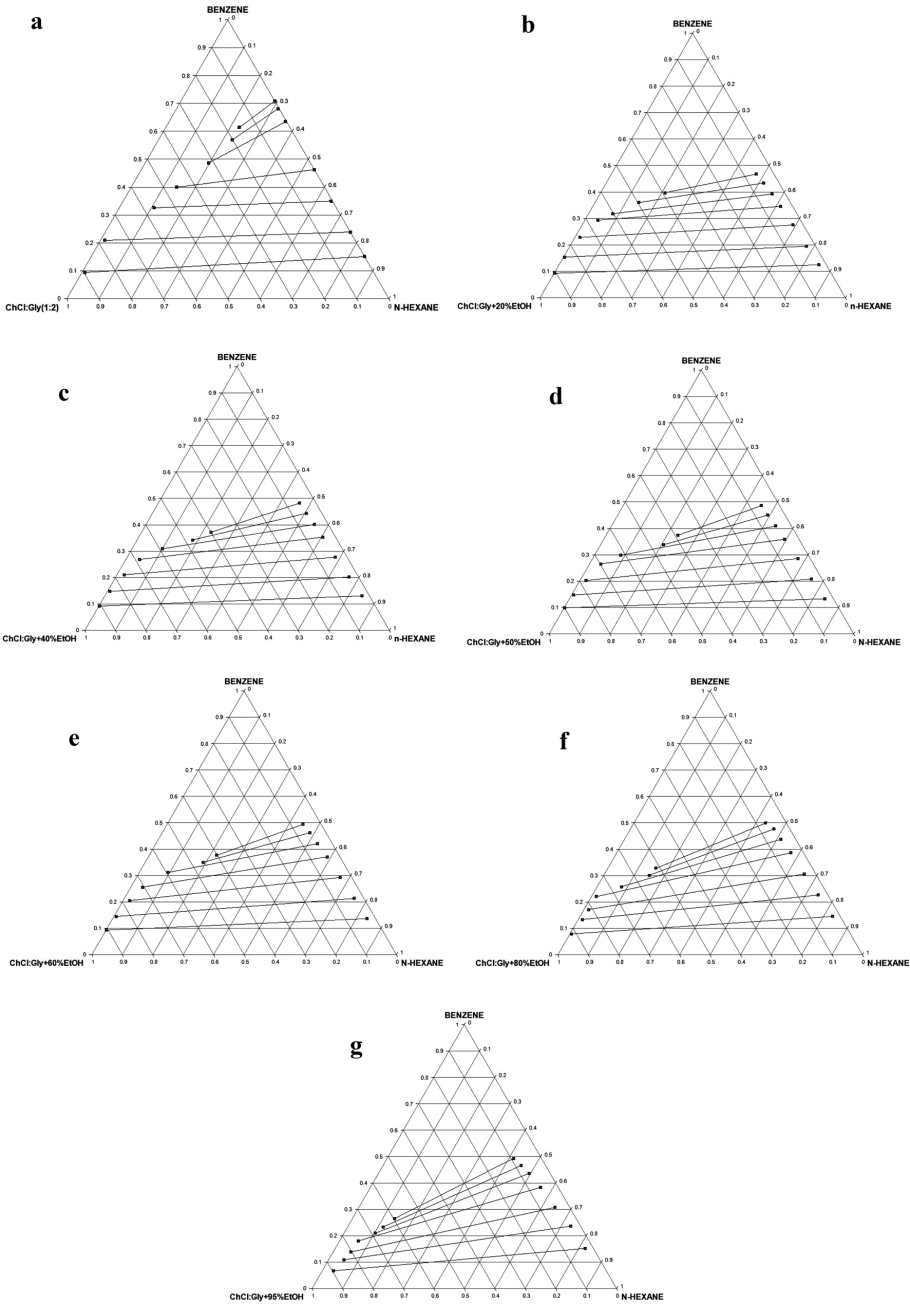
**a** Experimental ternary diagram/tie lines for the *n-*hexane + benzene + G100 (ChCl:Gly + 0%EtOH) at temperature 303 K and atmospheric pressure. **b** Experimental ternary diagram/tie lines for the *n-*hexane + benzene + G80Et20 (ChCl:Gly + 20%EtOH) at temperature 303 K and atmospheric pressure. **c** Experimental ternary diagram/tie lines for the *n-*hexane + benzene + G60Et40 (ChCl:Gly + 40%EtOH) at temperature 303 K and atmospheric pressure. **d** Experimental ternary diagram/tie lines for the *n-*hexane + benzene + G50Et50 (ChCl:Gly + 50%EtOH) at temperature 303 K and atmospheric pressure. **e** Experimental ternary diagram/tie lines for the *n-*hexane + benzene + G40Et60 (ChCl:Gly + 60%EtOH) at temperature 303 K and atmospheric pressure. **f** Experimental ternary diagram/tie lines for the *n-*hexane + benzene + G20Et80 (ChCl:Gly + 80%EtOH) at temperature 303 K and atmospheric pressure. **g** Experimental ternary diagram/tie lines for the *n-*hexane + benzene + G5Et95 (ChCl:Gly + 95%EtOH) at temperature 303 K and atmospheric pressure

Table S3 presents the comprehensive experimental LLE data for the pseudo-ternary systems of *n*-hexane (1) + benzene (2) + solvent (G100–G5Et95) (3). As earlier explained, the ternary diagrams/tie lines for seven (7) of the twenty (20) systems are shown in Fig. 6a–g, while the remaining ones are presented in Figure S2a–m. These seven are considered typical of behavior pattern of the lot. At 0% EtOH, as shown in Fig. 6a, there is no miscibility between glyceline and *n*-hexane, thus no DES in the raffinate phase, thereby obviating the need for solvent recovery from this phase. As the % EtOH increases in the hybrid solvent, the miscibility improves (Fig. 6b–g). The two-phase region also decreases as the volume proportion of EtOH increases from 0% in Fig. 6a to 95% in Fig. 6g. Type 1 behavior according to Treybal’s classification was exhibited by all the extractants [39]. The highly polar nature of the studied DES may be responsible for its immiscibility with *n*-hexane. The biphasic region decreases with increase in % MeOH. Finally, the positive slopes of tie lines show that benzene solubility in *n*-hexane is higher than its solubility in the solvent.

##### Distribution coefficients and selectivities

**Fig. 7:**
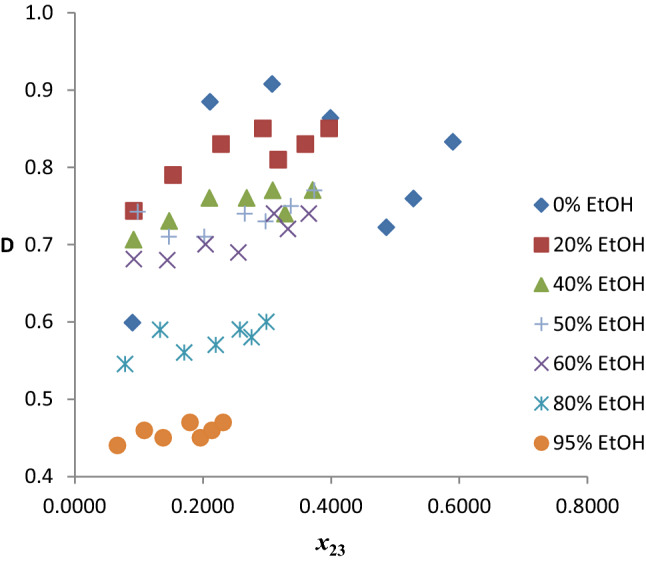
Benzene distribution coefficients as a function of benzene composition in the extract phase for the system *n-*hexane + benzene + glyceline/EtOH at temperature 303 K and atmospheric pressure

The benzene distribution coefficients (*D*) obtained for the twenty pseudo-ternary systems of *n*-hexane (1) + benzene (2) + solvent (G100–G5Et95) (3) are presented in Table S3. Figure 7 shows a plot of *D* versus benzene composition in the extract phase for the seven chosen systems. The *D* values of the hybrid solvents from 0 to 60% EtOH are generally ≥ 0.6 at all compositions of benzene in the extract phase. Attenuation of *D* values only becomes significant with EtOH content beyond 60%.

**Fig. 8:**
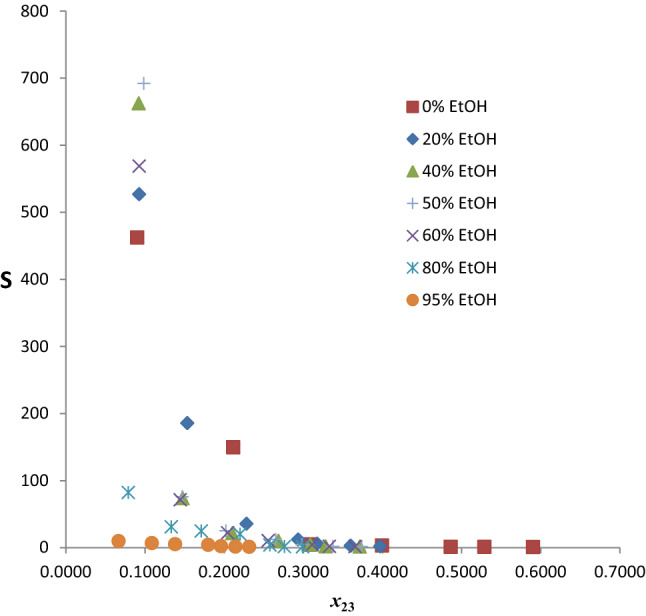
Selectivities as a function of benzene composition in the extract phase for the system *n-*hexane + benzene + glyceline/EtOH at temperature 303 K and atmospheric pressure

The selectivity values for the twenty pseudo-ternary systems are shown in Table S3 and those of the chosen seven are plotted in Fig. 8. Generally, the *S* values decrease with increasing composition of benzene in the extract phase for the seven extractants. Addition of EtOH significantly improved the selectivity of the hybrid solvent relative to neat glyceline up to 60% and thereafter it decreased very sharply. There is a clear enhancement in the *S*_max_ up to 60%, even though the highest value occurs at 50% EtOH. The maximum value of *S* is in the following decreasing order: G50Et50 (50% EtOH, *S* = 692.070) $\succ$ G60Et40 (40% EtOH, *S* = 662.413) $\succ$ G40Et60 (60% EtOH, *S* = 568.774) $\succ$ G80Et20 (20% EtOH, *S* = 526.833) $\succ$ G100 (0% EtOH, *S* = 462.219) $\succ$ G20Et80 (80% EtOH, *S* = 82.165) $\succ$ G5Et95 (95% EtOH, *S* = 9.787). There is a 49.73% increase in the maximum value of *S* at 50% EtOH relative to the neat glyceline. The decrease in *S*_max_ from 60 to 80% is profoundly sharp, a drop of 85.55%, signifying sharp attenuation beyond 60% EtOH. Also worthy of note is the convergence of *S* values beyond 20% mole fraction of benzene in the extract irrespective of % EtOH. This signifies selectivity becomes insensitive to EtOH addition when the composition of benzene in the extract phase goes above 20%. Similar trend occurred in the extraction of benzene from *n*-hexane using a binary mixed DES (glyceline/reline) as extractant [21]. Generally, selectivity tends to unity as the tie line tends toward the plait point, where the distribution coefficient of solute and other components of the ternary mixture becomes unity. Thus, the observed convergence of *S* values can be attributed to the inability of the hybrid solvents to discriminate beyond 20% mole fraction of benzene as the *S* value has become very low.

#### Reline + ethanol (R100–R5Et95)

There were twenty (20) extractants investigated in this category, including the neat-reline (ChCl:Ur 100%; R100) and nineteen (19) hybrid solvents of reline and ethanol in volume proportions of ethanol ranging from 5 to 95% in incremental steps of 5% (R95Et5–R5Et95). This sub-section presents the liquid–liquid equilibria data, ternary diagrams, distribution coefficients and selectivities for the extraction of benzene from *n*-hexane using these extractants.

##### LLE data and tie line

**Fig. 9:**
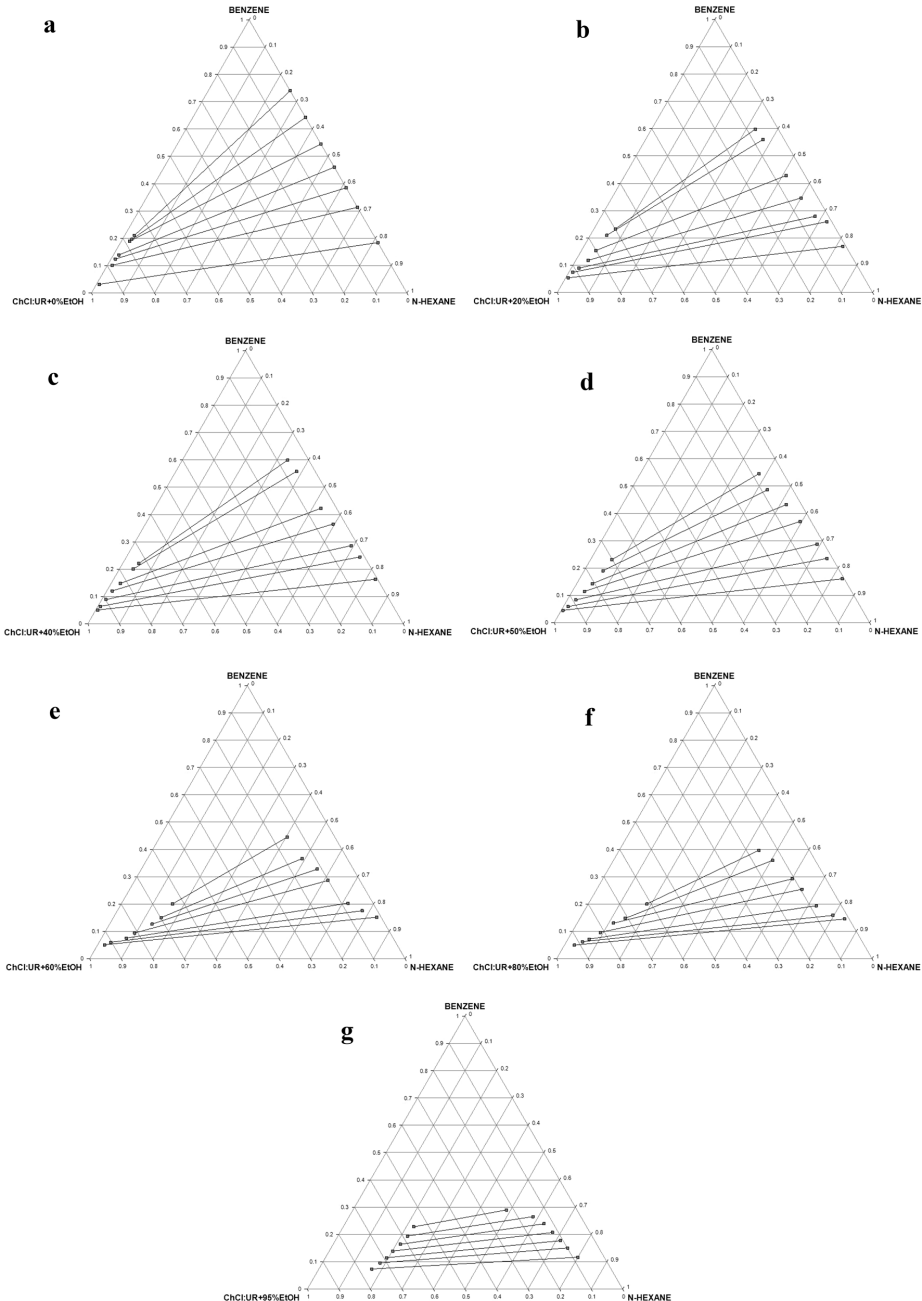
**a** Experimental ternary diagram/tie lines for the *n-*hexane + benzene + R100 (ChCl:Ur + 0%EtOH) at temperature 303 K and atmospheric pressure. **b** Experimental ternary diagram/tie lines for the *n-*hexane + benzene + R80Et20 (ChCl:Ur + 20%EtOH) at temperature 303 K and atmospheric pressure. **c** Experimental ternary diagram/tie lines for the *n-*hexane + benzene + R60Et40 (ChCl:Ur + 40%EtOH) at temperature 303 K and atmospheric pressure. **d** Experimental ternary diagram/tie lines for the *n-*hexane + benzene + R50Et50 (ChCl:Ur + 50%EtOH) at temperature 303 K and atmospheric pressure. **e** Experimental ternary diagram/tie lines for the *n-*hexane + benzene + R40Et60 (ChCl:Ur + 60%EtOH) at temperature 303 K and atmospheric pressure. **f** Experimental ternary diagram/tie lines for the *n-*hexane + benzene + R20Et80 (ChCl:Ur + 80%EtOH) at temperature 303 K and atmospheric pressure. **g** Experimental ternary diagram/tie lines for the *n-*hexane + benzene + R5Et95 (ChCl:Ur + 95%EtOH) at temperature 303 K and atmospheric pressure

Table S4 presents the comprehensive experimental LLE data for the pseudo-ternary systems of *n*-hexane (1) + benzene (2) + hybrid solvent (R100–R5Et95) (3). As earlier explained, the ternary diagrams/tie lines for seven (7) of the twenty (20) systems are shown in Fig. 9a–g, while the remaining ones are presented in Figure S3a–m. These seven are considered typical of behavior pattern of the lot. At 0% EtOH, as shown in Fig. 9a, there is no miscibility between glyceline and *n*-hexane, thus no DES in the raffinate phase, thereby obviating the need for solvent recovery from this phase. As the % EtOH addition increases in the hybrid solvent, the miscibility improves (Fig. 9b–g). The two-phase region also decreases as the volume proportion of EtOH increases from 0% in Fig. 9a to 95% in Fig. 9g. Type 1 behavior according to Treybal’s classification was exhibited by all the mixed extractants [39]. The highly polar nature of the studied DES may be responsible for its immiscibility with *n*-hexane. The biphasic region decreases with increase in % EtOH. Finally, the positive slopes of tie lines show that benzene solubility in *n*-hexane is higher than its solubility in the solvent.

##### Distribution coefficients and selectivities

**Fig. 10:**
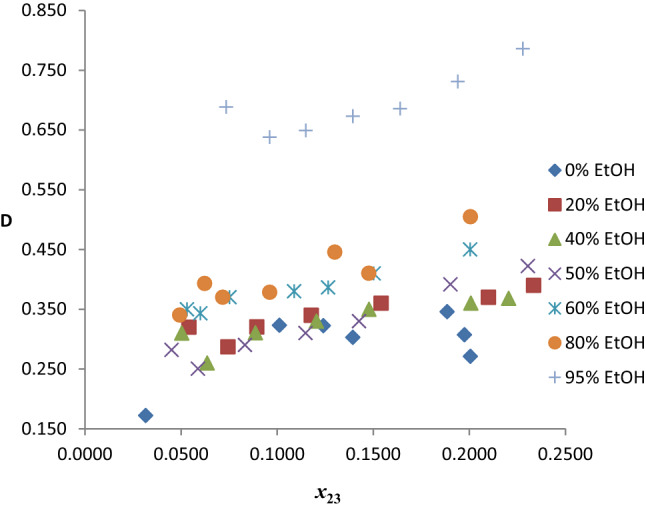
Benzene distribution coefficients as a function of benzene composition in the extract phase for the system *n-*hexane + benzene + reline/EtOH at temperature 303 K and atmospheric pressure

The benzene distribution coefficients (*D*) obtained for the twenty pseudo-ternary systems of *n*-hexane (1) + benzene (2) + mixed solvent (R100–R5Et95) (3) are presented in Table S4. Figure 10 shows a plot of *D* versus benzene composition in the extract phase for the seven chosen systems. The *D* values of the hybrid solvents generally show a gradual increase from % EtOH content of 0–80%, values lie between 0.25 and 0.45, with few outliers. There is a sharp increase in the *D* values for 95% EtOH relative to others. This is largely because of the much higher values of *D* for ethanol compared to neat-reline.

**Fig. 11:**
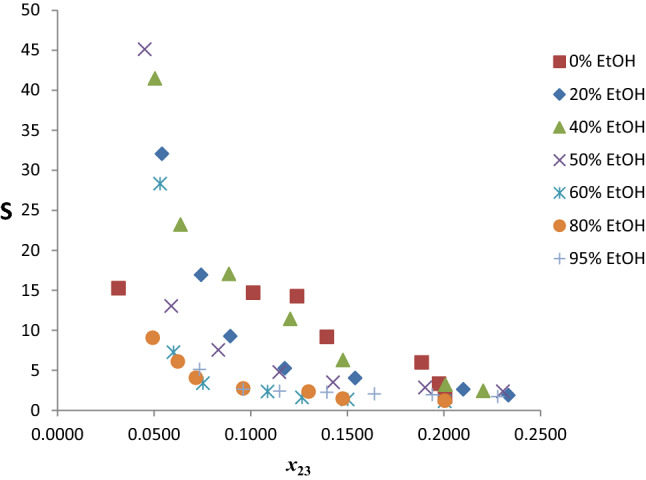
Selectivities as a function of benzene composition in the extract phase for the system *n-*hexane + benzene + reline/EtOH at temperature 303 K and atmospheric pressure

The selectivity values for the twenty pseudo-ternary systems are shown in Table S4 and those of the chosen seven are plotted in Fig. 11. Generally, the *S* values decrease with increasing composition of benzene in the extract phase for the seven extractants. Addition of EtOH significantly improved the selectivity of the hybrid solvents relative to neat-reline up to 60% and thereafter it decreased very sharply. There is a clear enhancement in the *S*_max_ up to 60%, even though the highest value occurs at 50% EtOH. The maximum value of *S* is in the following decreasing order: R50Et50 (50% EtOH, *S* = 45.097) $\succ$ R60Et40 (40% EtOH, *S* = 41.450) $\succ$ R80Et20 (20% EtOH, *S* = 32.042) $\succ$ R40Et60 (60% EtOH, *S* = 28.301) $\succ$ R100 (0% EtOH, *S* = 15.240) $\succ$ R20Et80 (80% EtOH, *S* = 9.071) $\succ$ R5Et95 (95% EtOH, *S* = 5.096). There is a staggering 195.91% increase in the maximum value of *S* at 50% EtOH relative to the neat-reline. The decrease in *S*_max_ from 60 to 80% is profoundly sharp, 67.95% drop, signifying sharp attenuation beyond 60% EtOH. It is pertinent to note that at 10% benzene composition in the extract phase, the *S* value for 0% EtOH (neat-reline) is higher than all hybrid solvents. Also worthy of note is the convergence of *S* values beyond 20% mole fraction of benzene in the extract irrespective of % EtOH. This signifies selectivity becomes insensitive to EtOH addition when the composition of benzene in the extract phase goes above 20%. Similar trend occurred in the extraction of benzene from *n*-hexane using a binary mixed DES (glyceline/reline) as extractant [21]. Generally, selectivity tends to unity as the tie line tends toward the plait point, where the distribution coefficient of solute and other components of the ternary mixture becomes unity. Thus, the observed convergence of *S* values can be attributed to the inability of the hybrid solvents to discriminate beyond 20% mole fraction of benzene as the *S* value has become very low.

### Physicochemical properties of hybrid solvents

The physicochemical properties (density, viscosity and refractive index) of the studied hybrid solvents as measured experimentally are presented and discussed in this section. The three pairs of hybrid solvents are analyzed in the following sub-sections.

#### Ethaline + ethanol

**Fig. 12:**
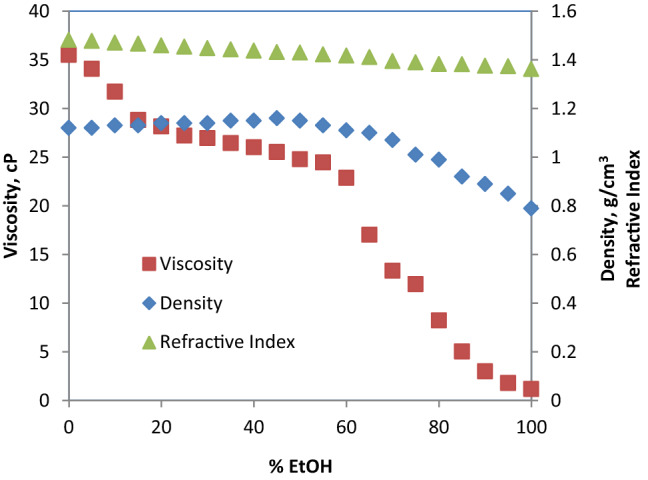
Viscosity, density and refractive index of ethaline/EtOH mixed solvent as a function of volume percent EtOH

Table S5 (supplementary information) shows the density, viscosity and refractive index of the hybrid solvents (ethaline/EtOH) as a function of volume % of EtOH. These values are plotted in Fig. 12. A significant decrease in viscosity of the hybrid solvent is noticed as volume % of EtOH increases. This is evident from the sharp steep in negative slope of viscosity profile in Fig. 12 and it is in agreement with the findings of similar work in this regard [29, 32]. For example, a decrease of 20.7% in viscosity was achieved with 20% EtOH addition while the drop in viscosity at 50% EtOH is 30.17%. The profile for density also shows decrease with increasing volume % EtOH in the hybrid solvent but not as sharp as viscosity. On the other hand, the refractive index shows almost constant values with increasing volume % of EtOH in the mixed solvent.

#### Glyceline + ethanol

**Fig. 13:**
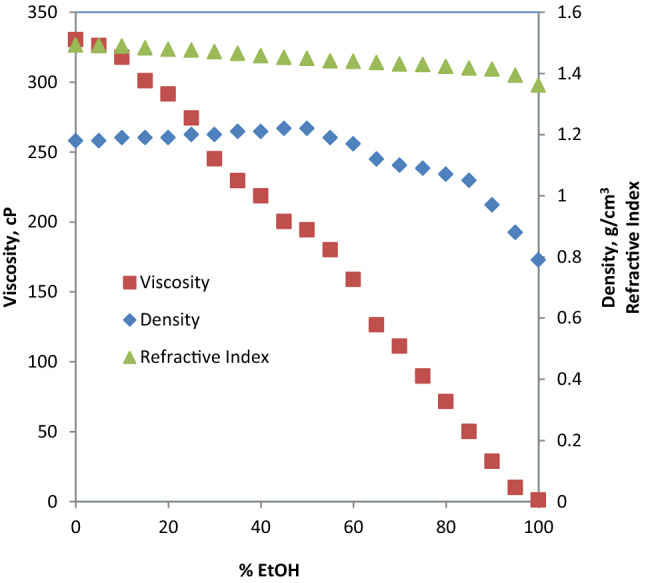
Viscosity, density and refractive index of glyceline/EtOH mixed solvent as a function of volume percent EtOH

Table S6 (supplementary information) shows the density, viscosity and refractive index of the hybrid solvents (glyceline/EtOH) as a function of volume % of EtOH. These values are plotted in Fig. 13. A significant decrease in viscosity of the hybrid solvent is noticed as volume % of EtOH increases. This is evident from the sharp steep in negative slope of viscosity profile in Fig. 13. For example, a decrease of 11.76% in viscosity was achieved with 20% EtOH addition while the drop in viscosity at 50% EtOH is 41.15%. The profile for density also shows decrease with increasing volume % EtOH in the hybrid solvent but not as sharp as viscosity. On the other hand, the refractive index shows almost constant values with increasing volume % of EtOH in the hybrid solvent. Similar findings have been reported in the literature [29, 40].

#### Reline + ethanol

**Fig. 14:**
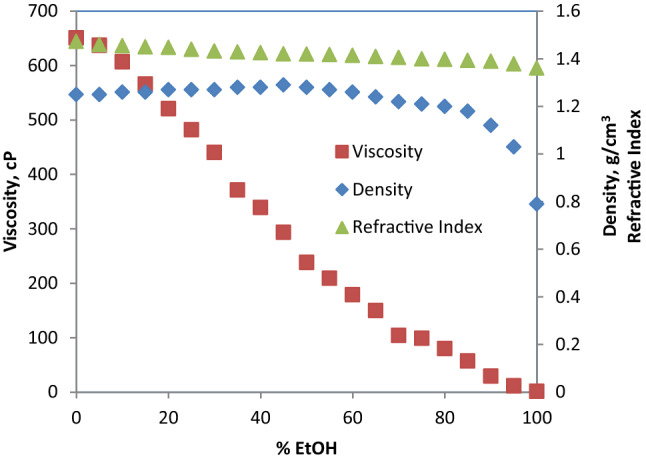
Viscosity, density and refractive index of reline/EtOH mixed solvent as a function of volume percent EtOH

Table S7 (supplementary information) shows the density, viscosity and refractive index of the hybrid solvents (reline/EtOH) as a function of volume % of EtOH. These values are plotted in Fig. 14. A significant decrease in viscosity of the mixed solvent is noticed as volume % of EtOH increases. This is evident from the sharp steep in negative slope of viscosity profile in Fig. 14. For example, a decrease of 19.98% in viscosity was achieved with 20% EtOH addition while the drop in viscosity at 50% EtOH is 63.38%. The profile for density also shows decrease with increasing volume % EtOH in the hybrid solvent but not as sharp as viscosity. On the other hand, the refractive index shows almost constant values with increasing volume % of EtOH in the hybrid solvent. Similar findings have been reported in the literature [31, 41, 42].

### General discussion and comparative analysis

**Fig. 15:**
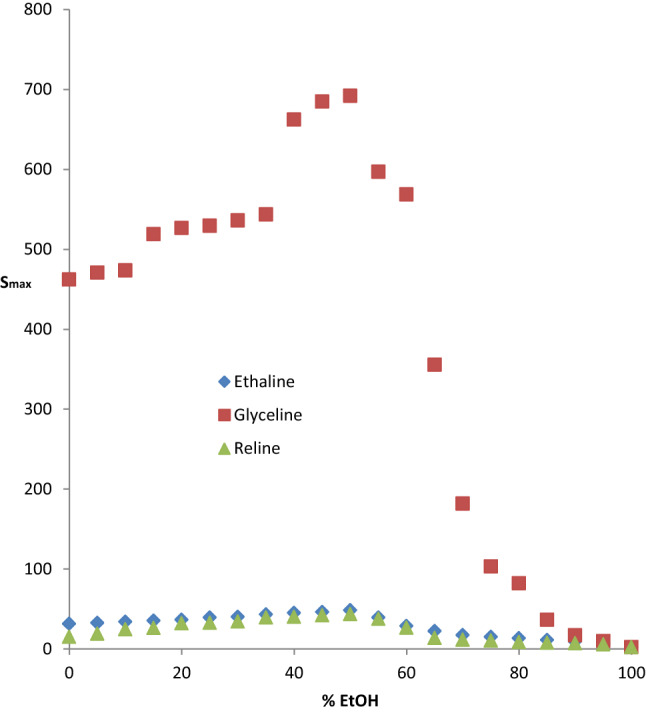
Maximum selectivities of mixed solvents (ethaline/EtOH, glyceline/EtOH, reline/EtOH) as a function of volume percent EtOH

The ternary diagram for the system *n*-hexane + benzene + ethanol provides insight and beneficial guideline for blending gasoline with ethanol in what is now known as gasohol. Such blending must lie within the single-phase region to remain uniform and deliver the requisite fuel function. The two-phase region should be avoided; this is a key contribution of this study. For the hybrid solvents, the expectation is that the properties should lie between those of their constituents. In consonance with this expectation, addition of EtOH imparts viscosity reduction on DES since the viscosity of the former is profoundly lower than that of the latter. By the same reasoning, the selectivities of the hybrid solvents are expected to lie between those of the neat-DES and ethanol. Since the selectivities of neat-DES (ethaline, glyceline and reline) are much higher than that of ethanol, addition of EtOH to these DESs should lower their selectivities. Figure 15 shows the maximum selectivities (*S*_max_) of the hybrid solvents as a function of volume % EtOH. A profound increase in *S*_max_ occurs as % EtOH increases up to 50% and decreases with further increase in EtOH content for all hybrid solvents. This occurrence suggests a strong intermolecular interaction between these DESs and EtOH as % EtOH increases to 50%, which dwindles with further addition of EtOH. The underpinning phenomenological context for this novel performance enhancement should be unraveled by spectroscopic studies. In a related investigation involving two choline chloride-based DESs, Hadj-Kali et al. [43] reported that addition of 50 wt% water breaks the hydrogen bonding between the HBA (choline chloride) and HBD (urea and glycerol). This may well explain the finding of this study. It is also striking to note the remarkably superior performance of glyceline-based solvent relative to the ethaline- and reline-based solvent in this study. This trend was consistently demonstrated in all our previous contributions [19–21]. It is however in sharp contrast to the findings in some related studies in terms of the role of the hydrogen bond donors (glycerol, ethylene glycol, and urea). For example, Naik et al. [44] reported a higher value of selectivity for ethylene glycol-based DES relative to the glycerol-based DES in the extraction of toluene from *n*-heptane. Similar finding was reported by Park [45] in the extraction of toluene from *n*-heptane using a ternary mixed solvent of choline chloride: urea: ethylene glycol/glycerol. The sharp disparity or contrast between their findings and ours may be attributed to the difference in the polarity of the aromatic (toluene/benzene), the hydrogen bond acceptor (choline chloride/methyltriphenylphosphonium bromide), and the hydrogen bond network between HBA and HBD in each case.

The performances of the solvents under study are best benchmarked against the conventional organic solvent commonly used in the industries for separating aromatics from non-aromatics—sulfolane. It is however pertinent to make clarification on the seeming discrepancy between the values of selectivity reported for sulfolane in two different articles for the separation of benzene from *n*-hexane. Shekaari et al. [18] reported *S*_max_ value of 47.7704 at 303.15 K and 0.0865 MPa and another value of 36.3735 at 313.15 K and 0.0865 MPa. This suggests that selectivity is sensitive to both temperature and pressure. On the other hand, Guo et al. [46] reported *S*_max_ value of 42.38 at 303.15 K and 1 atm (0.101325 MPa). Considering the pressure difference, the values reported by the two contributions are in good agreement. Consequently, the comparative analysis is here based on the same temperature (*T* = 303.15 K) and same pressure (*P* = 1 atm) as shown in Table 2. It is instructive to note that both ethaline- and glyceline-based hybrid solvents have benzene distribution coefficient higher than that of sulfolane at 50% EtOH. The selectivities of all hybrid solvents at 50% EtOH are higher than the values for sulfolane. These are remarkable enhancement for both ethaline- and reline-based hybrid solvents whose neat-DES underperforms sulfolane. The best hybrid solvent is glyceline + 50% EtOH (G50Et50), having the highest selectivity value of 692.070, representing 49.73% increase in *S* and with 41.15% reduction in viscosity relative to the neat glyceline.

**Table 2:** Comparison of distribution coefficient, selectivity and viscosity for *n*-hexane + benzene + solvent at 303.15 K and 1 atm

Solvent	*D*	*S*		*η*	References
Sulfolane	0.0171–0.5014	1.87–42.38	10.35^a^		[46]
E50Et50	0.621–0.740	1.036–48.421	24.79		This study
G50Et50	0.710–0.770	1.480–692.070	194.45		This study
R50Et50	0.251–0.422	2.347–45.097	238.33		This study
G80E20	0.651–0.750	1.077–422.485	309.92		[21]
[EMIM][EtSO_4_]	0.0093–0.0265	16.58–67.72	ND		[46]
[EMIM][NTf_2_]	0.0335–0.1331	5.92–40.61	ND		[46]
[EMIM][EtSO_4_]:[EMIM][NTf_2_], 1:9	0.0273–0.0684	11.56–35.91	ND		[46]
[EMIM][EtSO_4_]:[EMIM][NTf_2_], 9:1	0.0114–0.0431	10.58–52.03	ND		[46]

*ND* not determined a—[24] *η*: dynamic viscosity in cP

Table 2 also shows the performance of other solvents, DESs and ionic liquids, used by other workers in the extraction of benzene from *n*-hexane. It is clear that the mixed solvent G50Et50 outperformed the ionic liquids, 1-ethyl-3-methylimidazolium bis(trifluoromethylsulfonyl)imide ([EMIM][NTf_2_]), 1-ethyl-3-methylimidazolium ethylsulfate ([EMIM][EtSO_4_]) and their mixtures, with higher values of *D* and *S*. In our previous contribution, the binary mixed DES, 80% glyceline + 20% ethaline (G80E20), was considered the best in comparison to other mixed DESs evaluated in that study, with a decrease of 8.55% in selectivity and reduction of 9.41% in viscosity relative to neat glyceline [21]. The performance of G50Et50 is superior to G80E20 not only in its higher values of *D* and *S*, but also in much lower viscosity.

Viscosity was observed to generally decrease with increasing proportion of EtOH to the neat DESs (glyceline, ethaline and reline) in the hybrid solvent. The molecular weight of HBD in the hybrid solvent seems to play significant role. The molecular weight of EtOH is 46, which is lower compared to the molecular weights of the primary HBD in the hybrid solvents (glycerol: 92.09; urea: 60.06; and ethylene glycol: 62.07). Thus, increasing content of EtOH, which results in lowering of the average molecular weight of the HBD in the hybrid solvent, causes decrease in viscosity. This is in contradiction to the findings of Al-Dawsari et al. [47], as they observed that the viscosities of DESs increased as the molecular weight of the HBDs increased for the same HBA. This disparity may be attributed to the alteration in the strength and nature of hydrogen bond occasioned by the secondary HBD (EtOH) in the current study.

On the overall basis, using *D*, *S*, and viscosity values, G50Et50 is the best of the mixed solvents under study. Industrial replacement of sulfolane with G50Et50 for liquid–liquid extraction of aromatics from aliphatics will imply lower solvent requirement for the extraction and smaller equipment diameter due to higher *D*, fewer stages and reduced equipment height due to higher *S*, and higher cost of mixing and transport due to higher viscosity. It must be stated that all hybrid solvents with 50% EtOH can conveniently replace sulfolane.

### Consistency of LLE data and thermodynamic modeling

The consistency and reliability of the LLE data were tested using both Othmer-Tobias [48] and Hand [49] correlations as detailed in the supplementary information. As shown in Tables S9 and S10, the coefficient of determination, *R*^2^ > 0.99 for all mixing proportions of DES/EtOH. This clearly validates the consistency and reliability of the experimentally obtained LLE data. Also, thermodynamic modeling of the LLE data was done using both NRTL [50] and UNIQUAC [51] model as detailed in the supplementary information. The binary interaction parameters and root mean square deviation (RSMD) values obtained for both models are presented in Table S12. The highest value of RSMD is 0.0306 for UNIQUAC and 0.0303 for NRTL. Thus, both models adequately describe the experimental LLE data.

## Conclusion

In this study, three categories of hybrid solvents were prepared by mixing choline chloride-based deep eutectic solvents (ethaline, glyceline and reline) with ethanol in various volume proportions to reduce the high viscosity of the neat DESs and enhance their industrial appeal. The hybrid solvents were explored as extractants for the extraction of aromatic (benzene) from aliphatic (*n*-hexane). Results show that addition of ethanol has a novel enhancing influence on the extraction capacity of the DESs up to a point but plummeted same thereafter. The best hybrid solvent was obtained at 50% ethanol content, giving 54.01%, 49.73% and 195.91% increase in the maximum selectivities of ethaline, glyceline and reline, respectively. Thus, ethanol imparts a positive influence on the DESs both in terms of enhanced extraction efficiency and viscosity reduction. The hybrid solvent, glyceline + 50% ethanol (G50Et50), emerged overall best in this regard. Petrochemical industries can therefore embrace this hybrid solvent in place of sulfolane in a drive for green and sustainable paradigm shift. The reliability of the LLE data was confirmed by both Othmer-Tobias and Hand equations. The thermodynamic activity coefficient models of both NRTL and UNIQUAC adequately represent the experimental LLE data.

## Supplementary Information

Below is the link to the electronic supplementary material.

Supplementary file1 (DOCX 2419 kb)
