# Supplementary material for: Evaluation of hybrid solvents featuring choline chloride-based deep eutectic solvents and ethanol as extractants for the liquid–liquid extraction of benzene from n-hexane: towards a green and sustainable paradigm
Source: Appl Petrochem Res. 2021 Sep 25;11(3):335–51. doi: 10.1007/s13203-021-00282-y (PMC8475862; doi:10.1007/s13203-021-00282-y)
Supplement: Supplementary file 1 — Supplementary file1 (DOCX 2419 kb) [file 13203_2021_282_MOESM1_ESM.docx]

Supplementary Information

1. LLE Data for the EtOH

Table S1: LLE data for EtOH

| **Raffinate Phase** | | **Extract Phase** | | **D** | **S** |
| --- | --- | --- | --- | --- | --- |
| ***x*_11_** | ***x*_21_** | ***x*_13_** | ***x*_23_** |  |  |
| 0.8774 | 0.0304 | 0.2744 | 0.0215 | 0.707 | 2.261 |
| 0.8084 | 0.0600 | 0.2757 | 0.0356 | 0.593 | 1.740 |
| 0.7392 | 0.1186 | 0.2761 | 0.0726 | 0.612 | 1.639 |
| 0.6697 | 0.1752 | 0.2763 | 0.1117 | 0.638 | 1.545 |
| 0.5995 | 0.2294 | 0.2765 | 0.1534 | 0.669 | 1.450 |
| 0.5279 | 0.2801 | 0.2776 | 0.1986 | 0.709 | 1.348 |
| 0.4534 | 0.3249 | 0.2814 | 0.2497 | 0.769 | 1.238 |

1. LLE data and ternary diagrams for the ethaline/EtOH hybrid solvent

Table S2: LLE data for ethaline/EtOH hybrid solvent

| **Raffinate Phase** | | **Extract Phase** | | **D** | **S** |
| --- | --- | --- | --- | --- | --- |
| ***x*_11_** | ***x*_21_** | ***x*_13_** | ***x*_23_** |  |  |
| **0% EtOH** | | | | | |
| 0.8710 | 0.1290 | 0.0131 | 0.0610 | 0.473 | 31.440 |
| 0.7901 | 0.2099 | 0.0671 | 0.1001 | 0.477 | 5.615 |
| 0.7310 | 0.2690 | 0.1031 | 0.1410 | 0.524 | 3.716 |
| 0.7189 | 0.2811 | 0.1331 | 0.1635 | 0.582 | 3.142 |
| 0.6551 | 0.3449 | 0.1619 | 0.1869 | 0.542 | 2.193 |
| 0.5579 | 0.4421 | 0.1862 | 0.2154 | 0.487 | 1.460 |
| 0.4338 | 0.5662 | 0.1983 | 0.2987 | 0.528 | 1.154 |
|  |  |  |  |  |  |
| **5% EtOH** | | | | | |
| 0.8795 | 0.1055 | 0.0203 | 0.0793 | 0.752 | 32.566 |
| 0.8187 | 0.1437 | 0.1277 | 0.1063 | 0.740 | 4.743 |
| 0.7511 | 0.2064 | 0.1543 | 0.1589 | 0.770 | 3.748 |
| 0.6830 | 0.2655 | 0.1990 | 0.2205 | 0.831 | 2.850 |
| 0.5928 | 0.3296 | 0.2366 | 0.2658 | 0.806 | 2.021 |
| 0.5109 | 0.3796 | 0.2735 | 0.3227 | 0.850 | 1.588 |
| 0.4575 | 0.4280 | 0.3001 | 0.3510 | 0.820 | 1.250 |
|  |  |  |  |  |  |
| **10% EtOH** | | | | | |
| 0.8777 | 0.1080 | 0.0181 | 0.0756 | 0.700 | 33.944 |
| 0.8151 | 0.1474 | 0.1313 | 0.1076 | 0.730 | 4.532 |
| 0.7486 | 0.2064 | 0.1478 | 0.1548 | 0.750 | 3.799 |
| 0.6813 | 0.2672 | 0.1944 | 0.2031 | 0.760 | 2.664 |
| 0.5775 | 0.3240 | 0.2222 | 0.2301 | 0.710 | 1.846 |
| 0.5096 | 0.3889 | 0.2641 | 0.3033 | 0.780 | 1.505 |
| 0.4555 | 0.4320 | 0.2914 | 0.3586 | 0.830 | 1.298 |
|  |  |  |  |  |  |
| **15% EtOH** | | | | | |
| 0.8750 | 0.1101 | 0.0171 | 0.0759 | 0.689 | 35.275 |
| 0.8133 | 0.1482 | 0.1258 | 0.1082 | 0.730 | 4.720 |
| 0.7462 | 0.2124 | 0.1553 | 0.1529 | 0.720 | 3.459 |
| 0.6801 | 0.2674 | 0.1832 | 0.2006 | 0.750 | 2.785 |
| 0.5758 | 0.3257 | 0.2349 | 0.2475 | 0.760 | 1.863 |
| 0.5072 | 0.3893 | 0.2571 | 0.3037 | 0.780 | 1.539 |
| 0.4528 | 0.4327 | 0.2757 | 0.3418 | 0.790 | 1.297 |
|  |  |  |  |  |  |
| **20% EtOH** | | | | | |
| 0.8732 | 0.1108 | 0.0165 | 0.0765 | 0.690 | 36.539 |
| 0.8116 | 0.1499 | 0.1289 | 0.1094 | 0.730 | 4.595 |
| 0.7444 | 0.2141 | 0.1634 | 0.1606 | 0.750 | 3.417 |
| 0.6787 | 0.2678 | 0.2333 | 0.1955 | 0.730 | 2.124 |
| 0.5741 | 0.3265 | 0.3063 | 0.2482 | 0.760 | 1.425 |
| 0.5050 | 0.3895 | 0.3228 | 0.3077 | 0.790 | 1.236 |
| 0.4503 | 0.4332 | 0.3369 | 0.3422 | 0.790 | 1.056 |
|  |  |  |  |  |  |
| **25% EtOH** | | | | | |
| 0.8715 | 0.1111 | 0.0148 | 0.0744 | 0.670 | 39.433 |
| 0.8102 | 0.1510 | 0.1191 | 0.1088 | 0.721 | 4.902 |
| 0.7432 | 0.2052 | 0.1655 | 0.1416 | 0.690 | 3.099 |
| 0.6766 | 0.2609 | 0.2133 | 0.1931 | 0.740 | 2.348 |
| 0.5718 | 0.3287 | 0.2302 | 0.2498 | 0.760 | 1.888 |
| 0.5034 | 0.3901 | 0.3101 | 0.2926 | 0.750 | 1.218 |
| 0.4487 | 0.4348 | 0.3332 | 0.3435 | 0.790 | 1.064 |
|  |  |  |  |  |  |
| **30% EtOH** | | | | | |
| 0.8696 | 0.1119 | 0.0143 | 0.0739 | 0.660 | 40.160 |
| 0.8087 | 0.1518 | 0.0688 | 0.1047 | 0.690 | 8.107 |
| 0.7417 | 0.2058 | 0.0914 | 0.1502 | 0.730 | 5.923 |
| 0.6749 | 0.2626 | 0.1098 | 0.1865 | 0.710 | 4.365 |
| 0.5701 | 0.3304 | 0.1318 | 0.2511 | 0.760 | 3.287 |
| 0.5016 | 0.3924 | 0.1584 | 0.2904 | 0.740 | 2.344 |
| 0.4480 | 0.4365 | 0.2333 | 0.3448 | 0.790 | 1.517 |
|  |  |  |  |  |  |
| **35% EtOH** | | | | | |
| 0.8688 | 0.1127 | 0.0135 | 0.0755 | 0.670 | 43.113 |
| 0.8071 | 0.1554 | 0.1176 | 0.1057 | 0.680 | 4.668 |
| 0.7397 | 0.2088 | 0.1602 | 0.1504 | 0.720 | 3.326 |
| 0.6732 | 0.2643 | 0.2062 | 0.1877 | 0.710 | 2.319 |
| 0.5684 | 0.3320 | 0.2203 | 0.2457 | 0.740 | 1.909 |
| 0.5001 | 0.3954 | 0.2933 | 0.3005 | 0.760 | 1.296 |
| 0.4468 | 0.4375 | 0.3201 | 0.3458 | 0.790 | 1.103 |
|  |  |  |  |  |  |
| **40% EtOH** | | | | | |
| 0.8672 | 0.1143 | 0.0125 | 0.0743 | 0.650 | 45.097 |
| 0.8064 | 0.1559 | 0.1178 | 0.1060 | 0.680 | 4.654 |
| 0.7381 | 0.2101 | 0.1601 | 0.1513 | 0.720 | 3.320 |
| 0.6720 | 0.2652 | 0.2068 | 0.1856 | 0.700 | 2.274 |
| 0.5668 | 0.3331 | 0.2211 | 0.2432 | 0.730 | 1.872 |
| 0.4994 | 0.3963 | 0.2942 | 0.2918 | 0.736 | 1.250 |
| 0.4454 | 0.4383 | 0.3205 | 0.3375 | 0.770 | 1.070 |
|  |  |  |  |  |  |
| **45% EtOH** | | | | | |
| 0.8667 | 0.1147 | 0.0120 | 0.0734 | 0.640 | 46.219 |
| 0.8049 | 0.1606 | 0.1178 | 0.1076 | 0.670 | 4.578 |
| 0.7368 | 0.2112 | 0.1601 | 0.1501 | 0.711 | 3.271 |
| 0.6706 | 0.2664 | 0.2068 | 0.1838 | 0.690 | 2.237 |
| 0.5655 | 0.3335 | 0.2211 | 0.2436 | 0.730 | 1.868 |
| 0.4978 | 0.3972 | 0.2942 | 0.2939 | 0.740 | 1.252 |
| 0.4445 | 0.4390 | 0.3205 | 0.3336 | 0.760 | 1.054 |
|  |  |  |  |  |  |
| **50% EtOH** | | | | | |
| 0.8658 | 0.1155 | 0.0111 | 0.0717 | 0.621 | 48.421 |
| 0.8031 | 0.1619 | 0.1151 | 0.1069 | 0.660 | 4.607 |
| 0.7354 | 0.2126 | 0.1572 | 0.1488 | 0.700 | 3.274 |
| 0.6693 | 0.2672 | 0.2008 | 0.1817 | 0.680 | 2.267 |
| 0.5640 | 0.3344 | 0.2159 | 0.2441 | 0.730 | 1.907 |
| 0.4962 | 0.3978 | 0.2926 | 0.2944 | 0.740 | 1.255 |
| 0.4432 | 0.4393 | 0.3168 | 0.3252 | 0.740 | 1.036 |
|  |  |  |  |  |  |
| **55% EtOH** | | | | | |
| 0.8646 | 0.1159 | 0.0134 | 0.0707 | 0.610 | 39.359 |
| 0.8018 | 0.1622 | 0.0645 | 0.1038 | 0.640 | 7.955 |
| 0.7342 | 0.2133 | 0.0808 | 0.1408 | 0.660 | 5.998 |
| 0.6678 | 0.2677 | 0.0899 | 0.1713 | 0.640 | 4.753 |
| 0.5623 | 0.3357 | 0.1046 | 0.2276 | 0.678 | 3.645 |
| 0.4949 | 0.3981 | 0.1804 | 0.2747 | 0.690 | 1.893 |
| 0.4421 | 0.4395 | 0.1847 | 0.3077 | 0.700 | 1.676 |
|  |  |  |  |  |  |
| **60% EtOH** | | | | | |
| 0.8638 | 0.1162 | 0.0165 | 0.0640 | 0.551 | 28.834 |
| 0.7997 | 0.1638 | 0.0609 | 0.0966 | 0.590 | 7.744 |
| 0.7325 | 0.2145 | 0.0697 | 0.1223 | 0.570 | 5.992 |
| 0.6664 | 0.2691 | 0.0901 | 0.1588 | 0.590 | 4.365 |
| 0.5609 | 0.3361 | 0.1088 | 0.1983 | 0.590 | 3.042 |
| 0.4936 | 0.3994 | 0.1349 | 0.2437 | 0.610 | 2.233 |
| 0.4401 | 0.4409 | 0.1687 | 0.2934 | 0.665 | 1.736 |
|  |  |  |  |  |  |
| **65% EtOH** | | | | | |
| 0.8622 | 0.1178 | 0.0188 | 0.0577 | 0.490 | 22.464 |
| 0.7978 | 0.1652 | 0.0597 | 0.0859 | 0.520 | 6.949 |
| 0.7305 | 0.2160 | 0.0694 | 0.1123 | 0.520 | 5.473 |
| 0.6651 | 0.2709 | 0.0901 | 0.1409 | 0.520 | 3.839 |
| 0.5587 | 0.3373 | 0.1298 | 0.1822 | 0.540 | 2.325 |
| 0.4915 | 0.4005 | 0.1386 | 0.2203 | 0.550 | 1.951 |
| 0.4388 | 0.4422 | 0.1488 | 0.2521 | 0.570 | 1.681 |
|  |  |  |  |  |  |
| **70% EtOH** | | | | | |
| 0.8613 | 0.1192 | 0.0237 | 0.0572 | 0.480 | 17.439 |
| 0.7961 | 0.1669 | 0.0611 | 0.0851 | 0.510 | 6.644 |
| 0.7282 | 0.2183 | 0.0739 | 0.1092 | 0.500 | 4.929 |
| 0.6634 | 0.2726 | 0.0955 | 0.1445 | 0.530 | 3.682 |
| 0.5566 | 0.3394 | 0.1381 | 0.1833 | 0.540 | 2.177 |
| 0.4897 | 0.4023 | 0.1409 | 0.2132 | 0.530 | 1.842 |
| 0.4365 | 0.4444 | 0.1552 | 0.2398 | 0.540 | 1.518 |
|  |  |  |  |  |  |
| **75% EtOH** | | | | | |
| 0.8601 | 0.1199 | 0.0257 | 0.0540 | 0.450 | 15.073 |
| 0.7949 | 0.1681 | 0.0601 | 0.0791 | 0.471 | 6.224 |
| 0.7267 | 0.2198 | 0.0768 | 0.1012 | 0.460 | 4.357 |
| 0.6613 | 0.2737 | 0.0955 | 0.1341 | 0.490 | 3.393 |
| 0.5552 | 0.3408 | 0.1322 | 0.1705 | 0.500 | 2.101 |
| 0.4880 | 0.4040 | 0.1383 | 0.2101 | 0.520 | 1.835 |
| 0.4351 | 0.4459 | 0.1511 | 0.2319 | 0.520 | 1.498 |
|  |  |  |  |  |  |
| **80% EtOH** | | | | | |
| 0.8585 | 0.1205 | 0.0277 | 0.0518 | 0.430 | 13.323 |
| 0.7931 | 0.1699 | 0.0641 | 0.0782 | 0.460 | 5.695 |
| 0.7252 | 0.2213 | 0.0761 | 0.1018 | 0.460 | 4.384 |
| 0.6594 | 0.2746 | 0.1059 | 0.1318 | 0.480 | 2.989 |
| 0.5540 | 0.3425 | 0.1297 | 0.1678 | 0.490 | 2.093 |
| 0.4862 | 0.4058 | 0.1509 | 0.2029 | 0.500 | 1.611 |
| 0.4326 | 0.4474 | 0.1695 | 0.2237 | 0.500 | 1.276 |
|  |  |  |  |  |  |
| **85% EtOH** | | | | | |
| 0.8570 | 0.1210 | 0.0333 | 0.0533 | 0.440 | 11.336 |
| 0.7916 | 0.1709 | 0.0699 | 0.0820 | 0.480 | 5.434 |
| 0.7229 | 0.2301 | 0.0818 | 0.1082 | 0.470 | 4.156 |
| 0.6575 | 0.2755 | 0.1237 | 0.1295 | 0.470 | 2.498 |
| 0.5519 | 0.3441 | 0.1396 | 0.1686 | 0.490 | 1.937 |
| 0.4847 | 0.4073 | 0.1641 | 0.2037 | 0.500 | 1.477 |
| 0.4303 | 0.4487 | 0.1843 | 0.2289 | 0.510 | 1.191 |
|  |  |  |  |  |  |
| **90% EtOH** | | | | | |
| 0.8556 | 0.1224 | 0.0399 | 0.0526 | 0.430 | 9.215 |
| 0.7892 | 0.1728 | 0.0752 | 0.0812 | 0.470 | 4.932 |
| 0.7205 | 0.2320 | 0.0941 | 0.1044 | 0.450 | 3.446 |
| 0.6550 | 0.2770 | 0.1282 | 0.1331 | 0.481 | 2.455 |
| 0.5494 | 0.3456 | 0.1504 | 0.1763 | 0.510 | 1.863 |
| 0.4823 | 0.4092 | 0.1799 | 0.2046 | 0.500 | 1.340 |
| 0.4287 | 0.4503 | 0.1962 | 0.2297 | 0.510 | 1.115 |
|  |  |  |  |  |  |
| **95% EtOH** | | | | | |
| 0.8520 | 0.1230 | 0.0531 | 0.0529 | 0.430 | 6.901 |
| 0.7881 | 0.1739 | 0.0831 | 0.0783 | 0.450 | 4.270 |
| 0.7189 | 0.2331 | 0.0997 | 0.1026 | 0.440 | 3.174 |
| 0.6518 | 0.2792 | 0.1343 | 0.1284 | 0.460 | 2.232 |
| 0.5469 | 0.3471 | 0.1638 | 0.1666 | 0.480 | 1.603 |
| 0.4796 | 0.4104 | 0.1936 | 0.1929 | 0.470 | 1.164 |
| 0.4265 | 0.4535 | 0.1988 | 0.2222 | 0.490 | 1.051 |


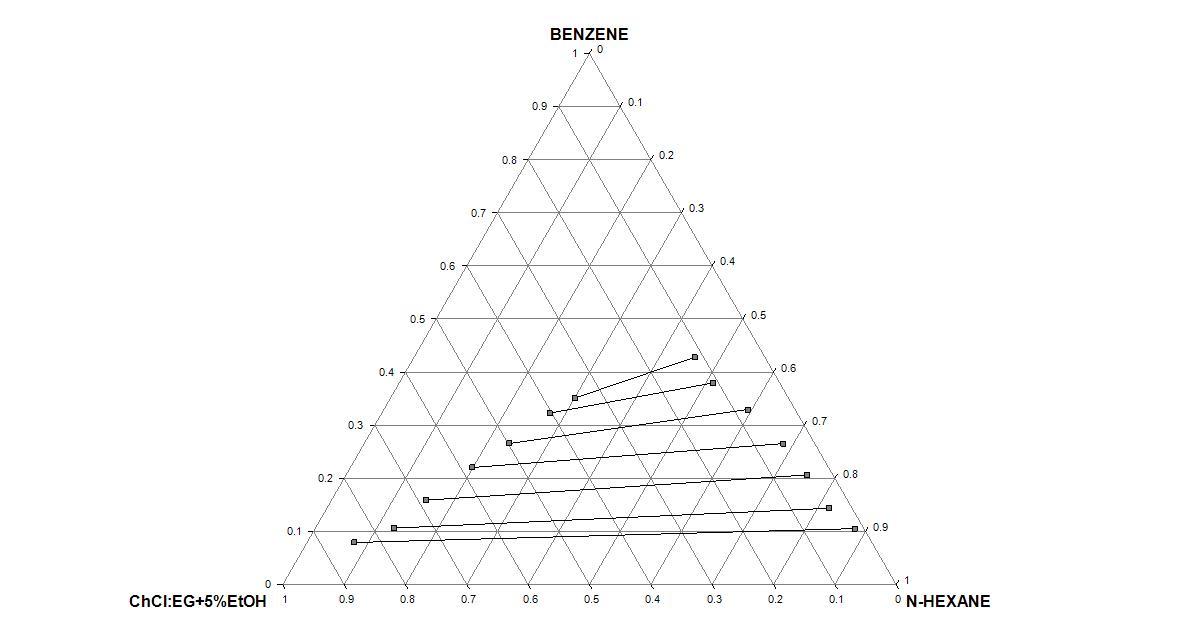


Fig S1a: Experimental tie lines data for the studied mixture of *n-*hexane - benzene –ChCl:EG + 5%EtOH at temperature 303K and atmospheric pressure.


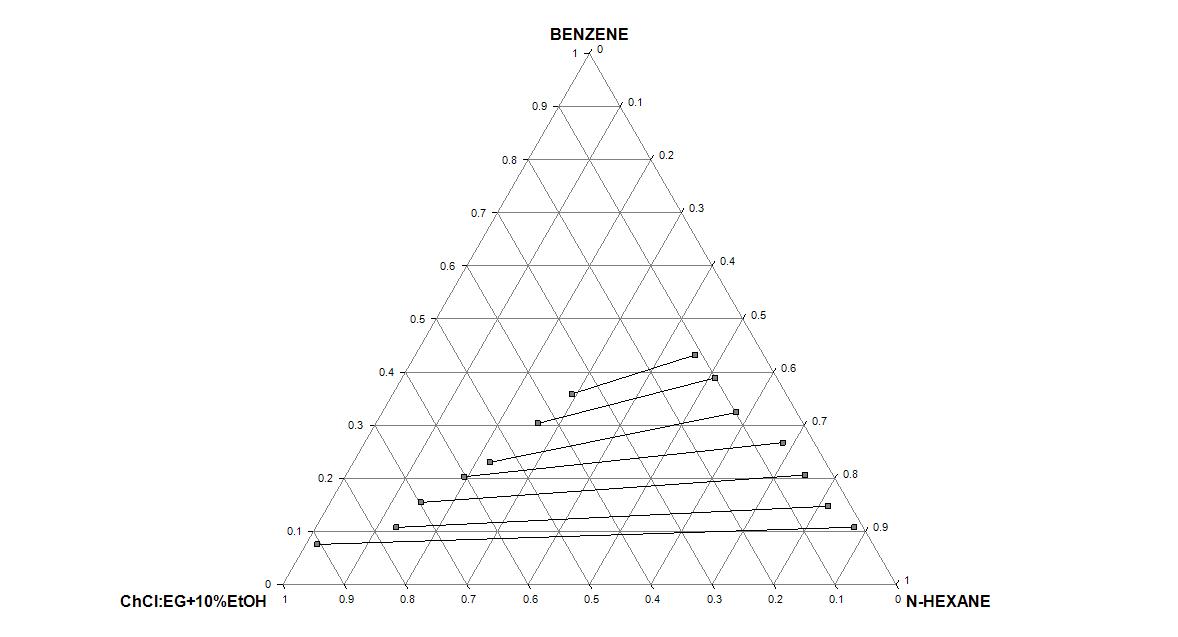


Fig S1b: Experimental tie lines data for the studied mixture of *n-*hexane - benzene –ChCl:EG + 10%EtOH at temperature 303K and atmospheric pressure.


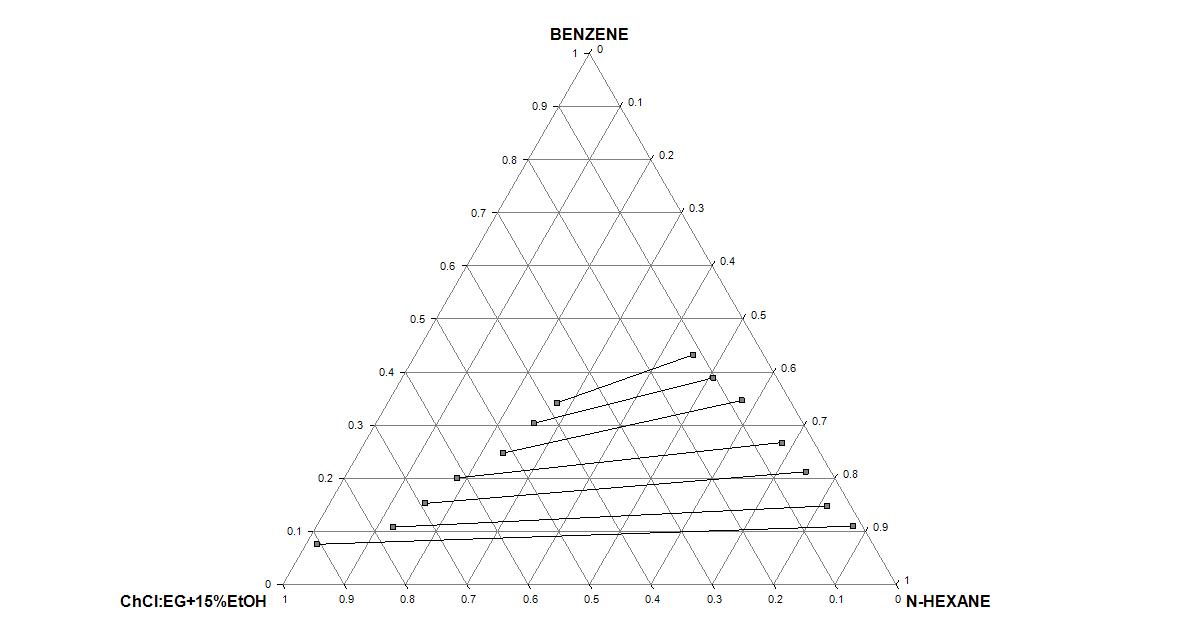


Fig S1c: Experimental tie lines data for the studied mixture of *n-*hexane - benzene –ChCl:EG + 15%EtOH at temperature 303K and atmospheric pressure.


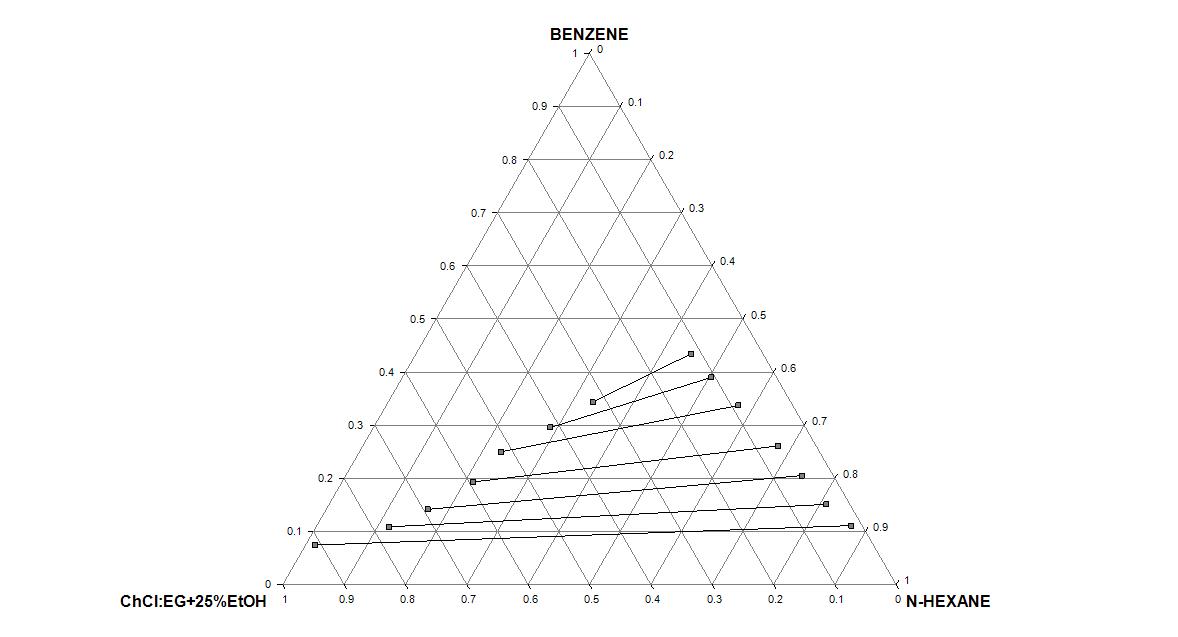


Fig S1d: Experimental tie lines data for the studied mixture of *n-*hexane - benzene –ChCl:EG + 25%EtOH at temperature 303K and atmospheric pressure.


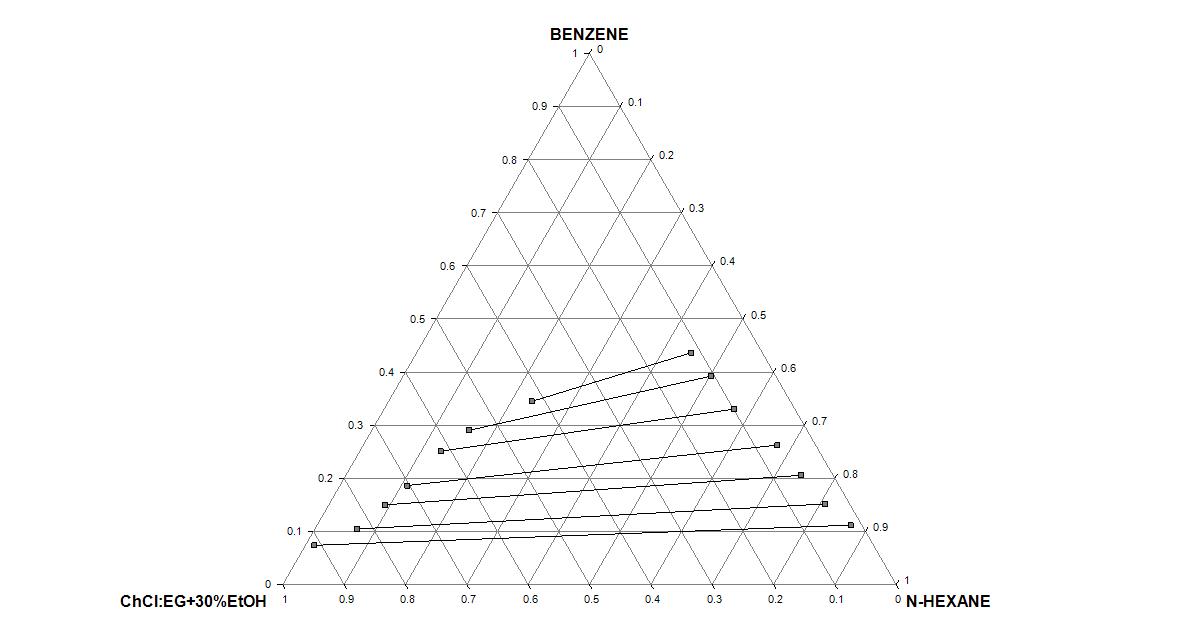


Fig S1e: Experimental tie lines data for the studied mixture of *n-*hexane - benzene –ChCl:EG + 30%EtOH at temperature 303K and atmospheric pressure.


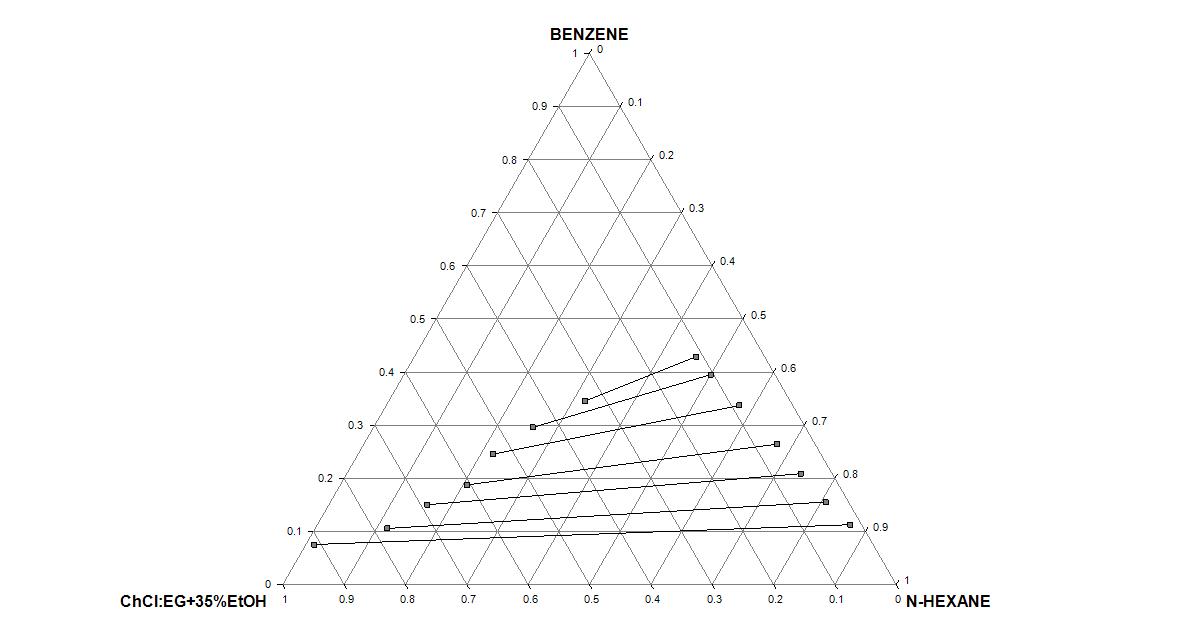


Fig S1f: Experimental tie lines data for the studied mixture of *n-*hexane - benzene –ChCl:EG + 35%EtOH at temperature 303K and atmospheric pressure.


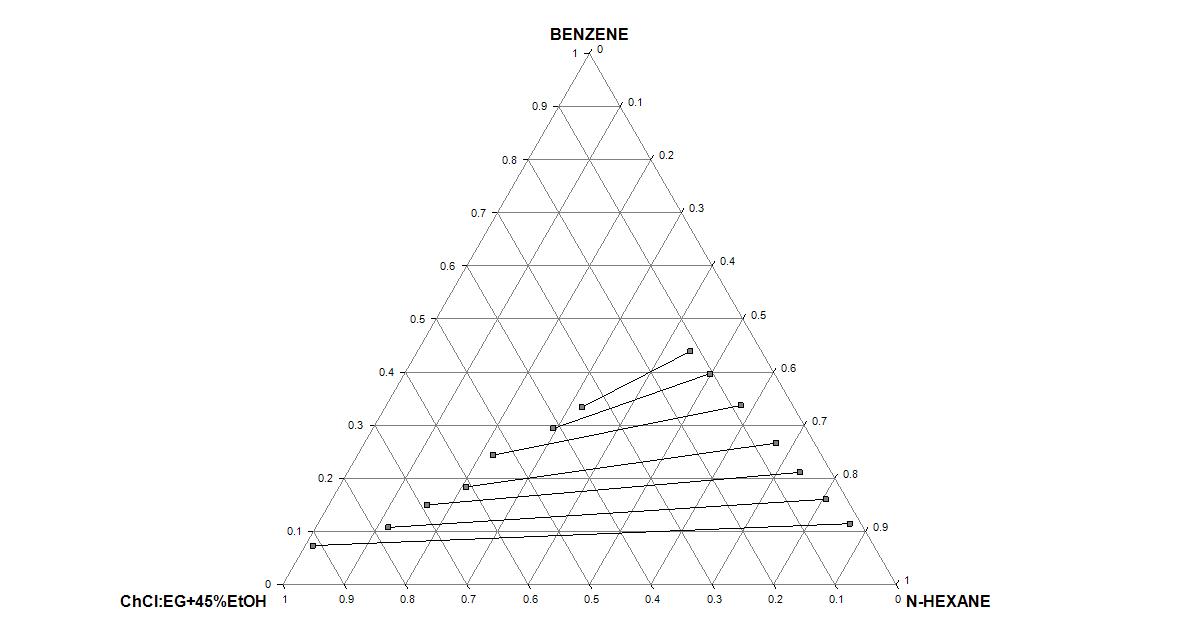


Fig S1g: Experimental tie lines data for the studied mixture of *n-*hexane - benzene –ChCl:EG + 45%EtOH at temperature 303K and atmospheric pressure.


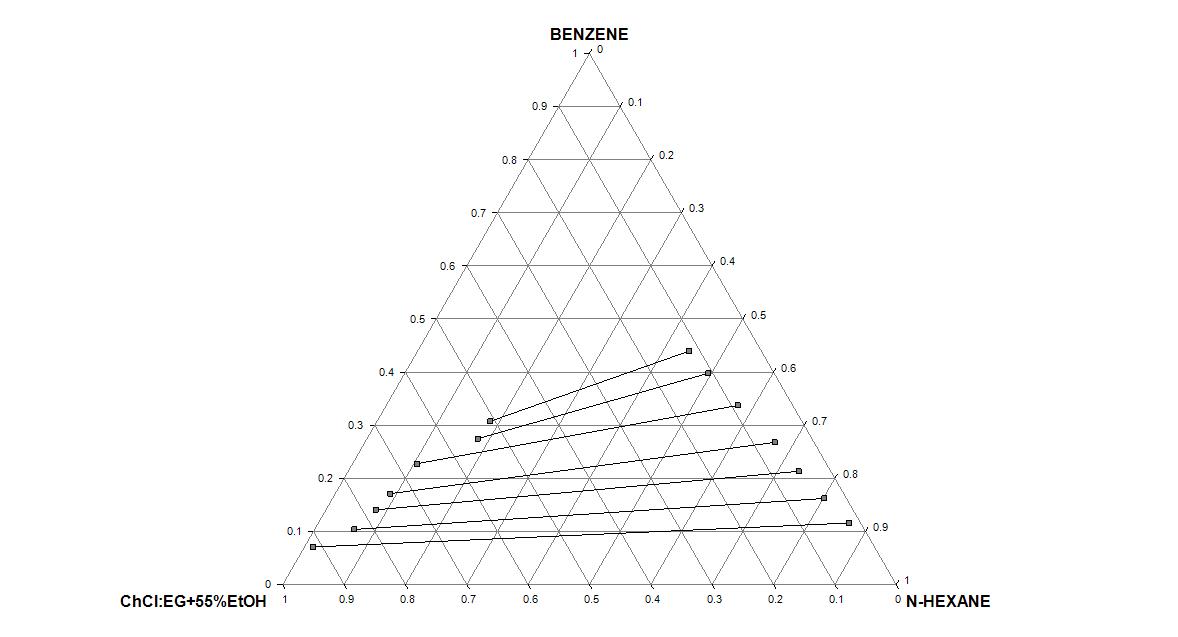


Fig S1h: Experimental tie lines data for the studied mixture of *n-*hexane - benzene –ChCl:EG + 55%EtOH at temperature 303K and atmospheric pressure.


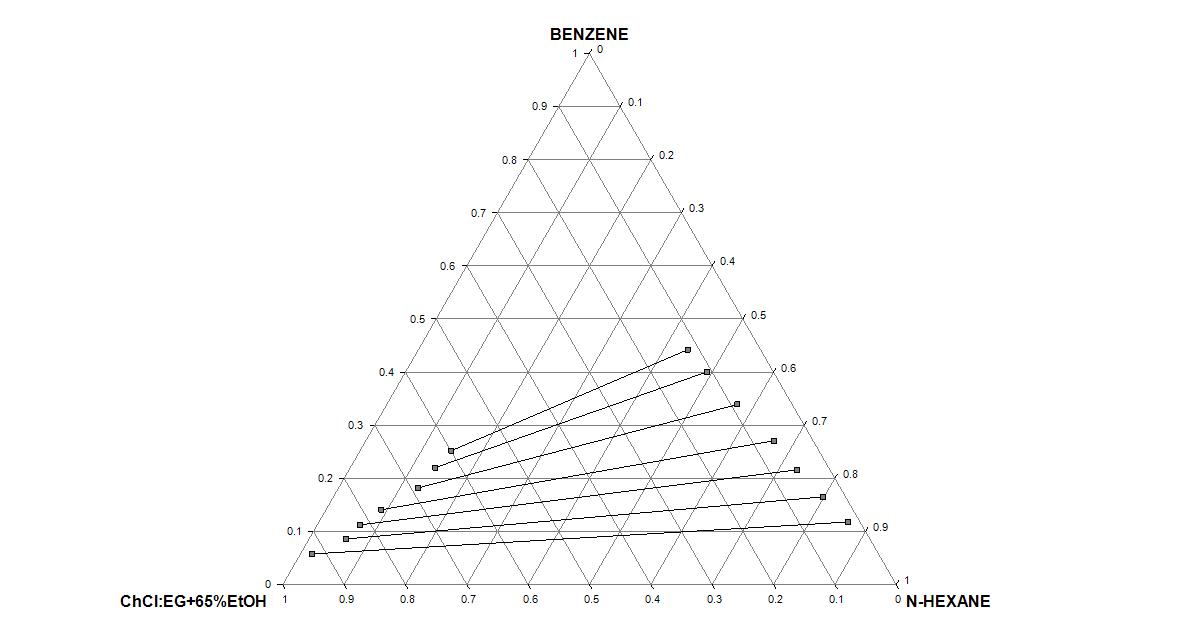


Fig S1i: Experimental tie lines data for the studied mixture of *n-*hexane - benzene –ChCl:EG + 65%EtOH at temperature 303K and atmospheric pressure.


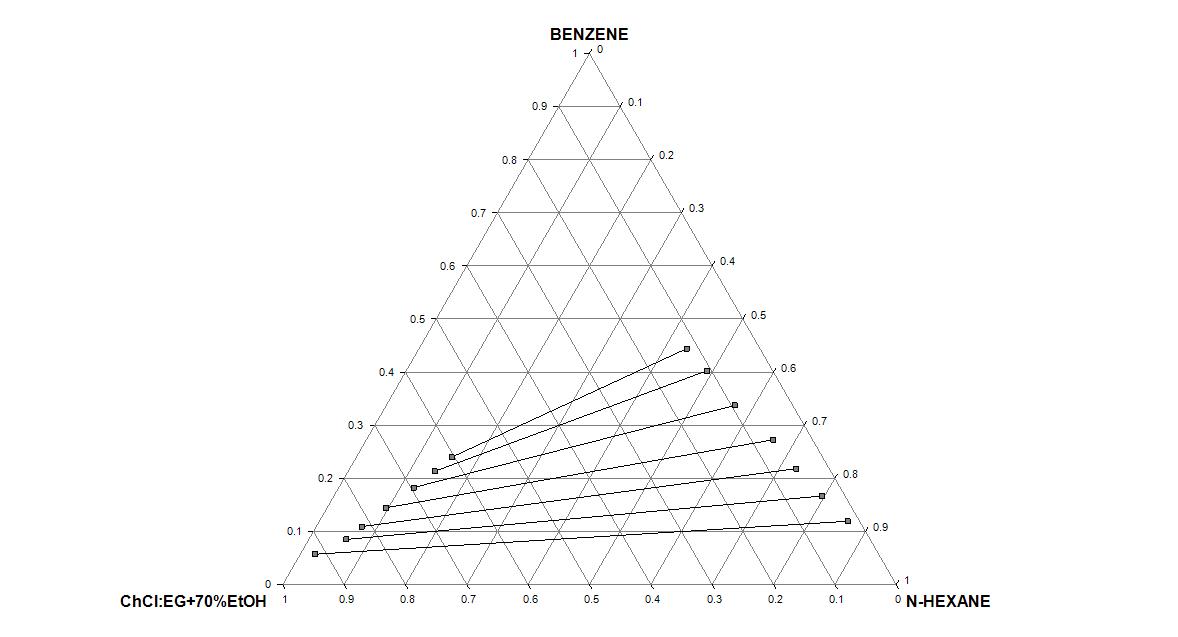


Fig S1j: Experimental tie lines data for the studied mixture of *n-*hexane - benzene –ChCl:EG + 70%EtOH at temperature 303K and atmospheric pressure.


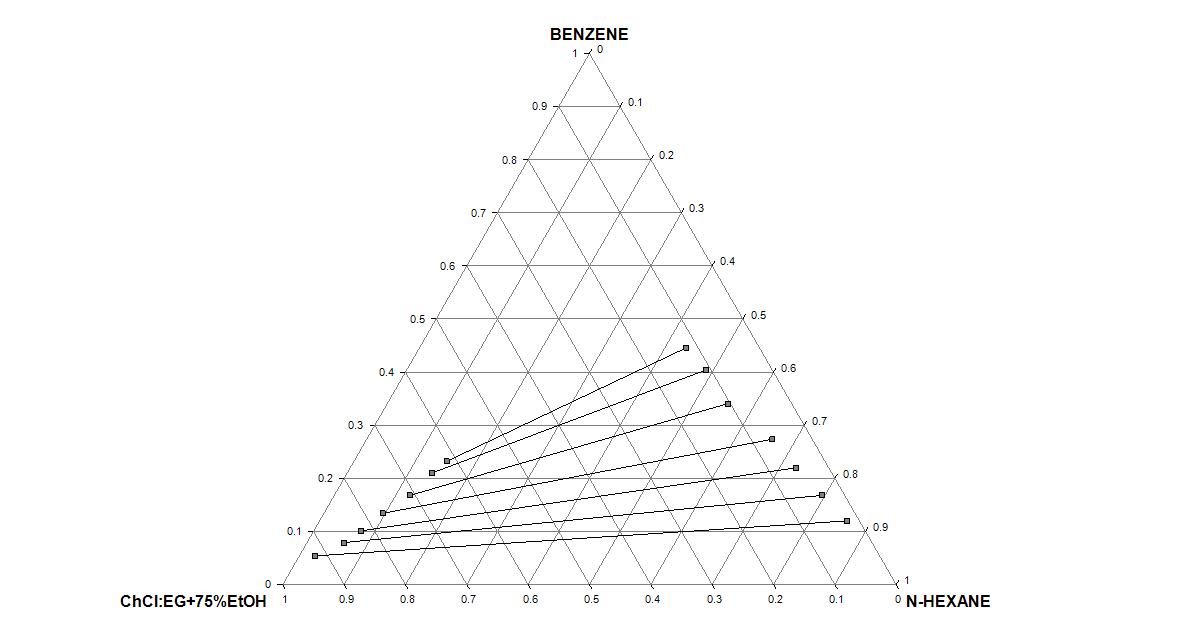


Fig S1k: Experimental tie lines data for the studied mixture of *n-*hexane - benzene –ChCl:EG + 75%EtOH at temperature 303K and atmospheric pressure.


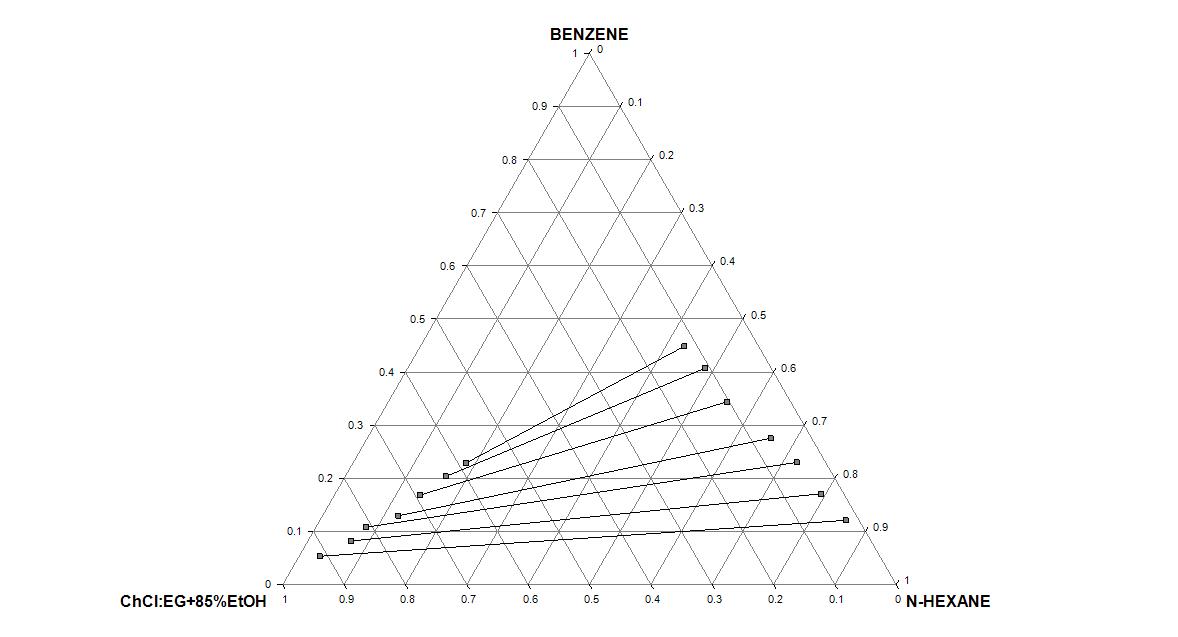


Fig S1l: Experimental tie lines data for the studied mixture of *n-*hexane - benzene –ChCl:EG + 85%EtOH at temperature 303K and atmospheric pressure.


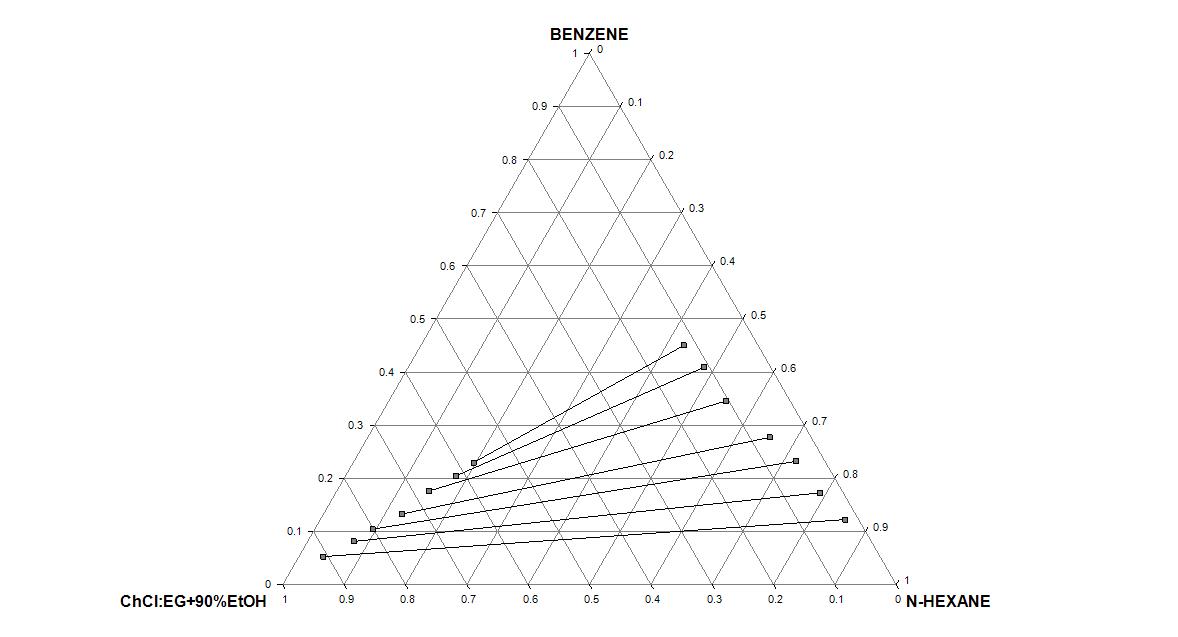


Fig S1m: Experimental tie lines data for the studied mixture of *n-*hexane - benzene –ChCl:EG + 90%EtOH at temperature 303K and atmospheric pressure.

1. LLE data and ternary diagrams for the glyceline/EtOH hybrid solvent

Table S3: LLE data for the glyceline/EtOH hybridd solvent

| **Raffinate Phase** | | **Extract Phase** | | **D** | **S** |
| --- | --- | --- | --- | --- | --- |
| ***x*_11_** | ***x*_21_** | ***x*_13_** | ***x*_23_** |  |  |
| **0% EtOH** | | | | | |
| 0.8489 | 0.1511 | 0.0011 | 0.0905 | 0.599 | 462.219 |
| 0.7608 | 0.2392 | 0.0045 | 0.2116 | 0.885 | 149.559 |
| 0.6600 | 0.3400 | 0.1213 | 0.3086 | 0.908 | 4.939 |
| 0.5372 | 0.4628 | 0.1413 | 0.3996 | 0.863 | 3.283 |
| 0.3266 | 0.6734 | 0.1966 | 0.4864 | 0.722 | 1.200 |
| 0.3033 | 0.6967 | 0.2289 | 0.5291 | 0.759 | 1.006 |
| 0.2912 | 0.7088 | 0.2517 | 0.5903 | 0.833 | 0.964 |
|  |  |  |  |  |  |
| **5% EtOH** | | | | | |
| 0.8582 | 0.1143 | 0.0014 | 0.0878 | 0.768 | 470.878 |
| 0.7849 | 0.1851 | 0.0043 | 0.1629 | 0.880 | 160.643 |
| 0.7011 | 0.2644 | 0.0477 | 0.2380 | 0.900 | 13.231 |
| 0.6330 | 0.3285 | 0.0604 | 0.2661 | 0.810 | 8.489 |
| 0.5672 | 0.3928 | 0.0949 | 0.3339 | 0.850 | 5.081 |
| 0.5007 | 0.4568 | 0.1308 | 0.3974 | 0.870 | 3.330 |
| 0.4665 | 0.4860 | 0.1931 | 0.4325 | 0.890 | 2.150 |
|  |  |  |  |  |  |
| **10% EtOH** | | | | | |
| 0.8549 | 0.1226 | 0.0014 | 0.0951 | 0.776 | 473.672 |
| 0.7801 | 0.1924 | 0.0045 | 0.1712 | 0.890 | 154.254 |
| 0.6975 | 0.269 | 0.0203 | 0.2448 | 0.910 | 31.269 |
| 0.6222 | 0.3393 | 0.0513 | 0.2647 | 0.780 | 9.462 |
| 0.5887 | 0.3663 | 0.1018 | 0.304 | 0.830 | 4.799 |
| 0.5364 | 0.4131 | 0.1466 | 0.3511 | 0.850 | 3.110 |
| 0.4828 | 0.4622 | 0.2135 | 0.4067 | 0.880 | 1.990 |
|  |  |  |  |  |  |
| **15% EtOH** | | | | | |
| 0.8520 | 0.1230 | 0.0012 | 0.0899 | 0.731 | 518.935 |
| 0.7783 | 0.1932 | 0.0038 | 0.1700 | 0.880 | 180.221 |
| 0.6942 | 0.2723 | 0.0173 | 0.2423 | 0.890 | 35.706 |
| 0.6189 | 0.3411 | 0.0422 | 0.2661 | 0.780 | 11.441 |
| 0.5664 | 0.3891 | 0.0671 | 0.2802 | 0.720 | 6.079 |
| 0.5190 | 0.4285 | 0.1241 | 0.3257 | 0.760 | 3.179 |
| 0.4776 | 0.4649 | 0.2097 | 0.3980 | 0.856 | 1.950 |
|  |  |  |  |  |  |
| **20% EtOH** | | | | | |
| 0.8502 | 0.1248 | 0.0012 | 0.0928 | 0.744 | 526.833 |
| 0.7758 | 0.1947 | 0.0033 | 0.1538 | 0.790 | 185.706 |
| 0.6903 | 0.2752 | 0.0161 | 0.2284 | 0.830 | 35.584 |
| 0.6144 | 0.3456 | 0.0433 | 0.2938 | 0.850 | 12.063 |
| 0.5631 | 0.3924 | 0.0795 | 0.3178 | 0.810 | 5.736 |
| 0.5147 | 0.4338 | 0.1434 | 0.3601 | 0.830 | 2.979 |
| 0.4740 | 0.4675 | 0.2110 | 0.3974 | 0.850 | 1.910 |
|  |  |  |  |  |  |
| **25% EtOH** | | | | | |
| 0.8481 | 0.1265 | 0.0011 | 0.0869 | 0.687 | 529.643 |
| 0.7720 | 0.1985 | 0.0068 | 0.1451 | 0.731 | 82.988 |
| 0.6889 | 0.2776 | 0.0197 | 0.2082 | 0.750 | 26.227 |
| 0.6118 | 0.3527 | 0.0353 | 0.2738 | 0.776 | 13.454 |
| 0.5615 | 0.3985 | 0.0583 | 0.3029 | 0.760 | 7.321 |
| 0.5124 | 0.4441 | 0.1639 | 0.3508 | 0.790 | 2.469 |
| 0.4716 | 0.4759 | 0.2435 | 0.3807 | 0.800 | 1.549 |
|  |  |  |  |  |  |
| **30% EtOH** | | | | | |
| 0.8465 | 0.1279 | 0.0011 | 0.0891 | 0.697 | 536.095 |
| 0.7705 | 0.1980 | 0.0080 | 0.1465 | 0.740 | 71.262 |
| 0.6873 | 0.2752 | 0.0261 | 0.2092 | 0.760 | 20.018 |
| 0.6101 | 0.3489 | 0.0811 | 0.2721 | 0.780 | 5.867 |
| 0.5594 | 0.3956 | 0.1672 | 0.2967 | 0.750 | 2.509 |
| 0.5107 | 0.4388 | 0.2081 | 0.3379 | 0.770 | 1.890 |
| 0.4702 | 0.4768 | 0.2382 | 0.3767 | 0.790 | 1.560 |
|  |  |  |  |  |  |
| **35% EtOH** | | | | | |
| 0.8456 | 0.1291 | 0.0011 | 0.0913 | 0.707 | 543.647 |
| 0.7684 | 0.2011 | 0.0073 | 0.1448 | 0.720 | 75.792 |
| 0.6859 | 0.2791 | 0.0229 | 0.2121 | 0.760 | 22.762 |
| 0.6073 | 0.3542 | 0.0951 | 0.2727 | 0.770 | 4.917 |
| 0.5568 | 0.4017 | 0.1699 | 0.3013 | 0.750 | 2.458 |
| 0.5082 | 0.4433 | 0.2236 | 0.3414 | 0.770 | 1.750 |
| 0.4677 | 0.4788 | 0.2354 | 0.3735 | 0.780 | 1.550 |
|  |  |  |  |  |  |
| **40% EtOH** | | | | | |
| 0.8442 | 0.1307 | 0.0009 | 0.0923 | 0.706 | 662.413 |
| 0.7665 | 0.2022 | 0.0076 | 0.1477 | 0.730 | 73.671 |
| 0.6838 | 0.2772 | 0.0244 | 0.2107 | 0.760 | 21.302 |
| 0.6040 | 0.3535 | 0.0456 | 0.2687 | 0.760 | 10.068 |
| 0.5534 | 0.4016 | 0.1001 | 0.3092 | 0.770 | 4.256 |
| 0.5055 | 0.4440 | 0.1938 | 0.3286 | 0.740 | 1.930 |
| 0.4641 | 0.4824 | 0.2279 | 0.3717 | 0.771 | 1.569 |
|  |  |  |  |  |  |
| **45% EtOH** | | | | | |
| 0.8421 | 0.1309 | 0.0009 | 0.0958 | 0.732 | 684.774 |
| 0.7614 | 0.2091 | 0.0071 | 0.1464 | 0.700 | 75.083 |
| 0.6791 | 0.2869 | 0.0207 | 0.2037 | 0.710 | 23.293 |
| 0.5975 | 0.3640 | 0.0361 | 0.2585 | 0.710 | 11.754 |
| 0.5480 | 0.4070 | 0.0914 | 0.3012 | 0.740 | 4.437 |
| 0.4994 | 0.4491 | 0.2015 | 0.3279 | 0.730 | 1.810 |
| 0.4575 | 0.4850 | 0.2255 | 0.3638 | 0.750 | 1.522 |
|  |  |  |  |  |  |
| **50% EtOH** | | | | | |
| 0.8391 | 0.1331 | 0.0009 | 0.0988 | 0.742 | 692.070 |
| 0.7582 | 0.2073 | 0.0071 | 0.1472 | 0.710 | 75.829 |
| 0.6743 | 0.2852 | 0.0192 | 0.2025 | 0.710 | 24.936 |
| 0.5943 | 0.3589 | 0.0365 | 0.2656 | 0.740 | 12.049 |
| 0.5393 | 0.4087 | 0.0861 | 0.2984 | 0.730 | 4.573 |
| 0.4922 | 0.4503 | 0.2063 | 0.3377 | 0.750 | 1.789 |
| 0.4540 | 0.4860 | 0.2362 | 0.3742 | 0.770 | 1.480 |
|  |  |  |  |  |  |
| **55% EtOH** | | | | | |
| 0.8372 | 0.1363 | 0.0010 | 0.0972 | 0.713 | 597.035 |
| 0.7575 | 0.2120 | 0.0064 | 0.1421 | 0.670 | 79.334 |
| 0.6718 | 0.2887 | 0.0193 | 0.1993 | 0.690 | 24.029 |
| 0.5900 | 0.3675 | 0.0352 | 0.2499 | 0.680 | 11.398 |
| 0.5345 | 0.4160 | 0.0863 | 0.2912 | 0.700 | 4.335 |
| 0.4872 | 0.4563 | 0.2005 | 0.3286 | 0.720 | 1.750 |
| 0.4494 | 0.4881 | 0.2359 | 0.3612 | 0.740 | 1.410 |
|  |  |  |  |  |  |
| **60% EtOH** | | | | | |
| 0.8351 | 0.1364 | 0.0010 | 0.0929 | 0.681 | 568.774 |
| 0.7550 | 0.2130 | 0.0072 | 0.1448 | 0.680 | 71.286 |
| 0.6700 | 0.2925 | 0.0212 | 0.2048 | 0.700 | 22.128 |
| 0.5875 | 0.3710 | 0.0386 | 0.2559 | 0.690 | 10.498 |
| 0.5308 | 0.4207 | 0.0938 | 0.3113 | 0.740 | 4.187 |
| 0.4848 | 0.4627 | 0.2043 | 0.3332 | 0.720 | 1.709 |
| 0.4462 | 0.4943 | 0.2394 | 0.3658 | 0.740 | 1.379 |
|  |  |  |  |  |  |
| **65% EtOH** | | | | | |
| 0.8340 | 0.1418 | 0.0015 | 0.0907 | 0.640 | 355.636 |
| 0.7532 | 0.2143 | 0.0096 | 0.1457 | 0.680 | 53.343 |
| 0.6671 | 0.2944 | 0.0106 | 0.1973 | 0.670 | 42.177 |
| 0.5838 | 0.3747 | 0.0168 | 0.2548 | 0.680 | 23.630 |
| 0.5265 | 0.4240 | 0.0891 | 0.2968 | 0.700 | 4.136 |
| 0.4803 | 0.4662 | 0.1992 | 0.3263 | 0.700 | 1.688 |
| 0.4433 | 0.4982 | 0.2316 | 0.3537 | 0.710 | 1.359 |
|  |  |  |  |  |  |
| **70% EtOH** | | | | | |
| 0.8333 | 0.1428 | 0.0027 | 0.0841 | 0.589 | 181.763 |
| 0.7507 | 0.2178 | 0.0118 | 0.1372 | 0.630 | 40.076 |
| 0.6656 | 0.2959 | 0.0155 | 0.1835 | 0.620 | 26.630 |
| 0.5801 | 0.3774 | 0.0204 | 0.2415 | 0.640 | 18.197 |
| 0.5235 | 0.4285 | 0.0855 | 0.2785 | 0.650 | 3.979 |
| 0.4770 | 0.4700 | 0.1879 | 0.3055 | 0.650 | 1.650 |
| 0.4392 | 0.4998 | 0.2195 | 0.3371 | 0.674 | 1.350 |
|  |  |  |  |  |  |
| **75% EtOH** | | | | | |
| 0.8305 | 0.1440 | 0.0046 | 0.0824 | 0.572 | 103.311 |
| 0.7449 | 0.2236 | 0.0137 | 0.1364 | 0.610 | 33.168 |
| 0.6614 | 0.2991 | 0.0158 | 0.1765 | 0.590 | 24.702 |
| 0.5768 | 0.3807 | 0.0176 | 0.2360 | 0.620 | 20.316 |
| 0.5188 | 0.4317 | 0.0834 | 0.2719 | 0.630 | 3.918 |
| 0.4745 | 0.4725 | 0.1734 | 0.2882 | 0.610 | 1.669 |
| 0.4360 | 0.4995 | 0.2081 | 0.3147 | 0.630 | 1.320 |
|  |  |  |  |  |  |
| **80% EtOH** | | | | | |
| 0.8285 | 0.1452 | 0.0055 | 0.0792 | 0.545 | 82.165 |
| 0.7421 | 0.2264 | 0.0141 | 0.1335 | 0.590 | 31.035 |
| 0.6555 | 0.3060 | 0.0150 | 0.1714 | 0.560 | 24.478 |
| 0.5717 | 0.3868 | 0.0164 | 0.2205 | 0.570 | 19.872 |
| 0.5130 | 0.4375 | 0.0795 | 0.2581 | 0.590 | 3.807 |
| 0.4705 | 0.4765 | 0.1706 | 0.2764 | 0.580 | 1.600 |
| 0.4323 | 0.4992 | 0.2027 | 0.2995 | 0.600 | 1.280 |
|  |  |  |  |  |  |
| **85% EtOH** | | | | | |
| 0.8260 | 0.1465 | 0.0118 | 0.0762 | 0.520 | 36.410 |
| 0.7395 | 0.2280 | 0.0177 | 0.1254 | 0.550 | 22.979 |
| 0.6522 | 0.3083 | 0.0178 | 0.1634 | 0.530 | 19.420 |
| 0.5684 | 0.3881 | 0.0189 | 0.2135 | 0.550 | 16.544 |
| 0.5076 | 0.4439 | 0.0838 | 0.2441 | 0.550 | 3.331 |
| 0.4661 | 0.4814 | 0.1623 | 0.2599 | 0.540 | 1.550 |
| 0.4283 | 0.4987 | 0.1855 | 0.2743 | 0.550 | 1.270 |
|  |  |  |  |  |  |
| **90% EtOH** | | | | | |
| 0.8238 | 0.1487 | 0.0242 | 0.0744 | 0.500 | 17.032 |
| 0.7351 | 0.2334 | 0.0361 | 0.1237 | 0.530 | 10.792 |
| 0.6474 | 0.3081 | 0.0422 | 0.1602 | 0.520 | 7.977 |
| 0.5633 | 0.3852 | 0.0595 | 0.2041 | 0.530 | 5.016 |
| 0.5020 | 0.4380 | 0.0900 | 0.2321 | 0.530 | 2.956 |
| 0.4606 | 0.4689 | 0.1680 | 0.2532 | 0.540 | 1.480 |
| 0.4235 | 0.4970 | 0.1875 | 0.2684 | 0.540 | 1.220 |
|  |  |  |  |  |  |
| **95% EtOH** | | | | | |
| 0.8205 | 0.1520 | 0.0369 | 0.0669 | 0.440 | 9.787 |
| 0.7318 | 0.2367 | 0.0504 | 0.1088 | 0.460 | 6.674 |
| 0.6440 | 0.3075 | 0.0581 | 0.1384 | 0.450 | 4.989 |
| 0.5582 | 0.3833 | 0.0613 | 0.1801 | 0.470 | 4.279 |
| 0.4951 | 0.4364 | 0.1103 | 0.1964 | 0.450 | 2.020 |
| 0.4540 | 0.4665 | 0.1547 | 0.2145 | 0.460 | 1.349 |
| 0.4151 | 0.4934 | 0.1711 | 0.2318 | 0.470 | 1.140 |


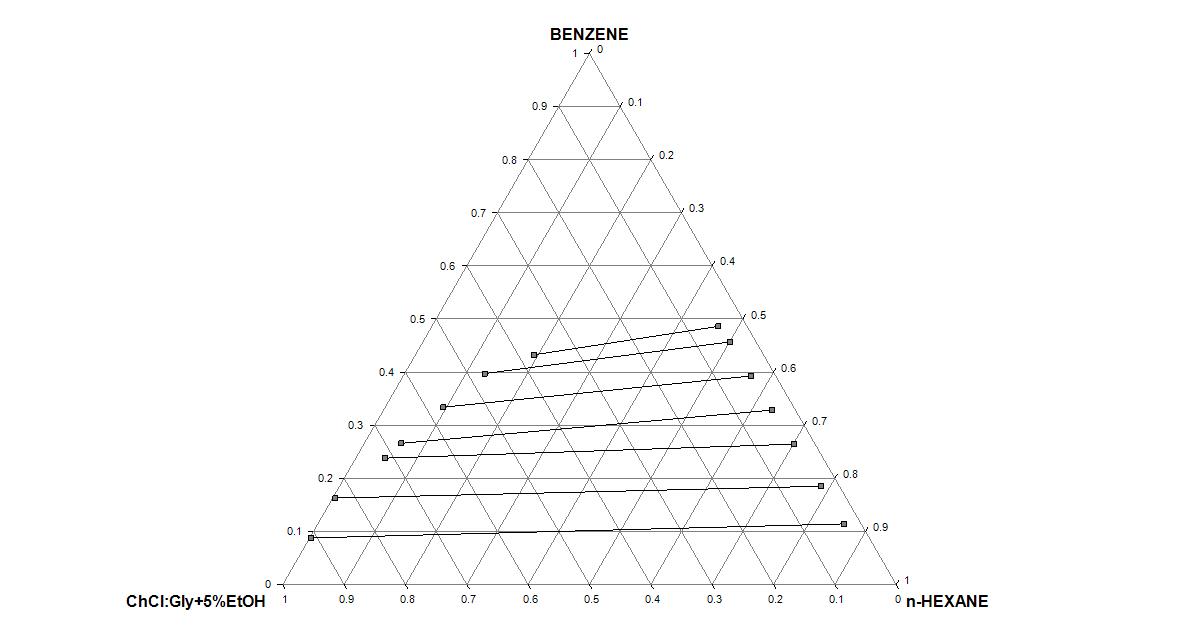


Fig S2a: Experimental tie lines data for the studied mixture of *n-*hexane - benzene – ChCl:Gly + 5%EtOH at temperature 303K and atmospheric pressure.


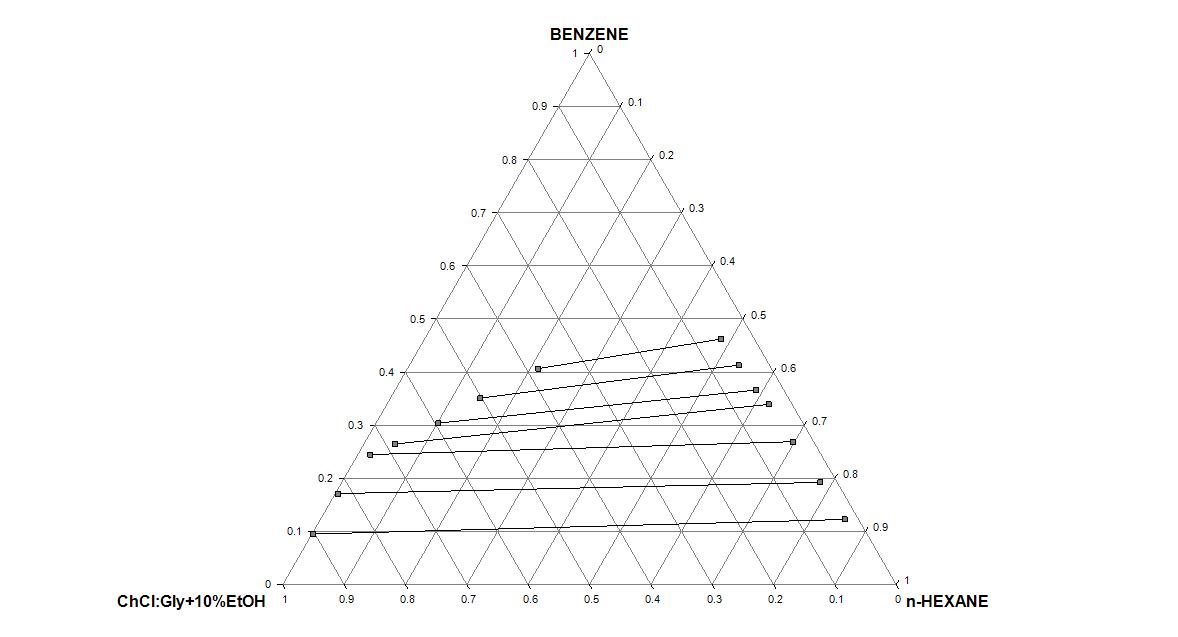


Fig S2b: Experimental tie lines data for the studied mixture of *n-*hexane - benzene – ChCl:Gly + 10%EtOH at temperature 303K and atmospheric pressure.


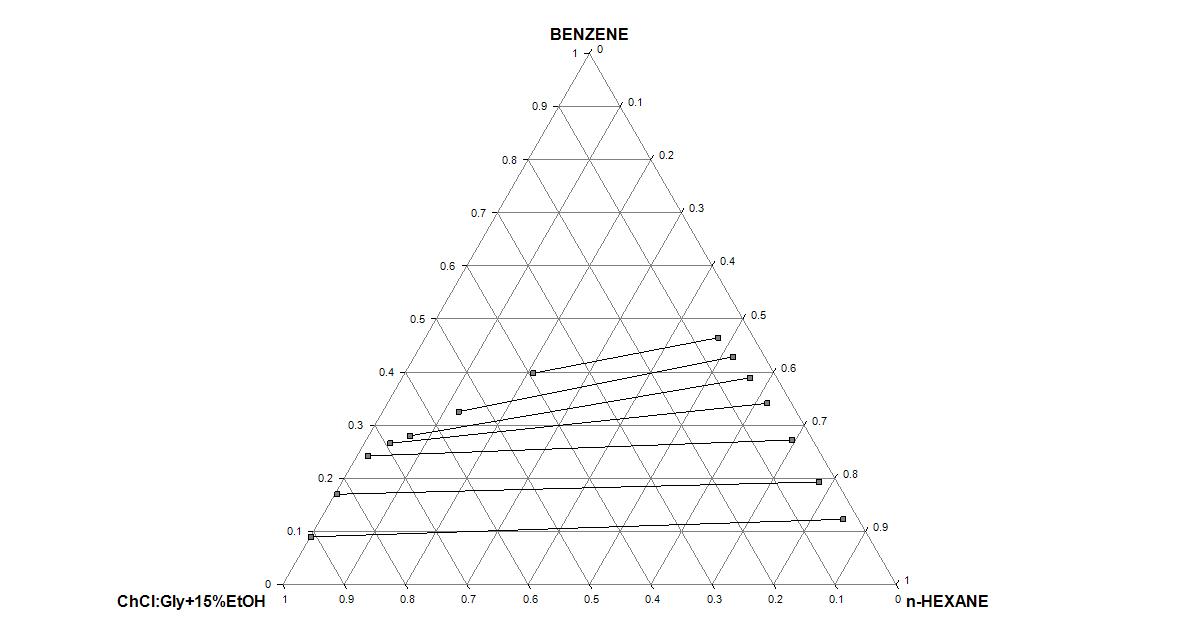


Fig. S2c: Experimental tie lines data for the studied mixture of *n-*hexane - benzene – ChCl:Gly + 15%EtOH at temperature 303K and atmospheric pressure.

**
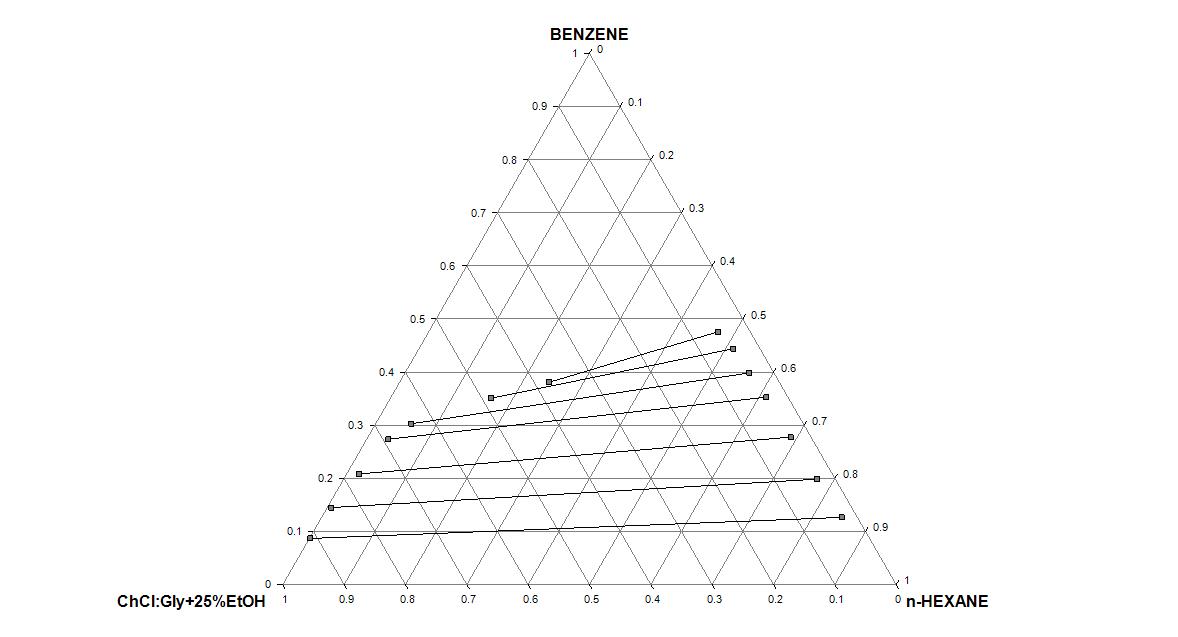
**

Fig S2d: Experimental tie lines data for the studied mixture of *n-*hexane - benzene – ChCl:Gly + 25%EtOH at temperature 303K and atmospheric pressure.

**
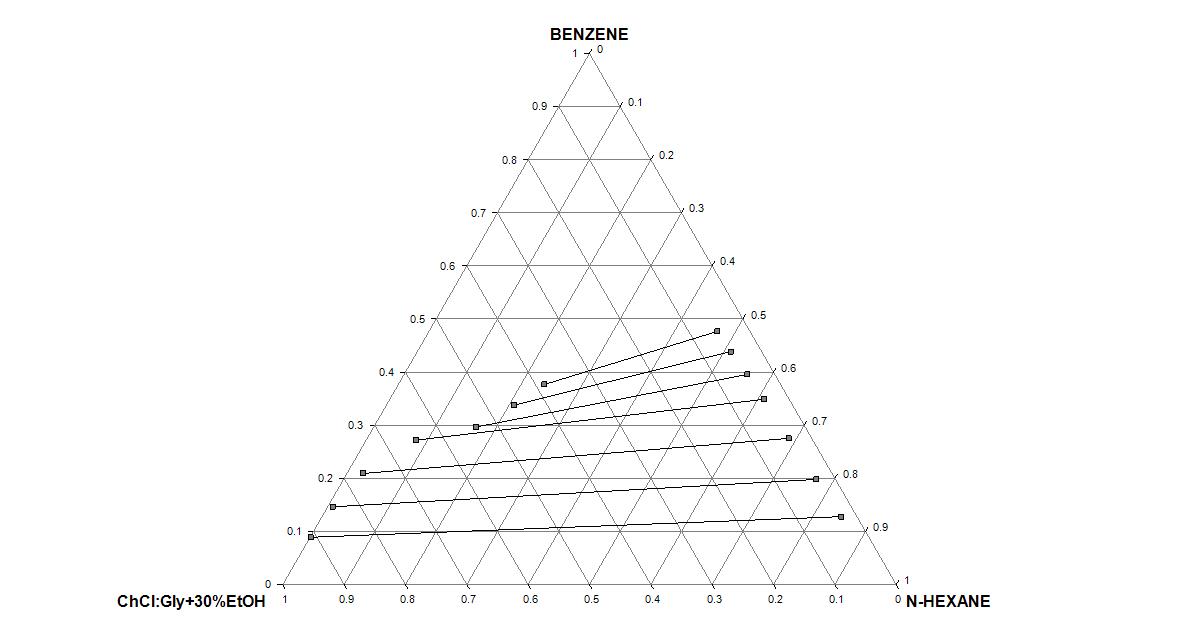
**

Fig S2e: Experimental tie lines data for the studied mixture of *n-*hexane - benzene – ChCl:Gly + 30%EtOH at temperature 303K and atmospheric pressure.

**
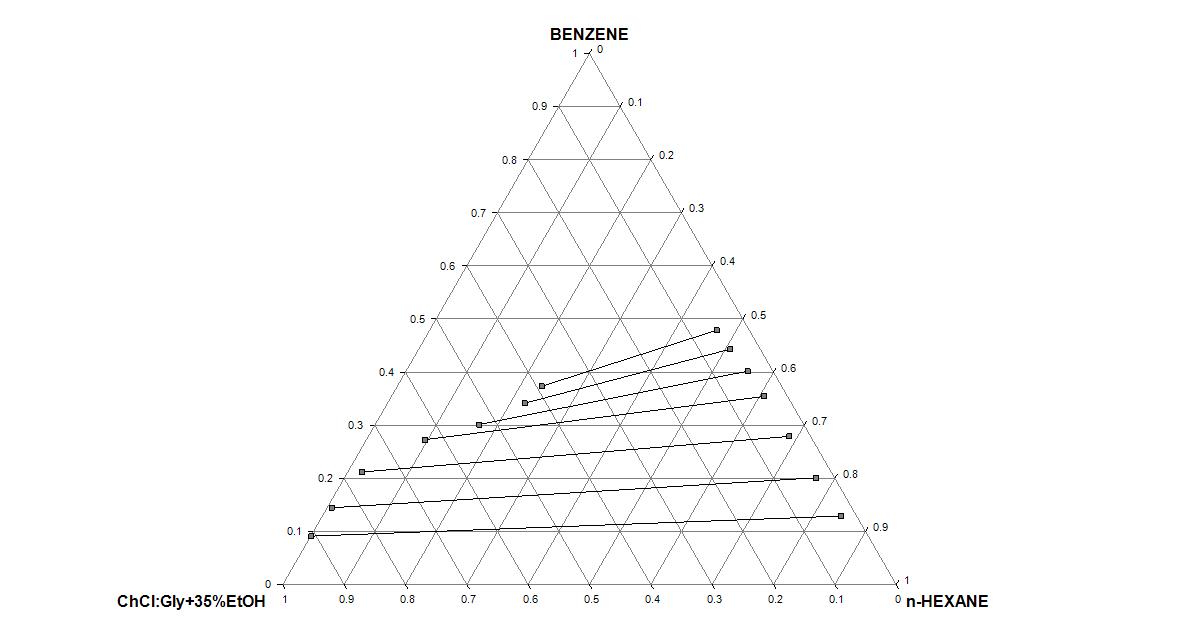
**

Fig S2f: Experimental tie lines data for the studied mixture of *n-*hexane - benzene – ChCl:Gly + 35%EtOH at temperature 303K and atmospheric pressure.


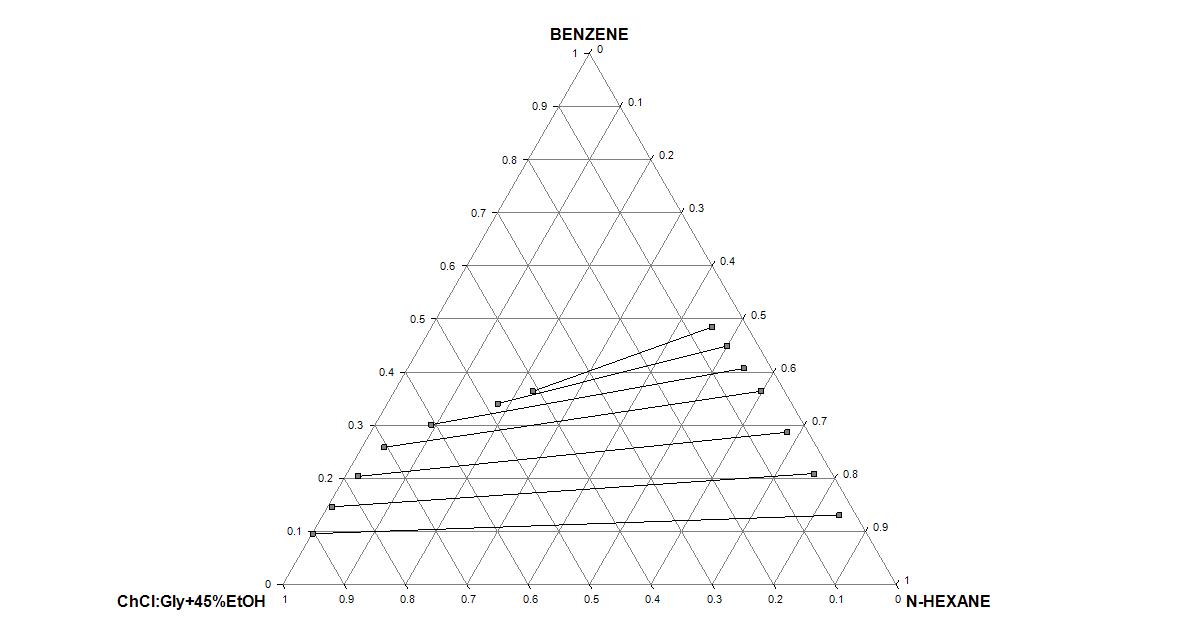


Fig S2g: Experimental tie lines data for the studied mixture of *n-*hexane - benzene – ChCl:Gly + 45%EtOH at temperature 303K and atmospheric pressure.


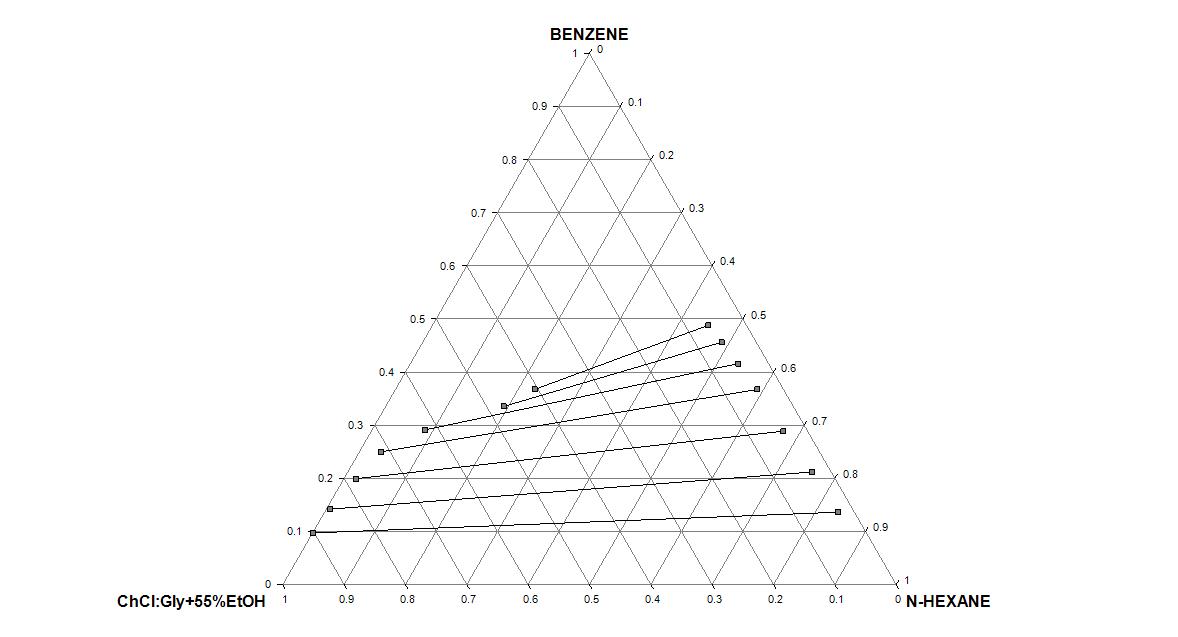


Fig S2h: Experimental tie lines data for the studied mixture of *n-*hexane - benzene – ChCl:Gly + 55%EtOH at temperature 303K and atmospheric pressure.


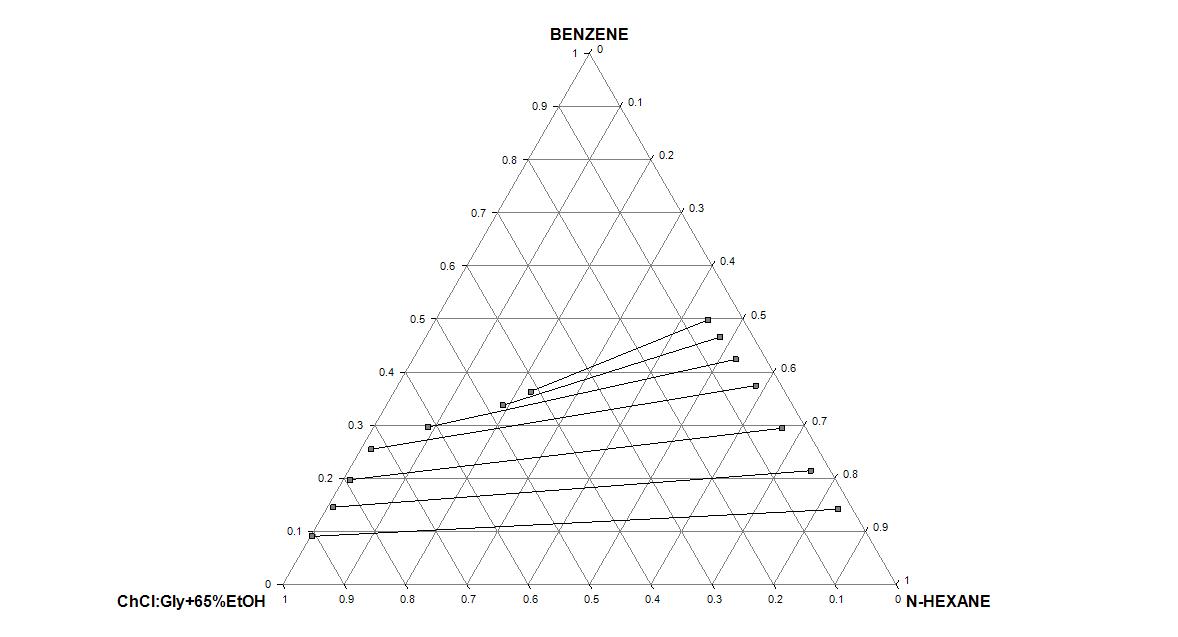


Fig S2i: Experimental tie lines data for the studied mixture of *n-*hexane - benzene – ChCl:Gly + 65%EtOH at temperature 303K and atmospheric pressure.


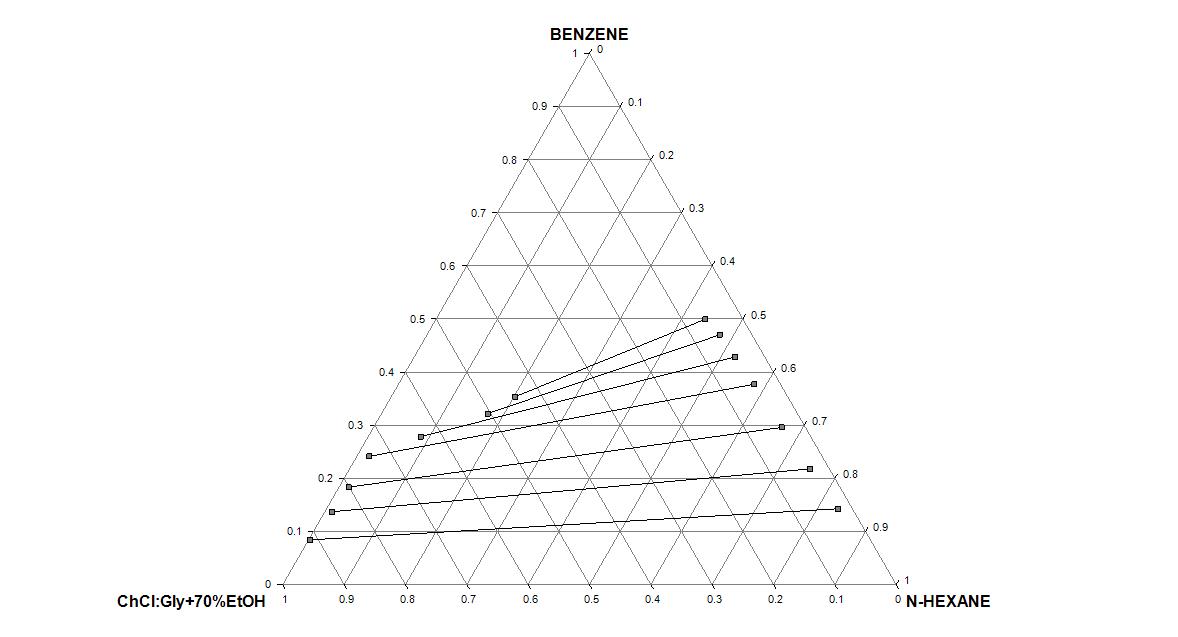


Fig S2j: Experimental tie lines data for the studied mixture of *n-*hexane - benzene – ChCl:Gly + 70%EtOH at temperature 303K and atmospheric pressure.


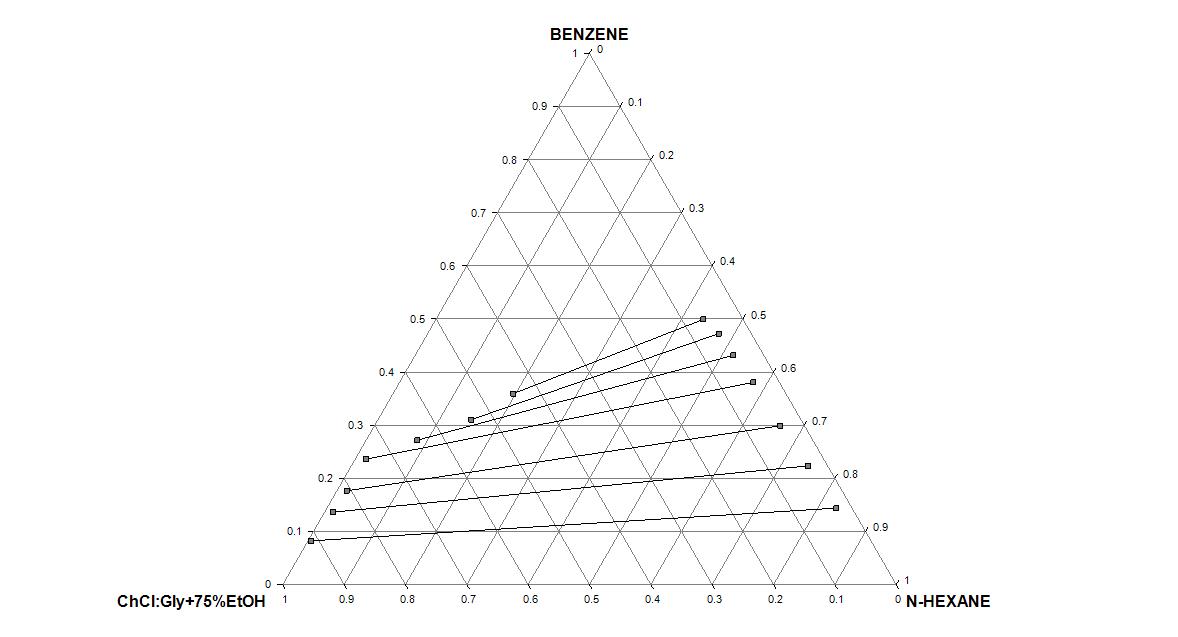


Fig S2k: Experimental tie lines data for the studied mixture of *n-*hexane - benzene – ChCl:Gly + 75%EtOH at temperature 303K and atmospheric pressure.


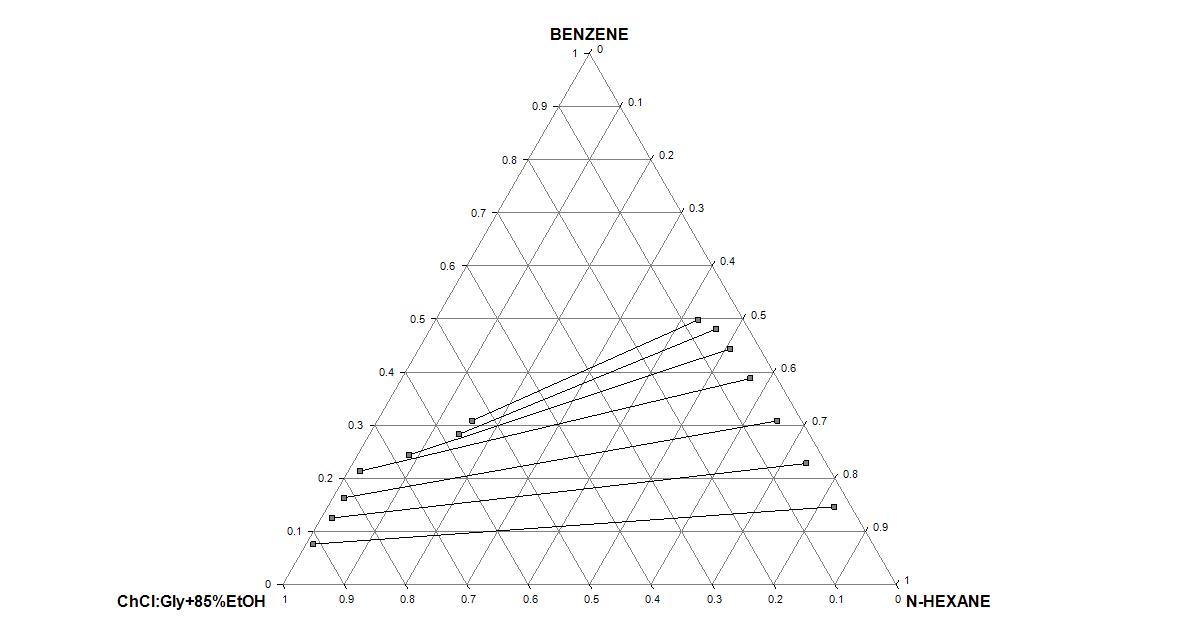


Fig S2l: Experimental tie lines data for the studied mixture of *n-*hexane - benzene – ChCl:Gly + 85%EtOH at temperature 303K and atmospheric pressure.


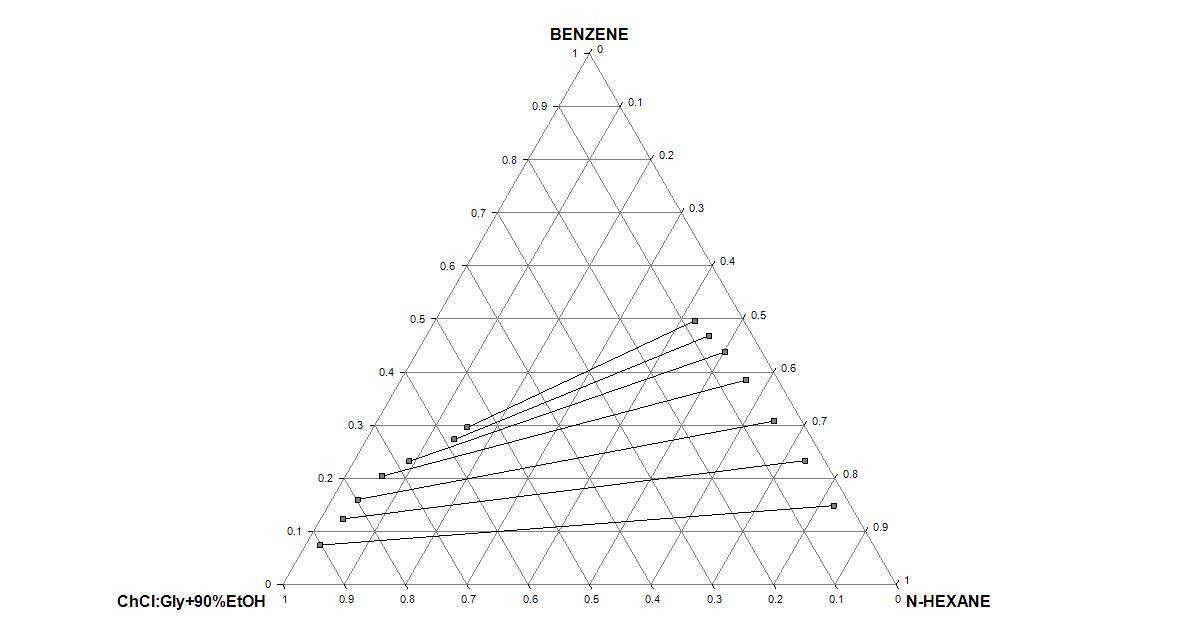


Fig S2m: Experimental tie lines data for the studied mixture of *n-*hexane - benzene – ChCl:Gly + 90%EtOH at temperature 303K and atmospheric pressure.

1. LLE data and ternary diagrams for reline/EtOH hybrid solvent

Table S4: LLE data for reline/EtOH hybrid solvent

| **Raffinate Phase** | | **Extract Phase** | | **D** | **S** |
| --- | --- | --- | --- | --- | --- |
| ***x*_11_** | ***x*_21_** | ***x*_13_** | ***x*_23_** |  |  |
| **0% EtOH** | | | | | |
| 0.8156 | 0.1844 | 0.0092 | 0.0317 | 0.172 | 15.240 |
| 0.6864 | 0.3136 | 0.0151 | 0.1013 | 0.323 | 14.684 |
| 0.6150 | 0.3850 | 0.0139 | 0.1241 | 0.322 | 14.262 |
| 0.5396 | 0.4604 | 0.0178 | 0.1395 | 0.303 | 9.185 |
| 0.4545 | 0.5455 | 0.0263 | 0.1886 | 0.346 | 5.975 |
| 0.3571 | 0.6429 | 0.0331 | 0.1976 | 0.307 | 3.316 |
| 0.2598 | 0.7402 | 0.0414 | 0.2007 | 0.271 | 1.702 |
|  |  |  |  |  |  |
| **5% EtOH** | | | | | |
| 0.8143 | 0.1762 | 0.0098 | 0.0423 | 0.240 | 19.948 |
| 0.7099 | 0.2713 | 0.0131 | 0.0701 | 0.258 | 14.002 |
| 0.6321 | 0.3391 | 0.0139 | 0.0980 | 0.289 | 13.142 |
| 0.5946 | 0.3854 | 0.0178 | 0.1041 | 0.270 | 9.023 |
| 0.4885 | 0.4795 | 0.0263 | 0.1486 | 0.310 | 5.756 |
| 0.3730 | 0.5875 | 0.0397 | 0.1939 | 0.330 | 3.101 |
| 0.3141 | 0.6334 | 0.0459 | 0.2217 | 0.350 | 2.395 |
|  |  |  |  |  |  |
| **10% EtOH** | | | | | |
| 0.8115 | 0.1785 | 0.0080 | 0.0446 | 0.250 | 25.345 |
| 0.7176 | 0.2669 | 0.0125 | 0.0614 | 0.230 | 13.207 |
| 0.6607 | 0.3168 | 0.0173 | 0.0887 | 0.280 | 10.693 |
| 0.5886 | 0.3714 | 0.0311 | 0.1114 | 0.300 | 5.677 |
| 0.4840 | 0.4715 | 0.0335 | 0.1415 | 0.300 | 4.336 |
| 0.3684 | 0.5786 | 0.0401 | 0.1967 | 0.340 | 3.123 |
| 0.3120 | 0.6325 | 0.0514 | 0.2214 | 0.350 | 2.125 |
|  |  |  |  |  |  |
| **15% EtOH** | | | | | |
| 0.8188 | 0.1712 | 0.0093 | 0.0531 | 0.310 | 27.308 |
| 0.7225 | 0.2600 | 0.0135 | 0.0728 | 0.280 | 14.985 |
| 0.6755 | 0.2995 | 0.0212 | 0.0869 | 0.290 | 9.245 |
| 0.5993 | 0.3607 | 0.0386 | 0.1226 | 0.340 | 5.277 |
| 0.4986 | 0.4479 | 0.0407 | 0.1612 | 0.360 | 4.409 |
| 0.3772 | 0.5623 | 0.0524 | 0.2025 | 0.360 | 2.592 |
| 0.3242 | 0.6068 | 0.0636 | 0.2302 | 0.379 | 1.934 |
|  |  |  |  |  |  |
| **20% EtOH** | | | | | |
| 0.8207 | 0.1693 | 0.0082 | 0.0542 | 0.320 | 32.042 |
| 0.7253 | 0.2594 | 0.0123 | 0.0744 | 0.287 | 16.913 |
| 0.6781 | 0.2794 | 0.0235 | 0.0895 | 0.320 | 9.243 |
| 0.6005 | 0.3465 | 0.0389 | 0.1178 | 0.340 | 5.248 |
| 0.5112 | 0.4283 | 0.0458 | 0.1542 | 0.360 | 4.018 |
| 0.3724 | 0.5676 | 0.0525 | 0.2101 | 0.370 | 2.626 |
| 0.3288 | 0.5987 | 0.0679 | 0.2335 | 0.390 | 1.889 |
|  |  |  |  |  |  |
| **25% EtOH** | | | | | |
| 0.8232 | 0.1688 | 0.0077 | 0.0523 | 0.310 | 33.124 |
| 0.7301 | 0.2549 | 0.0103 | 0.0688 | 0.270 | 19.132 |
| 0.6795 | 0.2930 | 0.0195 | 0.0879 | 0.300 | 10.454 |
| 0.6026 | 0.3574 | 0.0358 | 0.1144 | 0.320 | 5.388 |
| 0.5144 | 0.4331 | 0.0474 | 0.1473 | 0.340 | 3.691 |
| 0.3758 | 0.5617 | 0.0588 | 0.2022 | 0.360 | 2.301 |
| 0.3297 | 0.5993 | 0.0701 | 0.2338 | 0.390 | 1.835 |
|  |  |  |  |  |  |
| **30% EtOH** | | | | | |
| 0.8256 | 0.1663 | 0.0077 | 0.0549 | 0.330 | 35.396 |
| 0.7323 | 0.2532 | 0.0125 | 0.0761 | 0.301 | 17.608 |
| 0.6805 | 0.2970 | 0.0171 | 0.0951 | 0.320 | 12.743 |
| 0.6060 | 0.3595 | 0.0236 | 0.1258 | 0.350 | 8.986 |
| 0.5178 | 0.4373 | 0.0318 | 0.1618 | 0.370 | 6.025 |
| 0.3783 | 0.5682 | 0.0424 | 0.2103 | 0.370 | 3.302 |
| 0.3301 | 0.5999 | 0.0703 | 0.2401 | 0.400 | 1.879 |
|  |  |  |  |  |  |
| **35% EtOH** | | | | | |
| 0.8272 | 0.1651 | 0.0066 | 0.0528 | 0.320 | 40.082 |
| 0.7345 | 0.2525 | 0.0093 | 0.0733 | 0.290 | 22.927 |
| 0.6824 | 0.2976 | 0.0163 | 0.0982 | 0.330 | 13.814 |
| 0.6091 | 0.3634 | 0.0187 | 0.1272 | 0.350 | 11.401 |
| 0.5202 | 0.4443 | 0.0246 | 0.1644 | 0.370 | 7.825 |
| 0.3796 | 0.5779 | 0.0478 | 0.2196 | 0.380 | 3.018 |
| 0.3331 | 0.6065 | 0.0675 | 0.2426 | 0.400 | 1.974 |
|  |  |  |  |  |  |
| **40% EtOH** | | | | | |
| 0.8295 | 0.1630 | 0.0062 | 0.0505 | 0.310 | 41.450 |
| 0.7401 | 0.2449 | 0.0083 | 0.0637 | 0.260 | 23.193 |
| 0.6913 | 0.2862 | 0.0126 | 0.0888 | 0.310 | 17.023 |
| 0.5946 | 0.3654 | 0.0172 | 0.1206 | 0.330 | 11.410 |
| 0.5268 | 0.4227 | 0.0294 | 0.1479 | 0.350 | 6.270 |
| 0.3824 | 0.5581 | 0.0435 | 0.2009 | 0.360 | 3.164 |
| 0.3335 | 0.5990 | 0.0507 | 0.2205 | 0.368 | 2.421 |
|  |  |  |  |  |  |
| **45% EtOH** | | | | | |
| 0.8301 | 0.1621 | 0.0056 | 0.0471 | 0.291 | 43.071 |
| 0.7426 | 0.2414 | 0.0184 | 0.0628 | 0.260 | 10.499 |
| 0.6858 | 0.2892 | 0.0279 | 0.0867 | 0.300 | 7.369 |
| 0.5980 | 0.3605 | 0.0393 | 0.1191 | 0.330 | 5.027 |
| 0.5175 | 0.4300 | 0.0449 | 0.1505 | 0.350 | 4.034 |
| 0.4063 | 0.5317 | 0.0597 | 0.1967 | 0.370 | 2.518 |
| 0.3374 | 0.5913 | 0.0714 | 0.2246 | 0.380 | 1.795 |
|  |  |  |  |  |  |
| **50% EtOH** | | | | | |
| 0.8327 | 0.1605 | 0.0052 | 0.0452 | 0.282 | 45.097 |
| 0.7474 | 0.2351 | 0.0144 | 0.0589 | 0.251 | 13.003 |
| 0.6892 | 0.2873 | 0.0265 | 0.0833 | 0.290 | 7.541 |
| 0.5945 | 0.3705 | 0.0387 | 0.1149 | 0.310 | 4.764 |
| 0.5201 | 0.4324 | 0.0492 | 0.1427 | 0.330 | 3.489 |
| 0.4327 | 0.4859 | 0.0597 | 0.1903 | 0.392 | 2.839 |
| 0.3769 | 0.5461 | 0.0678 | 0.2306 | 0.422 | 2.347 |
|  |  |  |  |  |  |
| **55% EtOH** | | | | | |
| 0.8292 | 0.1583 | 0.0072 | 0.0523 | 0.330 | 38.049 |
| 0.7845 | 0.1650 | 0.0186 | 0.0661 | 0.401 | 16.897 |
| 0.7119 | 0.2079 | 0.0393 | 0.0868 | 0.418 | 7.563 |
| 0.6084 | 0.2901 | 0.0535 | 0.1074 | 0.370 | 4.210 |
| 0.5446 | 0.3379 | 0.0699 | 0.1318 | 0.390 | 3.039 |
| 0.4858 | 0.3837 | 0.0863 | 0.1497 | 0.390 | 2.196 |
| 0.4001 | 0.4627 | 0.1065 | 0.2035 | 0.440 | 1.652 |
|  |  |  |  |  |  |
| **60% EtOH** | | | | | |
| 0.8334 | 0.1521 | 0.0103 | 0.0532 | 0.350 | 28.301 |
| 0.7759 | 0.1753 | 0.0367 | 0.0601 | 0.343 | 7.248 |
| 0.7171 | 0.2034 | 0.0786 | 0.0753 | 0.370 | 3.378 |
| 0.6125 | 0.2865 | 0.1002 | 0.1089 | 0.380 | 2.323 |
| 0.5578 | 0.3278 | 0.1345 | 0.1266 | 0.386 | 1.602 |
| 0.4903 | 0.3667 | 0.1511 | 0.1503 | 0.410 | 1.330 |
| 0.4040 | 0.4455 | 0.1625 | 0.2005 | 0.450 | 1.119 |
|  |  |  |  |  |  |
| **65% EtOH** | | | | | |
| 0.8358 | 0.1504 | 0.0192 | 0.0511 | 0.340 | 14.790 |
| 0.7776 | 0.1621 | 0.0319 | 0.0623 | 0.384 | 9.369 |
| 0.7199 | 0.1913 | 0.0543 | 0.0727 | 0.380 | 5.038 |
| 0.6180 | 0.2595 | 0.0963 | 0.1012 | 0.390 | 2.503 |
| 0.5527 | 0.3075 | 0.1211 | 0.1231 | 0.400 | 1.827 |
| 0.4934 | 0.3597 | 0.1525 | 0.1475 | 0.410 | 1.327 |
| 0.4088 | 0.4413 | 0.1677 | 0.2030 | 0.460 | 1.121 |
|  |  |  |  |  |  |
| **70% EtOH** | | | | | |
| 0.8376 | 0.1479 | 0.0236 | 0.0503 | 0.340 | 12.070 |
| 0.7934 | 0.1621 | 0.0473 | 0.0642 | 0.396 | 6.643 |
| 0.7221 | 0.1936 | 0.0735 | 0.0868 | 0.448 | 4.405 |
| 0.6549 | 0.2412 | 0.1122 | 0.1169 | 0.485 | 2.829 |
| 0.5555 | 0.3139 | 0.1371 | 0.1358 | 0.433 | 1.753 |
| 0.4947 | 0.3596 | 0.1523 | 0.1640 | 0.456 | 1.481 |
| 0.4109 | 0.4398 | 0.1859 | 0.2023 | 0.460 | 1.017 |
|  |  |  |  |  |  |
| **75% EtOH** | | | | | |
| 0.8388 | 0.1483 | 0.0251 | 0.0489 | 0.330 | 11.019 |
| 0.7950 | 0.1575 | 0.0394 | 0.0605 | 0.384 | 7.751 |
| 0.7243 | 0.1973 | 0.0637 | 0.0868 | 0.440 | 5.002 |
| 0.6206 | 0.2816 | 0.0981 | 0.1071 | 0.380 | 2.406 |
| 0.5582 | 0.3153 | 0.1256 | 0.1358 | 0.431 | 1.914 |
| 0.5005 | 0.3639 | 0.1478 | 0.1696 | 0.466 | 1.578 |
| 0.4131 | 0.4444 | 0.1465 | 0.2073 | 0.466 | 1.315 |
|  |  |  |  |  |  |
| **80% EtOH** | | | | | |
| 0.8399 | 0.1452 | 0.0315 | 0.0494 | 0.340 | 9.071 |
| 0.7965 | 0.1585 | 0.0515 | 0.0623 | 0.393 | 6.079 |
| 0.7261 | 0.1938 | 0.0665 | 0.0717 | 0.370 | 4.040 |
| 0.6501 | 0.2543 | 0.0901 | 0.0962 | 0.378 | 2.730 |
| 0.6002 | 0.2921 | 0.1154 | 0.1301 | 0.445 | 2.317 |
| 0.5043 | 0.3601 | 0.1433 | 0.1477 | 0.410 | 1.443 |
| 0.4431 | 0.3974 | 0.1861 | 0.2006 | 0.505 | 1.202 |
|  |  |  |  |  |  |
| **85% EtOH** | | | | | |
| 0.8007 | 0.1452 | 0.0382 | 0.0567 | 0.390 | 8.185 |
| 0.7524 | 0.1734 | 0.0729 | 0.0717 | 0.413 | 4.268 |
| 0.7261 | 0.1938 | 0.0823 | 0.0887 | 0.458 | 4.038 |
| 0.6501 | 0.2543 | 0.1001 | 0.1150 | 0.452 | 2.937 |
| 0.6002 | 0.2920 | 0.1154 | 0.1301 | 0.446 | 2.317 |
| 0.5043 | 0.3601 | 0.1262 | 0.1640 | 0.455 | 1.820 |
| 0.4431 | 0.3975 | 0.1448 | 0.2148 | 0.540 | 1.654 |
|  |  |  |  |  |  |
| **90% EtOH** | | | | | |
| 0.8001 | 0.0969 | 0.0855 | 0.0681 | 0.703 | 6.577 |
| 0.7237 | 0.1885 | 0.1227 | 0.0830 | 0.440 | 2.597 |
| 0.6989 | 0.2058 | 0.1321 | 0.1001 | 0.486 | 2.573 |
| 0.6596 | 0.2318 | 0.1408 | 0.1282 | 0.553 | 2.591 |
| 0.6080 | 0.2732 | 0.1512 | 0.1433 | 0.525 | 2.109 |
| 0.5147 | 0.3391 | 0.1590 | 0.1734 | 0.511 | 1.655 |
| 0.4431 | 0.3975 | 0.1621 | 0.2129 | 0.536 | 1.464 |
|  |  |  |  |  |  |
| **95% EtOH** | | | | | |
| 0.8001 | 0.1069 | 0.1081 | 0.0736 | 0.688 | 5.096 |
| 0.7506 | 0.1508 | 0.1829 | 0.0962 | 0.638 | 2.618 |
| 0.7146 | 0.1772 | 0.1946 | 0.1150 | 0.649 | 2.383 |
| 0.6735 | 0.2073 | 0.2020 | 0.1395 | 0.673 | 2.244 |
| 0.6314 | 0.2393 | 0.2126 | 0.1641 | 0.686 | 2.037 |
| 0.5824 | 0.2656 | 0.2204 | 0.1941 | 0.731 | 1.931 |
| 0.4870 | 0.2901 | 0.2230 | 0.2280 | 0.786 | 1.716 |


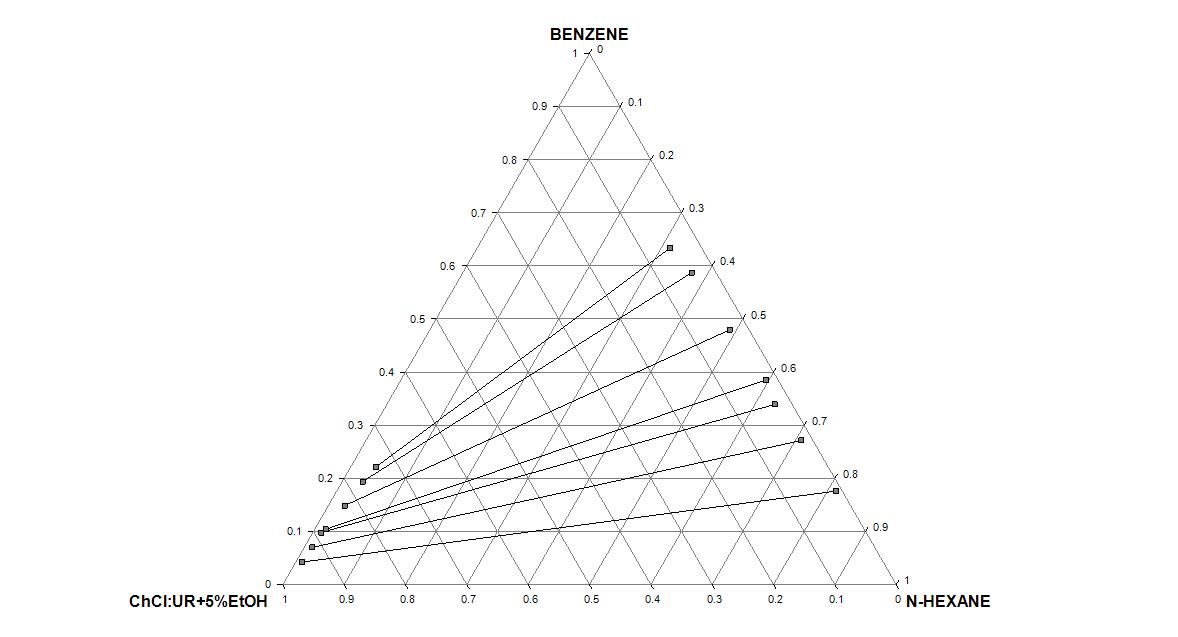


Fig S3a: Experimental tie lines data for the studied mixture of *n-*hexane - benzene – ChCl:Ur + 5%EtOH at temperature 303K and atmospheric pressure.


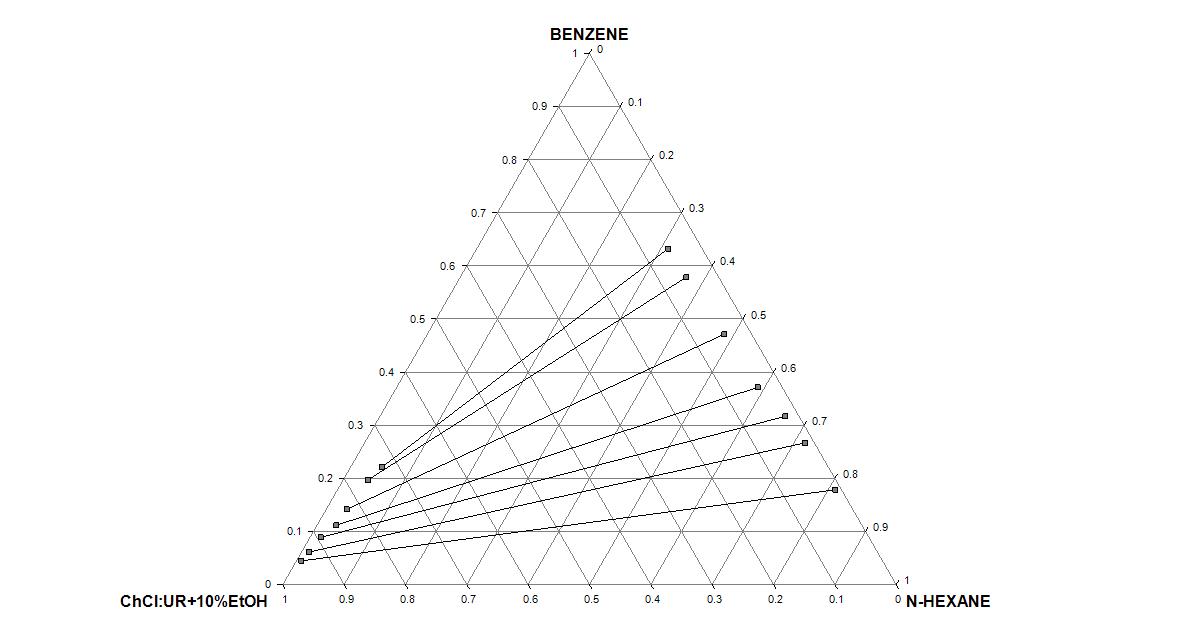


Fig S3b: Experimental tie lines data for the studied mixture of *n-*hexane - benzene – ChCl:Ur + 10%EtOH at temperature 303K and atmospheric pressure.


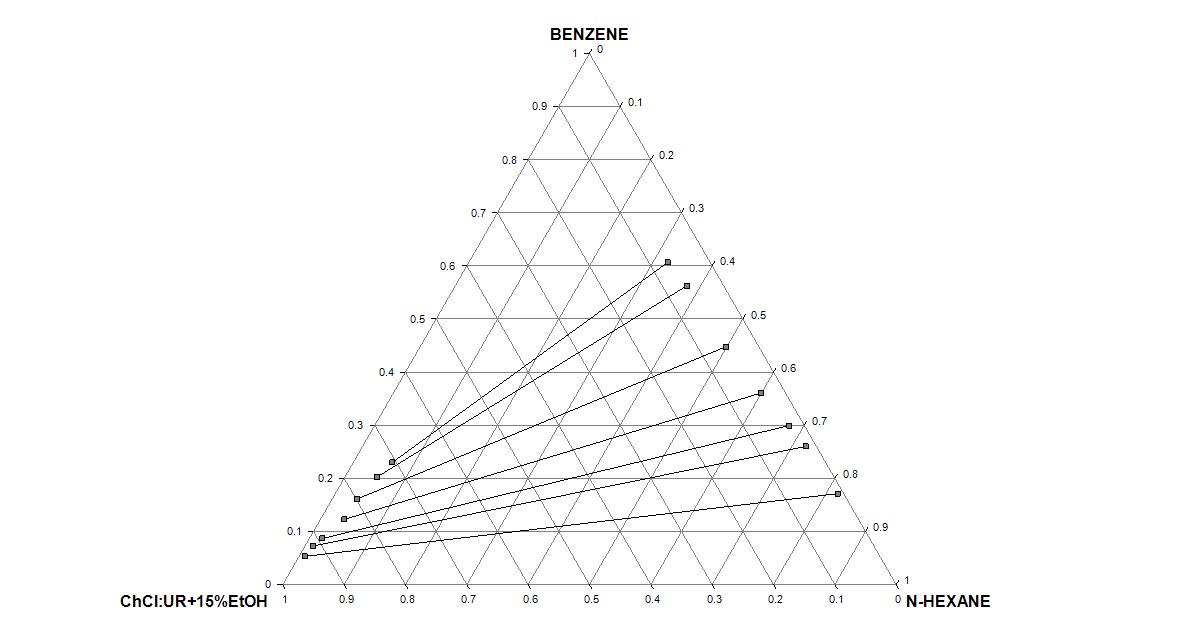


Fig S3c: Experimental tie lines data for the studied mixture of *n-*hexane - benzene –ChCl:Ur + 15%EtOH at temperature 303K and atmospheric pressure.


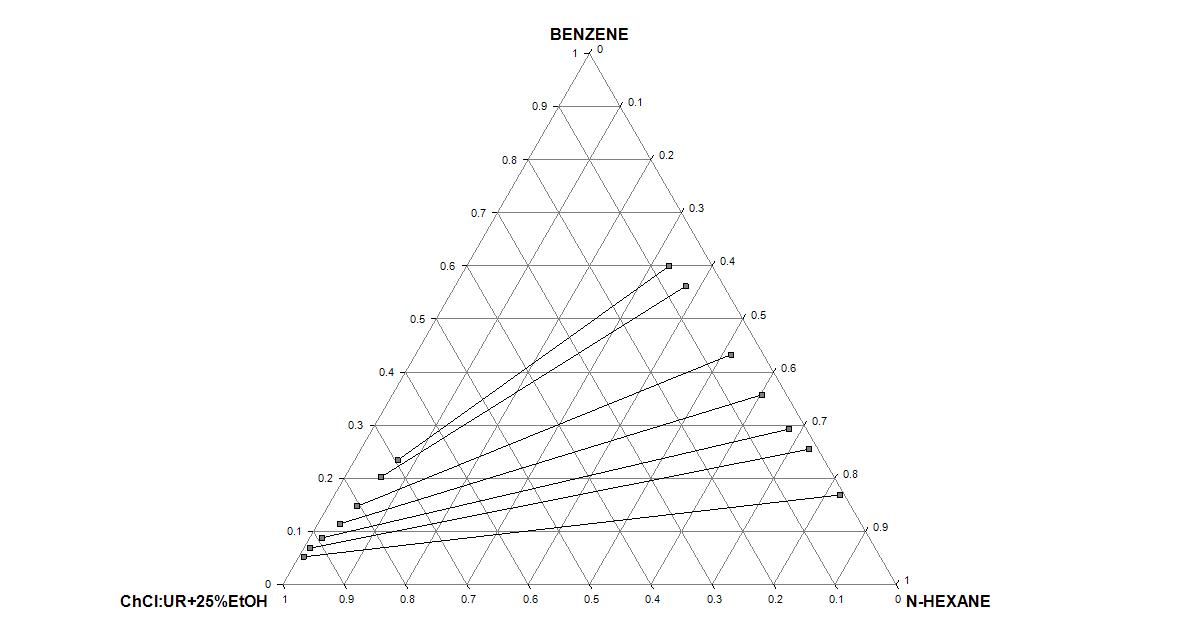


Fig S3d: Experimental tie lines data for the studied mixture of *n-*hexane - benzene –ChCl:Ur + 25%EtOH at temperature 303K and atmospheric pressure.


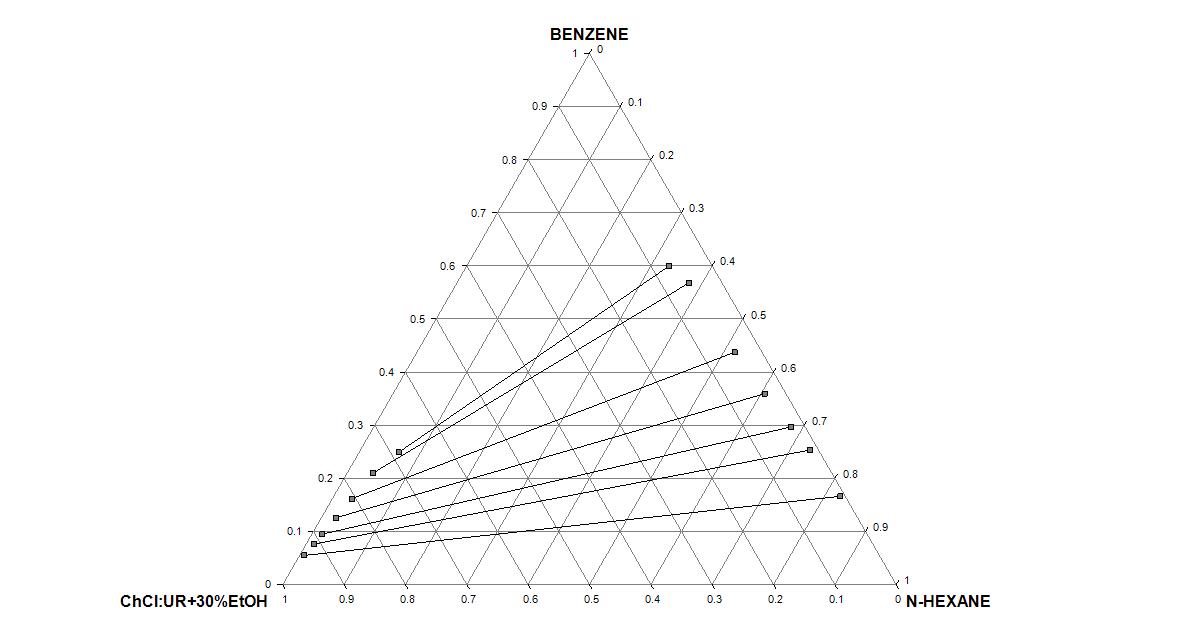


Fig S3e: Experimental tie lines data for the studied mixture of *n-*hexane - benzene – ChCl:Ur + 30%EtOH at temperature 303K and atmospheric pressure.


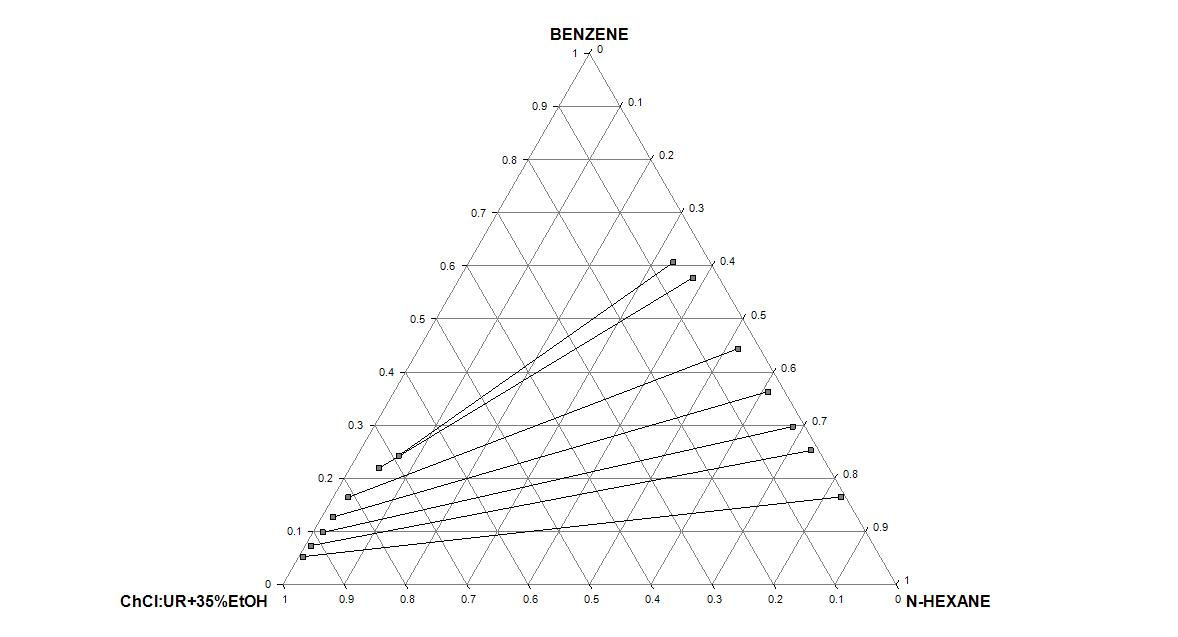


Fig S3f: Experimental tie lines data for the studied mixture of *n-*hexane - benzene – ChCl:Ur + 35%EtOH at temperature 303K and atmospheric pressure.


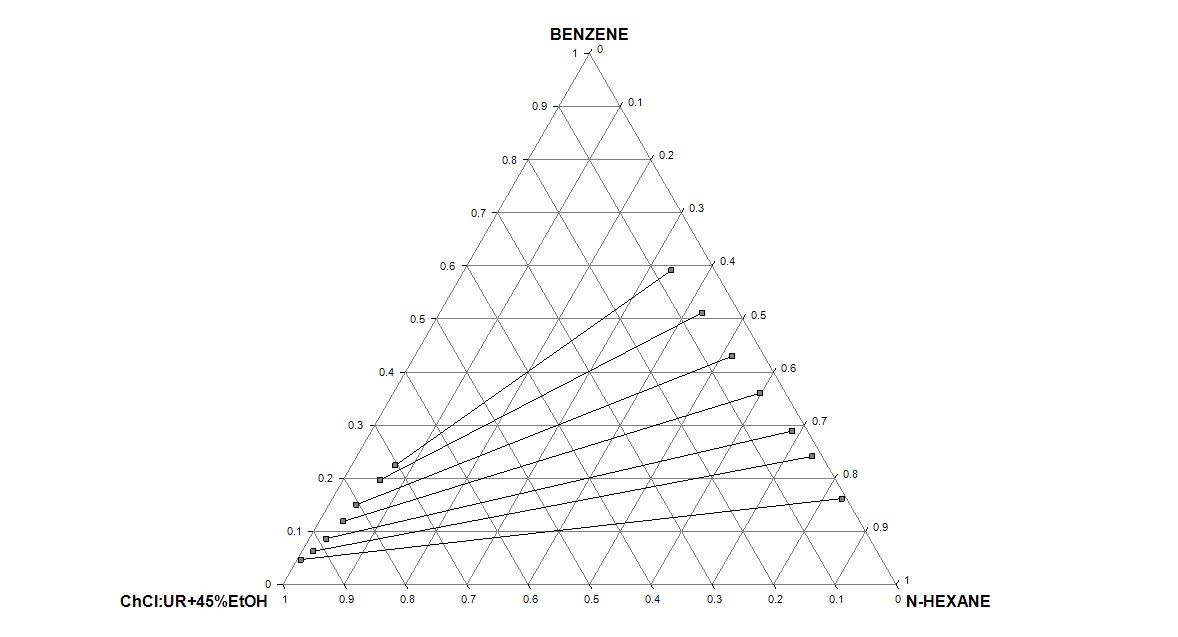


Fig S3g: Experimental tie lines data for the studied mixture of *n-*hexane - benzene – ChCl:Ur + 45%EtOH at temperature 303K and atmospheric pressure.


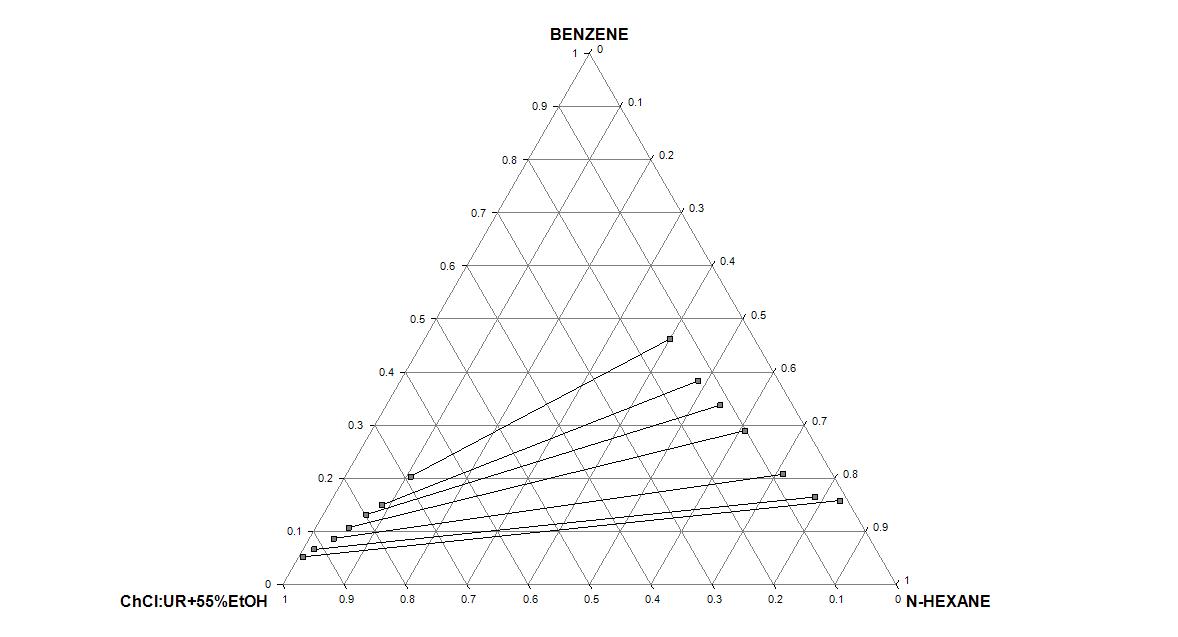


Fig S3h: Experimental tie lines data for the studied mixture of *n-*hexane - benzene – ChCl:Ur + 55%EtOH at temperature 303K and atmospheric pressure.


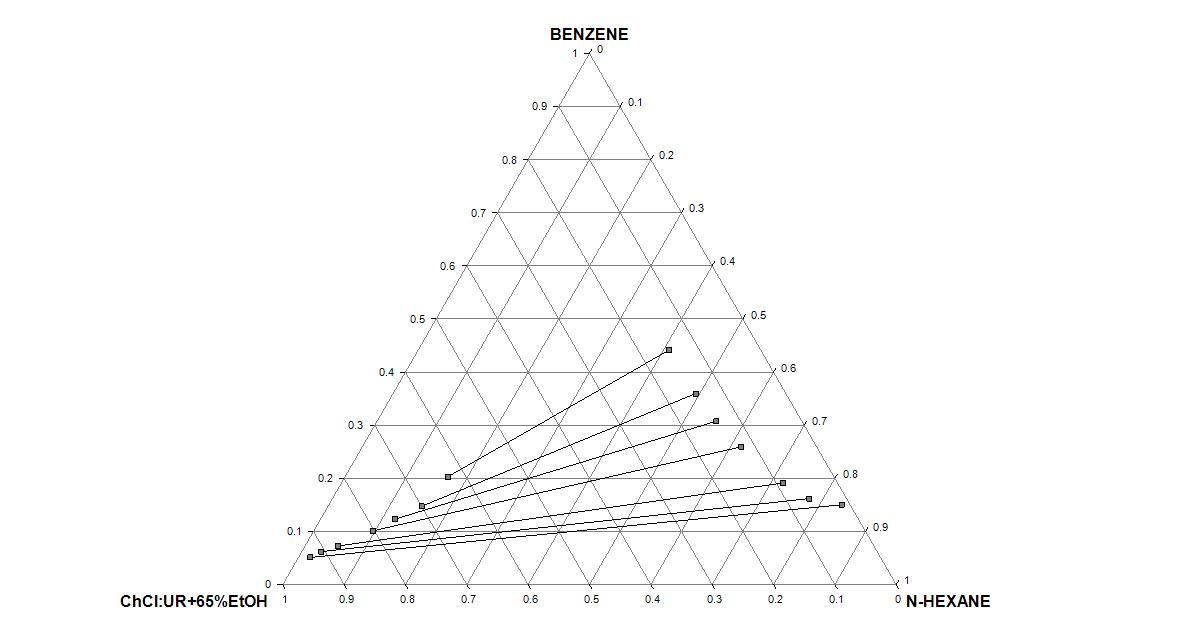


Fig S3i: Experimental tie lines data for the studied mixture of *n-*hexane - benzene – ChCl:Ur + 65%EtOH at temperature 303K and atmospheric pressure.


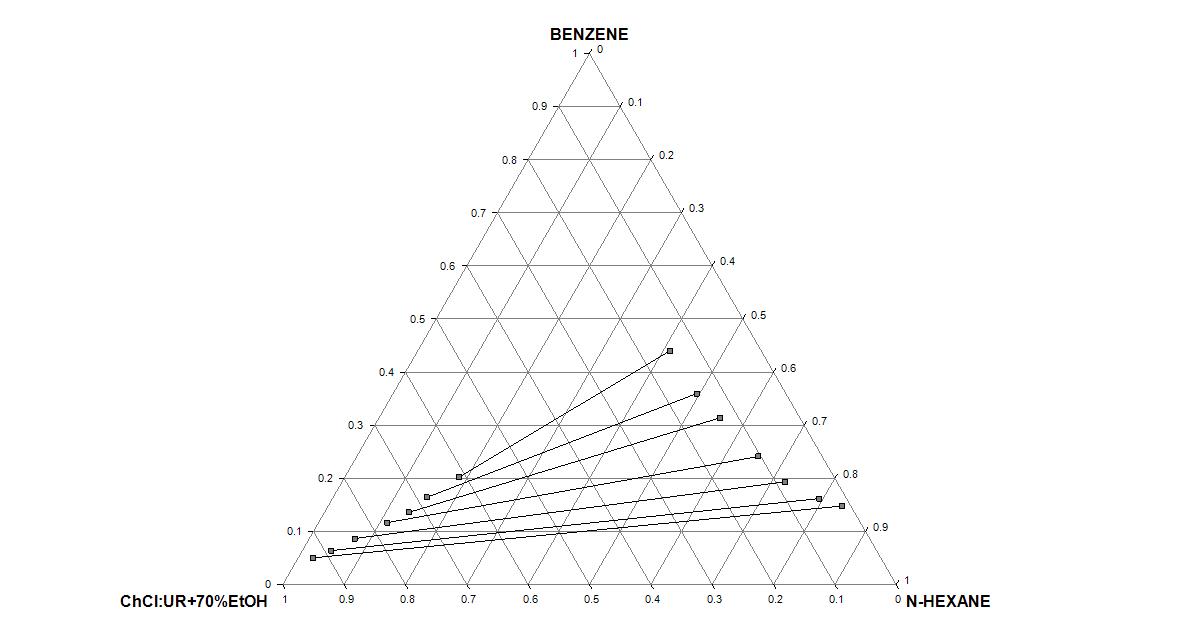


Fig S3j: Experimental tie lines data for the studied mixture of *n-*hexane - benzene – ChCl:Ur + 70%EtOH at temperature 303K and atmospheric pressure.


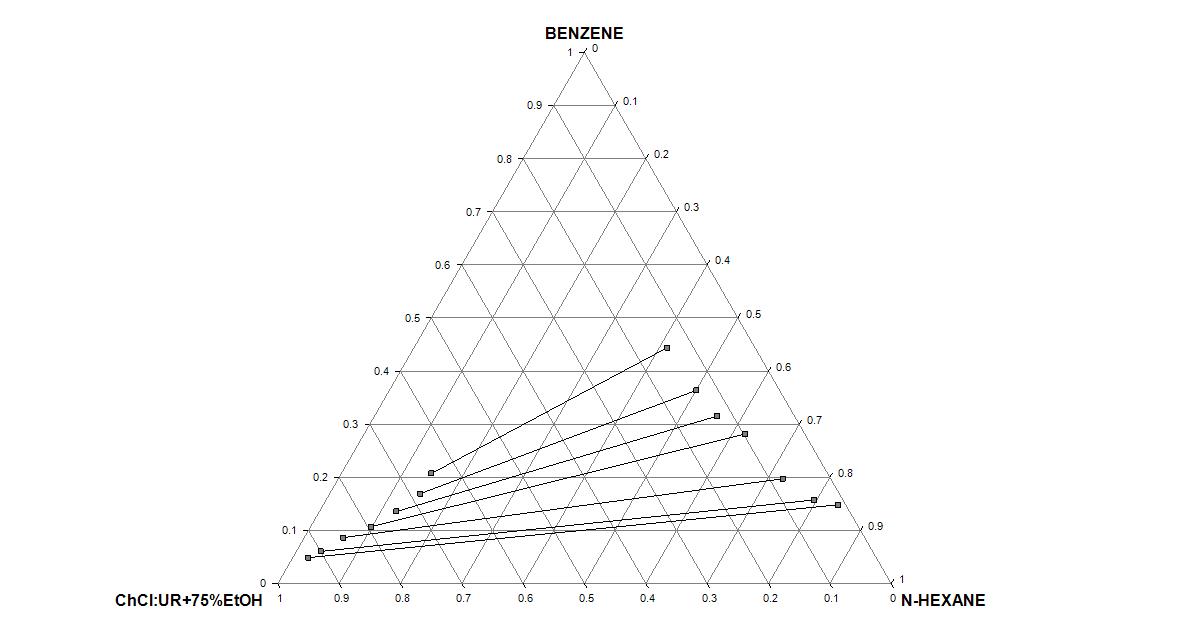


Fig S3k: Experimental tie lines data for the studied mixture of *n-*hexane - benzene – ChCl:Ur + 75%EtOH at temperature 303K and atmospheric pressure.


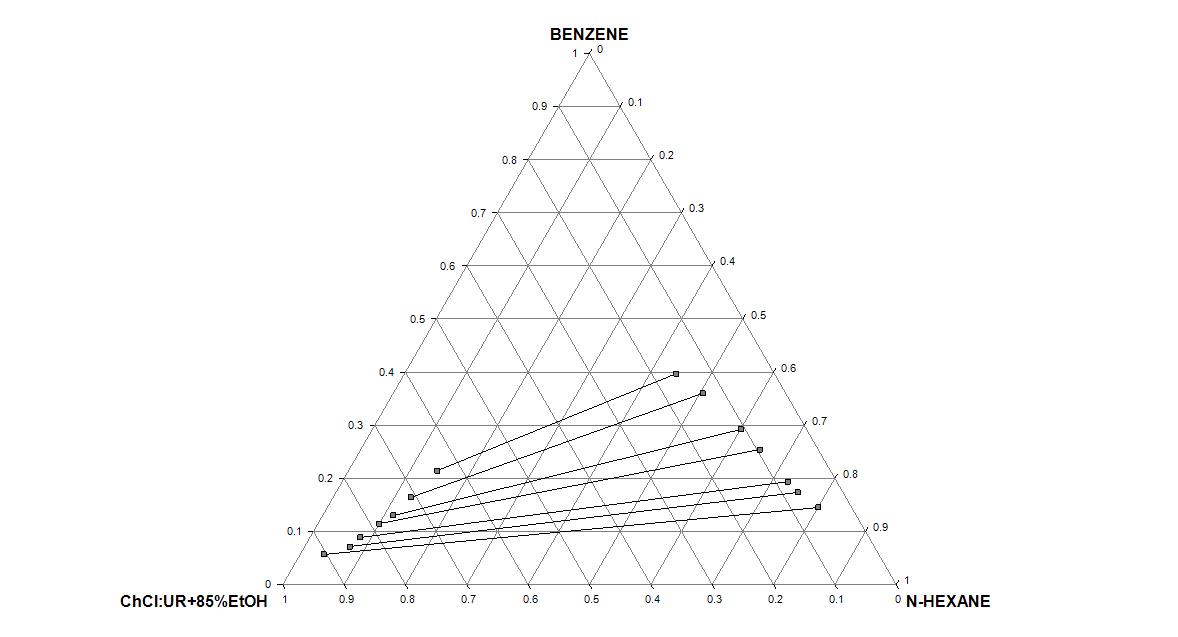


Fig S3l: Experimental tie lines data for the studied mixture of *n-*hexane - benzene – ChCl:Ur + 85%EtOH at temperature 303K and atmospheric pressure.


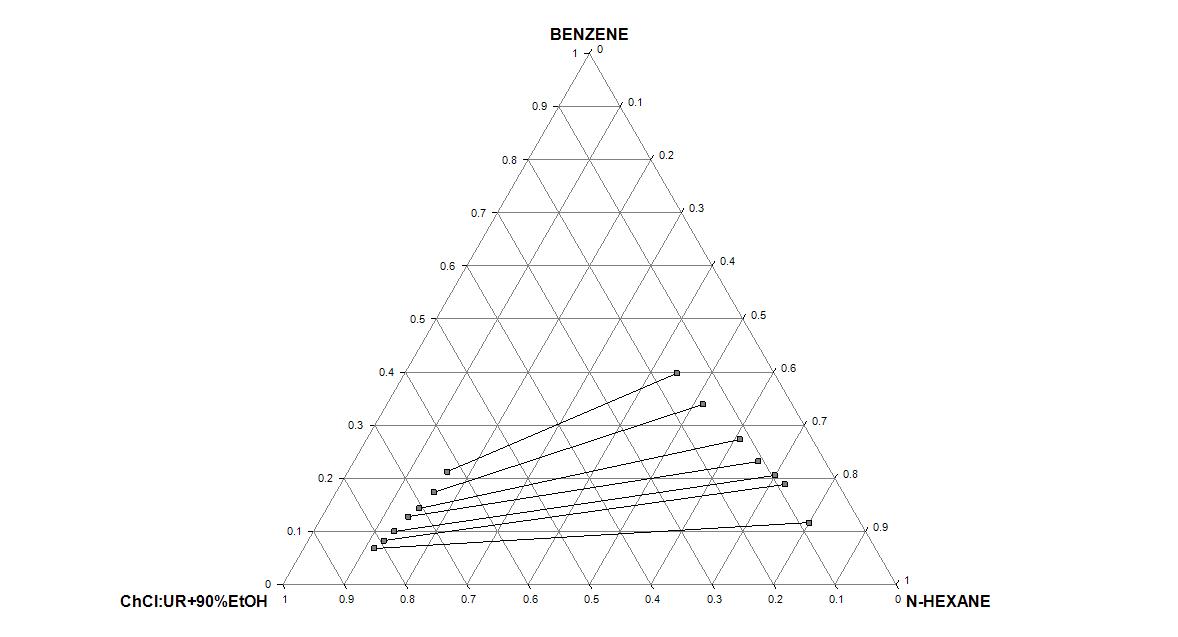


Fig S3m: Experimental tie lines data for the studied mixture of *n-*hexane - benzene – ChCl:Ur + 90%EtOH at temperature 303K and atmospheric pressure.

Table S5: Physicochemical data for ethaline/EtOH hybrid solvent

| **% EtOH** | **Density g/cm^3^** | **Viscosity** | **Refractive Index** |
| --- | --- | --- | --- |
|  |  | **cP** |  |
| 0 | 1.12 | 35.5 | 1.4814 |
| 5 | 1.12 | 34.07 | 1.4775 |
| 10 | 1.13 | 31.73 | 1.4701 |
| 15 | 1.13 | 28.82 | 1.4663 |
| 20 | 1.14 | 28.15 | 1.4586 |
| 25 | 1.14 | 27.22 | 1.4544 |
| 30 | 1.14 | 26.97 | 1.4478 |
| 35 | 1.15 | 26.45 | 1.4432 |
| 40 | 1.15 | 26.01 | 1.4375 |
| 45 | 1.16 | 25.53 | 1.4312 |
| 50 | 1.15 | 24.79 | 1.4293 |
| 55 | 1.13 | 24.46 | 1.4228 |
| 60 | 1.11 | 22.88 | 1.4169 |
| 65 | 1.1 | 17.03 | 1.4107 |
| 70 | 1.07 | 13.34 | 1.3945 |
| 75 | 1.01 | 11.95 | 1.3898 |
| 80 | 0.99 | 8.23 | 1.3827 |
| 85 | 0.92 | 5.05 | 1.3813 |
| 90 | 0.89 | 3.01 | 1.3757 |
| 95 | 0.85 | 1.8 | 1.3735 |
| 100 | 0.79 | 1.19 | 1.3617 |

Table S6: Physicochemical data for glyceline/EtOH hybrid solvent

| **% EtOH** | **Density g/cm^3^** | **Viscosity** | **Refractive Index** |
| --- | --- | --- | --- |
|  |  | **cP** |  |
| 0 | 1.18 | 330.4 | 1.4931 |
| 5 | 1.18 | 326.2 | 1.4912 |
| 10 | 1.19 | 317.94 | 1.4899 |
| 15 | 1.19 | 301.03 | 1.4834 |
| 20 | 1.19 | 291.55 | 1.4791 |
| 25 | 1.2 | 274.38 | 1.4766 |
| 30 | 1.2 | 245.12 | 1.4708 |
| 35 | 1.21 | 229.51 | 1.4655 |
| 40 | 1.21 | 218.77 | 1.4577 |
| 45 | 1.22 | 200.36 | 1.4523 |
| 50 | 1.22 | 194.45 | 1.4489 |
| 55 | 1.19 | 180.09 | 1.4404 |
| 60 | 1.17 | 158.89 | 1.4391 |
| 65 | 1.12 | 126.54 | 1.4353 |
| 70 | 1.1 | 111.15 | 1.4307 |
| 75 | 1.09 | 89.85 | 1.4287 |
| 80 | 1.07 | 71.64 | 1.4226 |
| 85 | 1.05 | 50.24 | 1.4178 |
| 90 | 0.97 | 28.76 | 1.4143 |
| 95 | 0.88 | 10.08 | 1.3942 |
| 100 | 0.79 | 1.19 | 1.3617 |

Table S7: Physicochemical data for reline/EtOH mixed solvent

| **% EtOH** | **Density g/cm^3^** | **Viscosity** | **Refractive Index** |
| --- | --- | --- | --- |
|  |  | **cP** |  |
| 0 | 1.25 | 650.77 | 1.4725 |
| 5 | 1.25 | 637.46 | 1.4596 |
| 10 | 1.26 | 606.83 | 1.4547 |
| 15 | 1.26 | 566.16 | 1.4495 |
| 20 | 1.27 | 520.72 | 1.4473 |
| 25 | 1.27 | 482.35 | 1.4402 |
| 30 | 1.27 | 440.64 | 1.4331 |
| 35 | 1.28 | 371.42 | 1.4285 |
| 40 | 1.28 | 339.22 | 1.4258 |
| 45 | 1.29 | 293.81 | 1.4211 |
| 50 | 1.28 | 238.33 | 1.4192 |
| 55 | 1.27 | 209.25 | 1.418 |
| 60 | 1.26 | 178.74 | 1.4144 |
| 65 | 1.24 | 149.89 | 1.4097 |
| 70 | 1.22 | 104.41 | 1.4055 |
| 75 | 1.21 | 98.86 | 1.3994 |
| 80 | 1.2 | 80.01 | 1.3977 |
| 85 | 1.18 | 57.37 | 1.3935 |
| 90 | 1.12 | 29.45 | 1.3891 |
| 95 | 1.03 | 11.32 | 1.3786 |
| 100 | 0.79 | 1.19 | 1.3617 |

Table S8: S_max_ as a function of % EtOH for all hybrid solvents

| **% EtOH** | **Ethaline** | **Glyceline** | **Reline** |
| --- | --- | --- | --- |
| 0 | 31.44 | 462.22 | 15.24 |
| 5 | 32.57 | 470.88 | 19.08 |
| 10 | 33.94 | 473.67 | 24.68 |
| 15 | 35.28 | 518.94 | 26.33 |
| 20 | 36.54 | 526.83 | 31.95 |
| 25 | 39.43 | 529.64 | 32.76 |
| 30 | 40.16 | 536.10 | 34.43 |
| 35 | 43.11 | 543.65 | 39.40 |
| 40 | 45.10 | 662.41 | 40.02 |
| 45 | 46.22 | 684.77 | 41.96 |
| 50 | 48.42 | 692.07 | 43.52 |
| 55 | 39.36 | 597.04 | 37.67 |
| 60 | 28.83 | 568.77 | 26.63 |
| 65 | 22.46 | 355.64 | 13.85 |
| 70 | 17.44 | 181.76 | 11.62 |
| 75 | 15.07 | 103.31 | 10.40 |
| 80 | 13.32 | 82.17 | 8.48 |
| 85 | 11.34 | 36.41 | 7.59 |
| 90 | 9.22 | 17.03 | 6.99 |
| 95 | 6.90 | 9.79 | 5.70 |
| 100 | 2.26 | 2.26 | 2.26 |

1. Consistency of Tie-Line Data**.**

For this study, the Hand and Othmer – Tobias empirical correlation models was used for consistency testing of the tie lines.

5.1 Othmer-Tobias Equation

Equation S1 was applied to ascertain the reliability of the LLE data. The correlation factors (R^2^) were determined using a partial least square regression. The obtained results from the correlation for the studied systems are reported in Table S9. As seen in the table, the closeness of R^2^ to unity and the linearity of its plot show the good degree of consistency of the measured LLE data for the studied systems.

$ln\{ \frac{1+w_{HC}^{\mathrm{HC}}}{w_{HC}^{\mathrm{HC}}}\}=A+Bln\{ \frac{1+ w_{DES+EtOH}^{DES+EtOH}}{w_{DES+EtOH}^{DES+EtOH}}\}$ S1

Where $w_{HC}^{\mathrm{HC}}$ relates to the mass fraction of hexane in the aliphatic-rich phase and $w_{DES+EtOH}^{DES+EtOH}$relates to the mass fraction of DESs+EtOH in the solvent rich phase, where Aand B are the fitting parameters of Othmer-Tobias.

Table S9: A and B constants of the Othmer-Tobias equation for the studied systems at T =303 K and 101.325kPa

| Systems  n-Hexane + Benzene +[DESs+EtOH] | Othmer Tobias correlation | | |
| --- | --- | --- | --- |
|  | A | B | R^2^ |
| Glyceline + 20%EtOH | -1.8126 | -2.7348 | 0.9956 |
| Glyceline + 40%EtOH | -1.6054 | -2.6175 | 0.9964 |
| Glyceline + 50%EtOH | -1.3283 | -2.5119 | 0.9971 |
| Glyceline + 60%EtOH | -1.2557 | -2.3356 | 0.9979 |
| Glyceline + 80%EtOH | -1.1176 | -2.2071 | 0.9982 |
| Ethaline + 20%EtOH | -1.8673 | -2.6455 | 0.9935 |
| Ethaline + 40%EtOH | -1.7105 | -2.5643 | 0.9943 |
| Ethaline + 50%EtOH | -1.3161 | -2.4062 | 0.9957 |
| Ethaline + 60%EtOH | -1.2884 | -2.2177 | 0.9968 |
| Ethaline + 80%EtOH | -1.1726 | -2.1389 | 0.9973 |
| Reline + 20%EtOH | -1.9225 | -2.5876 | 0.9961 |
| Reline + 40%EtOH | -1.5691 | -2.4065 | 0.9972 |
| Reline + 50%EtOH | -1.3154 | -2.2872 | 0.9979 |
| Reline + 60%EtOH | -1.2298 | -2.1441 | 0.9983 |
| Reline + 80%EtOH | -1.1672 | -2.0757 | 0.9987 |

5.2 Hand Equation

In order to double check the reliability of the experimental tie-line data, Hand equation is also applied. The measured LLE data were fitted by equation S2 using the linear least-square method. The corresponding fitting parameters and correlation factors (R^2^) are listed in Table S10. It can be seen from table S10 that all of the correlation factors (R^2^) are greater than 0.99 and very close to unity. Also, the linearity of the plots indicates the degree of regularity of the measured tie-line data.

Ln$\{\frac{w_{23}}{w_{33}}\}$ = m + n *Ln$\{\frac{w_{21}}{w_{11}}\}$ S2

Where m and n are the fitting parameters of Hands equation,

w_23_ = mass fraction of aromatics in extract phase

w_33_= mass fraction of solvents in the extract phase
w_11_ = mass fraction of aliphatics in raffinate phase

w_21_= mass fraction of aromatics in raffinate phase

Table S10: m and n constants of the Hands equation for the studied systems at T =303 K and 101.325kPa

| Systems  n-Hexane + Benzene +[DESs+EtOH] | Hand correlation | | |
| --- | --- | --- | --- |
|  | m | n | R^2^ |
| Glyceline + 20%EtOH | -4.7225 | -2.2164 | 0.9948 |
| Glyceline + 40%EtOH | -4.5488 | -1.9672 | 0.9952 |
| Glyceline + 50%EtOH | -4.0176 | -1.6141 | 0.9955 |
| Glyceline + 60%EtOH | -3.5307 | -1.2733 | 0.9968 |
| Glyceline + 80%EtOH | -3.1192 | -1.0975 | 0.9974 |
| Ethaline + 20%EtOH | -4.5164 | -2.1358 | 0.9923 |
| Ethaline + 40%EtOH | -3.8243 | -1.7786 | 0.9936 |
| Ethaline + 50%EtOH | -2.7451 | -1.2393 | 0.9945 |
| Ethaline + 60%EtOH | -2.3166 | -0.5775 | 0.9957 |
| Ethaline + 80%EtOH | -1.9827 | -0.3167 | 0.9961 |
| Reline + 20%EtOH | -4.3114 | -2.0552 | 0.9952 |
| Reline + 40%EtOH | -3.3658 | -1.5287 | 0.9957 |
| Reline + 50%EtOH | -2.6245 | -1.2193 | 0.9969 |
| Reline + 60%EtOH | -2.2268 | -0.8143 | 0.9973 |
| Reline + 80%EtOH | -1.8512 | -0.2447 | 0.9979 |

1. Thermodynamic modelling

The model development was achieved using ASPEN PLUS V9.0. The DESs+EtOH were selected as pseudo-pure components and the benzene and n-hexane were selected as conventional components. The following [DESs+EtOH] properties were added in the simulator to define the pseudo-component: Density, molecular weight, vapour pressure, normal boiling temperature, pressure, viscosity.
The NRTL and UNIQUAC models were used to correlate the equilibrium systems.

The UNIQUAC structural parameters [Vander waals group volume (r) and surface area(q)] for the components were obtained and listed in Table S11. All the corresponding binary model parameters were acquired by minimizing the objective function (OF) as shown in equation S3

$OF=\sum_{k=1}^{M} \sum_{s=1}^{2} \sum_{i=1}^{3} (w_{isk}^{exp}- w_{isk}^{cal})$^2^ S3

Where M is the number of tie-lines and w is the mass fraction of component i. The subscripts i, s, and k refer to the components, the phases, and the tie-line, respectively, and the superscripts exp and cal denote the experimental and calculated values, respectively.

For evaluating the correlation accuracy, the root-mean-square deviation (rmsd) was calculated to judge the difference between the calculated data and experimental data and was defined as follows in Equation S4

RMSD = 100 { $\sum_{k=1}^{M} \sum_{s=1}^{2} \sum_{i=1}^{3} \frac{(w_{isk}^{exp}- w_{isk}^{cal})2}{6M}\}$^½^ S4

Where M is the number of tie-lines and w is the mass fraction of component i. The subscripts i, s, and k refer to the components, the phases, and the tie-line, respectively, and the superscripts exp and cal denote the experimental and calculated values, respectively.

The values of RMSD were calculated and are listed in Table S12. As can be seen from the table, the largest value is 0.0306, which indicates that both NRTL and UNIQUAC models can fit the experimental data well, the smaller RMSDvalues indicate that both the NRTL and UNIQUAC models show good relationship for the phase behaviour of studied systems.
 From the results, the NRTL model shows a slightly better result than the UNIQUAC model for the ternary systems of Benzene+ Hexane +[DESs+EtOH].

Table S11: UNIQUAC Structural parameters for n-hexane + benzene + [ DESs + EtOH]

| Components | Van der waals group volume (r) | Surface area (q) |
| --- | --- | --- |
| n-Hexane | 4.4997 | 3.856 |
| Benzene | 3.1905 | 2.400 |
| Glyceline + 20%EtOH | 4.5021 | 3.913 |
| Glyceline + 40%EtOH | 3.8253 | 3.316 |
| Glyceline + 50%EtOH | 3.5281 | 3.124 |
| Glyceline + 60%EtOH | 3.1509 | 2.776 |
| Glyceline + 80%EtOH | 3.1278 | 2.684 |
| Ethaline + 20%EtOH | 3.3213 | 3.163 |
| Ethaline + 40%EtOH | 3.2916 | 2.897 |
| Ethaline + 50%EtOH | 3.2709 | 2.786 |
| Ethaline + 60%EtOH | 3.1974 | 2.623 |
| Ethaline + 80%EtOH | 2.4766 | 2.236 |
| Reline + 20%EtOH | 3.8335 | 3.261 |
| Reline + 40%EtOH | 3.5109 | 3.178 |
| Reline + 50%EtOH | 3.3445 | 2.967 |
| Reline + 60%EtOH | 3.1774 | 2.876 |
| Reline + 80%EtOH | 2.4886 | 2.241 |

**Table S12: Binary Interaction Parameters of NRTL and UNIQUAC Models for the Systems of n-Hexane (1) + Benzene (2) + [DESs + EtOH] (3) at 303 K and 101.325kPa.**

| **components i-j** | **NRTL** | | | | | | | | **UNIQUAC** | | | | | | | | |
| --- | --- | --- | --- | --- | --- | --- | --- | --- | --- | --- | --- | --- | --- | --- | --- | --- | --- |
|  | **a_ij_** | **a_ji_** | **b_ij_(k)** | **b_ji_(k)** | **α** | | **RMSD** | | **a_ij_** | | **a_ji_** | | **b_ij_(k)** | | **b_ji_(k)** | **z** | **RMSD** |
|  |  |  | **n-Hexane +Benzene + [Glyceline + 20%EtOH]** | | | | | | | | | | | |  |  |  |
| **1-2** | **0.4066** | **-1.554** | **-213.735** | **797.572** | **0.3** | | **0.0303** | | **-1.1078** | | **1.2281** | | **294.108** | | **-385.704** | **10** | **0.0306** |
| **1-3** | **0** | **0** | **128.834** | **-114.781** |  | |  |  | **0** | | **0** | | **54.5302** | | **-60.2226** |  |  |
| **2-3** | **0** | **0** | **276.749** | **-74.4868** |  | |  |  | **0** | | **0** | | **11.2081** | | **-61.0081** |  |  |
|  |  |  | **n-Hexane +Benzene + [Glyceline + 40%EtOH]** | | | | | | | | | | | |  |  |  |
| **1-2** | **0.4066** | **-1.554** | **-213.735** | **797.572** | **0.3** | | **0.0292** | | **-1.1078** | | **1.2281** | | **294.108** | | **-385.704** | **10** | **0.0295** |
| **1-3** | **0** | **0** | **107.055** | **-99.8932** |  | |  | | **0** | | **0** | | **54.5299** | | **-60.1234** |  |  |
| **2-3** | **0** | **0** | **198.677** | **-10.5512** |  | |  | | **0** | | **0** | | **11.1949** | | **-60.9922** |  |  |
|  |  |  | **n-Hexane +Benzene + [Glyceline + 50%EtOH]** | | | | | | | | | | | |  |  |  |
| **1-2** | **0.4066** | **-1.554** | **-213.735** | **797.572** | | **0.3** | | **0.0278** | | **-1.1078** | | **1.2281** | | **294.108** | **-385.704** | **10** | **0.0281** |
| **1-3** | **0** | **0** | **245.591** | **-210.984** | |  | |  | | **0** | | **0** | | **54.3315** | **-61.2219** |  |  |
| **2-3** | **0** | **0** | **103.142** | **73.1128** | |  | |  | | **0** | | **0** | | **11.1829** | **-60.9698** |  | **`** |
|  |  |  | **n-Hexane +Benzene + [Glyceline + 60%EtOH]** | | | | | | | | | | | |  |  |  |
| **1-2** | **0.4066** | **-1.554** | **-213.735** | **797.572** | | **0.3** | | **0.0282** | | **-1.1078** | | **1.2281** | | **294.108** | **-385.704** | **10** | **0.0286** |
| **1-3** | **0** | **0** | **276.187** | **-323.489** | |  | |  | | **0** | | **0** | | **53.3767** | **-61.8768** |  |  |
| **2-3** | **0** | **0** | **211.423** | **99.8124** | |  | |  | | **0** | | **0** | | **11.1761** | **-59.6495** |  |  |
|  |  |  | **n-Hexane +Benzene + [Glyceline + 80%EtOH]** | | | | | | | | | | | |  |  |  |
| **1-2** | **0.4066** | **-1.554** | **-213.735** | **797.572** | | **0.3** | | **0.0243** | | **-1.1078** | | **1.2281** | | **294.108** | **-385.704** | **10** | **0.0245** |
| **1-3** | **0** | **0** | **215.343** | **-190.482** | |  | |  | | **0** | | **0** | | **52.7864** | **-62.2463** |  |  |
| **2-3** | **0** | **0** | **101.214** | **67.3173** | |  | |  | | **0** | | **0** | | **10.9629** | **-59.7556** |  |  |
|  |  |  | **n-Hexane +Benzene + [Ethaline + 20%EtOH]** | | | | | | | | | | | | |  |  |
| **1-2** | **0.4066** | **-1.554** | **-213.735** | **797.572** | | **0.3** | | **0.0186** | | **-1.1078** | | **1.2281** | | **294.108** | **-385.704** | **10** | **0.0188** |
| **1-3** | **0** | **0** | **127.035** | **-98.8923** | |  | |  | | **0** | | **0** | | **52.354** | **-58.5225** |  |  |
| **2-3** | **0** | **0** | **208.677** | **-11.5512** | |  | |  | | **0** | | **0** | | **11.2499** | **-62.2928** |  |  |
|  |  |  | **n-Hexane +Benzene + [Ethaline + 40%EtOH]** | | | | | | | | | | | | |  |  |
| **1-2** | **0.4066** | **-1.554** | **-213.735** | **797.572** | | **0.3** | | **0.0155** | | **-1.1078** | | **1.2281** | | **294.108** | **-385.704** | **10** | **0.0159** |
| **1-3** | **0** | **0** | **253.921** | **115.124** | |  | |  | | **0** | | **0** | | **52.3293** | **-59.2217** |  |  |
| **2-3** | **0** | **0** | **113.124** | **83.118** | |  | |  | | **0** | | **0** | | **11.3656** | **-62.9698** |  |  |

| **components i-j** | **NRTL** | | | | | | | | **UNIQUAC** | | | | | | | | |
| --- | --- | --- | --- | --- | --- | --- | --- | --- | --- | --- | --- | --- | --- | --- | --- | --- | --- |
|  | **a_ij_** | **a_ji_** | **b_ij_(k)** | **b_ji_(k)** | **α** | | **RMSD** | | **a_ij_** | | **a_ji_** | | **b_ij_(k)** | | **b_ji_(k)** | **z** | **RMSD** |
|  |  |  | **n-Hexane +Benzene + [Ethaline + 50%EtOH]** | | | | | | | | | | | |  |  |  |
| **1-2** | **0.4066** | **-1.554** | **-213.735** | **797.572** | **0.3** | | **0.0148** | | **-1.1078** | | **1.2281** | | **294.108** | | **-385.704** | **10** | **0.0152** |
| **1-3** | **0** | **0** | **293.195** | **-238.489** |  | |  |  | **0** | | **0** | | **53.2549** | | **-59.7667** |  |  |
| **2-3** | **0** | **0** | **-7.9276** | **-103.241** |  | |  |  | **0** | | **0** | | **11.2172** | | **-61.6989** |  |  |
|  |  |  | **n-Hexane +Benzene + [Ethaline + 60%EtOH]** | | | | | | | | | | | |  |  |  |
| **1-2** | **0.4066** | **-1.554** | **-213.735** | **797.572** | **0.3** | | **0.0142** | | **-1.1078** | | **1.2281** | | **294.108** | | **-385.704** | **10** | **0.0295** |
| **1-3** | **0** | **0** | **349.159** | **-282.489** |  | |  | | **0** | | **0** | | **52.4193** | | **-59.0347** |  |  |
| **2-3** | **0** | **0** | **113.241** | **76.1821** |  | |  | | **0** | | **0** | | **11.3752** | | **-62.7167** |  |  |
|  |  |  | **n-Hexane +Benzene + [Ethaline + 80%EtOH]** | | | | | | | | | | | |  |  |  |
| **1-2** | **0.4066** | **-1.554** | **-213.735** | **797.572** | | **0.3** | | **0.0128** | | **-1.1078** | | **1.2281** | | **294.108** | **-385.704** | **10** | **0.0131** |
| **1-3** | **0** | **0** | **438.362** | **-339.572** | |  | |  | | **0** | | **0** | | **52.5274** | **-60.2198** |  |  |
| **2-3** | **0** | **0** | **-9.2988** | **181.33** | |  | |  | | **0** | | **0** | | **11.4754** | **-60.9356** |  | **`** |
|  |  |  | **n-Hexane +Benzene + [Reline + 20%EtOH]** | | | | | | | | | | | |  |  |  |
| **1-2** | **0.4066** | **-1.554** | **-213.735** | **797.572** | | **0.3** | | **0.0222** | | **-1.1078** | | **1.2281** | | **294.108** | **-385.704** | **10** | **0.0224** |
| **1-3** | **0** | **0** | **107.055** | **-99.8932** | |  | |  | | **0** | | **0** | | **54.9291** | **-59.9252** |  |  |
| **2-3** | **0** | **0** | **198.677** | **-10.5512** | |  | |  | | **0** | | **0** | | **11.2194** | **-61.0923** |  |  |
|  |  |  | **n-Hexane +Benzene + [Reline + 40%EtOH]** | | | | | | | | | | | |  |  |  |
| **1-2** | **0.4066** | **-1.554** | **-213.735** | **797.572** | | **0.3** | | **0.0193** | | **-1.1078** | | **1.2281** | | **294.108** | **-385.704** | **10** | **0.0196** |
| **1-3** | **0** | **0** | **233.342** | **-192.498** | |  | |  | | **0** | | **0** | | **53.9371** | **-59.6124** |  |  |
| **2-3** | **0** | **0** | **173.528** | **75.518** | |  | |  | | **0** | | **0** | | **11.4324** | **-61.6986** |  |  |
|  |  |  | **n-Hexane +Benzene + [Reline + 50%EtOH]** | | | | | | | | | | | | |  |  |
| **1-2** | **0.4066** | **-1.554** | **-213.735** | **797.572** | | **0.3** | | **0.0174** | | **-1.1078** | | **1.2281** | | **294.108** | **-385.704** | **10** | **0.0179** |
| **1-3** | **0** | **0** | **248.357** | **-177.623** | |  | |  | | **0** | | **0** | | **53.6158** | **-58.7212** |  |  |
| **2-3** | **0** | **0** | **201.423** | **82.2364** | |  | |  | | **0** | | **0** | | **11.7692** | **-62.4784** |  |  |
|  |  |  | **n-Hexane +Benzene + [Reline + 60%EtOH]** | | | | | | | | | | | | |  |  |
| **1-2** | **0.4066** | **-1.554** | **-213.735** | **797.572** | | **0.3** | | **0.0141** | | **-1.1078** | | **1.2281** | | **294.108** | **-385.704** | **10** | **0.0148** |
| **1-3** | **0** | **0** | **257.426** | **-172.315** | |  | |  | | **0** | | **0** | | **53.1484** | **-57.6638** |  |  |
| **2-3** | **0** | **0** | **218.274** | **83.7553** | |  | |  | | **0** | | **0** | | **12.1079** | **-63.9464** |  |  |
|  |  |  | **n-Hexane +Benzene + [Reline + 80%EtOH]** | | | | | | | | | | | | |  |  |
| **1-2** | **0.4066** | **-1.554** | **-213.735** | **797.572** | | **0.3** | | **0.0109** | | **-1.1078** | | **1.2281** | | **294.108** | **-385.704** | **10** | **0.0113** |
| **1-3** | **0** | **0** | **422.236** | **-343.645** | |  | |  | | **0** | | **0** | | **52.6472** | **-60.7887** |  |  |
| **2-3** | **0** | **0** | **-9.5879** | **187.437** | |  | |  | | **0** | | **0** | | **12.4748** | **-64.5293** |  |  |

1. Calibration curve for composition measurement


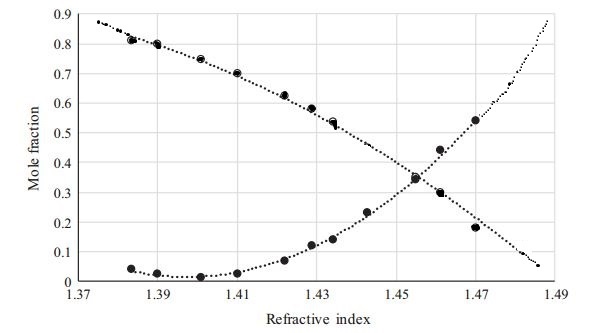


Figure S4: Calibration curve for n-Hexane + Benzene + [Glyceline+EtOH]


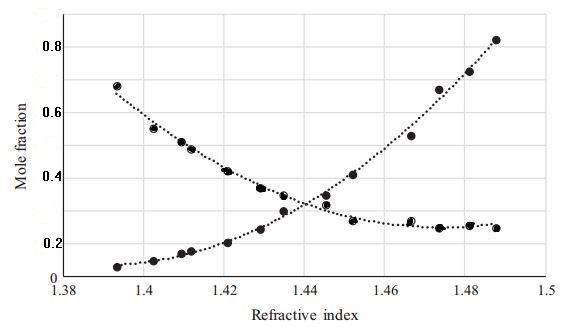


Figure S5: Calibration curve for n-Hexane + Benzene + [Ethaline+EtOH]


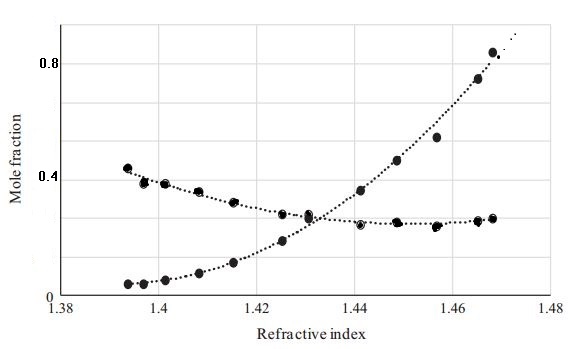


Figure S6: Calibration curve for n-Hexane + Benzene + [Reline+EtOH]
